# Cholinergic-dependent dopamine signals in mouse dorsomedial striatum are regulated by frontal but not sensory cortices

**DOI:** 10.1038/s41467-026-77168-x

**Published:** 2026-09-02

**Authors:** Hannah C. Goldbach, Rachele Rimondini, Evan S. Swanson, Jung Hoon Shin, Michael E. Authement, Lucy G. Anderson, Han Bin Kwon, Ron Paletzki, Charles R. Gerfen, Linda M. Amarante, Richard J. Krauzlis, Veronica A. Alvarez

**Affiliations:** 1 Section on Neurobiology of Compulsive Behaviors, National Institute of Mental Health, Bethesda, MD USA; 2 Laboratory on Neurobiology of Compulsive Behaviors, National Institute on Alcohol Abuse and Alcoholism, Rockville, MD USA; 3 Laboratory of Sensorimotor Research, National Eye Institute, Bethesda, Maryland USA; 4 Center on Compulsive Behaviors, National Institutes of Health, Bethesda, Maryland USA; 5 Section on Neuroanatomy, National Institute of Mental Health, Bethesda, Maryland USA

**Keywords:** Neural circuits, Sensorimotor processing

## Abstract

Everyday decisions depend on associations between sensory stimuli, actions, and outcomes. The striatum supports these sensorimotor associations through dopamine-dependent plasticity. Recent work has characterized a local striatal microcircuit in which cholinergic interneurons (CINs) modulate dopamine release via activation of nicotinic receptors on dopamine axons. Here, we show that visual stimuli evoke dopamine in the dorsomedial striatum partly through this cholinergic mechanism. Using anatomical and functional methods to identify the pathways involved, we found that visual and auditory cortices lack connectivity with CINs and were unable to drive cholinergic-dependent dopamine release. Frontal regions, which were activated by visual stimuli, strongly recruited CINs, producing robust dopamine release both ex vivo and in vivo. These findings reveal a fundamental distinction between sensory and frontal corticostriatal inputs, demonstrating that only the latter can evoke cholinergic-dependent dopamine signals. This work establishes a framework for understanding how cortical circuits shape striatal dopamine to support reinforcement learning.

## Abstract (web summary)

Neural mechanisms underlying sensory-guided learning are not fully understood. Here authors show that salient visual stimuli elicit striatal dopamine partly through a local cholinergic mechanism and identify cortical areas driving cholinergic-dependent dopamine release in vivo and in vitro in the dorsomedial striatum.

## Introduction

Sensory input provides a nearly constant flux of information that guides our decisions about actions, based on expected outcomes. However, the complex pathways through which sensory information drives action remain unclear. The dorsomedial striatum is one brain area known to play a role in this process^1–4^. The dorsomedial striatum receives glutamatergic input from the sensory cortex, frontal cortex, and thalamic nuclei, as well as dopaminergic input from the midbrain. It is this integration of contextual sensory information with expected outcomes, signaled largely by dopamine, that enables the dorsomedial striatum’s function in guiding goal-directed behaviors, sensory learning, and attention. Historically, most mechanistic understanding has focused on how striatal medium spiny neurons (MSNs), which comprise the main output pathways of the striatum, are driven by glutamatergic inputs and modulated by dopamine^5,6^. The temporal alignment of these glutamatergic and dopamine signals is crucial as it determines the rules of synaptic plasticity, promoting either long-term potentiation or depression of selective inputs^7–9^ to reshape functional connectivity and promote the associative learning of sensory cues and motor actions^10–13^.

Midbrain-originating action potentials have long been thought to have sole control over the timing of striatal dopamine signals, so much so that midbrain neuron activity is commonly interpreted as a proxy for dopamine release in the striatum. However, an alternative mechanism has recently been identified, in which acetylcholine released by cholinergic interneurons (CINs) can act locally within the striatum to regulate dopaminergic axonal excitability and dopamine release through nicotinic acetylcholine receptors (nAChRs)^14–21^, independently of soma-generated action potentials^22,23^. This local regulatory mechanism does not reflect the activity of CINs alone. Instead, CIN-evoked dopamine appears to act as a coincidence detector by requiring synchronized recruitment by the glutamatergic inputs arriving from the cortex and thalamus^17^.

Striatal CINs produce a characteristic response to novel or salient stimuli, which can strengthen as an animal learns to associate those stimuli with actions and rewards. This has been observed both in non-human primates^24–27^ and, more recently, in mice^28,29^. The recently identified ability of CINs to locally regulate dopamine opens new possibilities about the neural circuits underlying these behavioral findings. Theoretically, cortical and thalamic projections to the striatum that recruit CINs could regulate the temporal and spatial distribution of dopamine signals to shape behavior and learned associations with sensory stimuli. In support of a physiological role for this local mechanism, a recent study demonstrated that cholinergic control over dopamine in the ventral striatum contributes to effortful behavior^30^. Recent experiments have shown that motor and somatosensory cortical inputs, as well as thalamic inputs, synapse onto MSNs with more strength than they do onto CINs^31,32^. Yet we do not know which glutamatergic inputs drive striatal CINs to provide cholinergic control over dopamine, nor how it compares across subregions of the striatum. Recent in vivo evidence suggests that CIN responses to sensory cues are shaped by the convergence of top-down inputs from the cortex with bottom-up inputs from the thalamus and midbrain^33^. Therefore, CINs might play an integrative role in the formation of associations for sensory-guided actions, whereby brain regions with functional connections to CINs can synchronize acetylcholine release to gain temporal and spatial control over dopamine transmission.

These findings raise new fundamental questions about which inputs to the dorsomedial striatum recruit CINs to regulate dopamine transmission. Motor cortex^18^, prelimbic cortex^20^, and some thalamic nuclei^18,34^ can evoke striatal dopamine through recruitment of CINs. However, it is unknown whether sensory cortical areas, which project robustly to the dorsomedial striatum, can also recruit CINs directly. Specifically, if sensory cortices could also recruit CINs and control the timing of dopamine signals, this would allow direct control over action selection and the learned associations between stimuli and rewards. In contrast, if the ability to drive striatal dopamine through CINs is unique to a few areas—such as the prefrontal cortex and thalamus—it would indicate a larger role for feedforward pathways through the frontal cortex and sensory thalamic nuclei in sensory-guided learning.

Here we show that, in fact, salient visual stimuli elicit striatal dopamine in part via a cholinergic-dependent mechanism. We identify specific cortical areas and striatal cell types involved in driving cholinergic-dependent dopamine release in the dorsomedial striatum. In vivo fiber photometry and ex vivo recordings revealed that prefrontal areas connect far more heavily with CINs in the dorsomedial striatum than cortical sensory areas such as the visual and auditory cortices, despite their prominent projections to striatal medium spiny neurons in the dorsomedial striatum. Cortical sensory areas failed to drive cholinergic-dependent dopamine in the dorsomedial striatum and formed only rare and weak synaptic contacts with CINs, as validated by anatomical and functional analysis. Lastly, we confirmed that visual stimuli drive in vivo time-locked calcium responses in striatal-projecting frontal cortical axons and stimulation of these corticostriatal projections recruits CINs to drive acetylcholine and dopamine release in the dorsomedial striatum.

Through this combination of experimental approaches, our findings establish that frontal cortices, but not sensory cortices, can play a prominent role in controlling striatal CINs and modulating striatal dopamine. These results complement other findings demonstrating the importance of frontal cortices in sensorimotor learning and dopamine transmission, and highlight the importance of these additional circuit details as an unexplored avenue for understanding the etiology of dopamine-related disorders.

## Results

### Salient visual stimuli elicit striatal dopamine through both midbrain and local cholinergic mechanisms

**Fig. 1:**
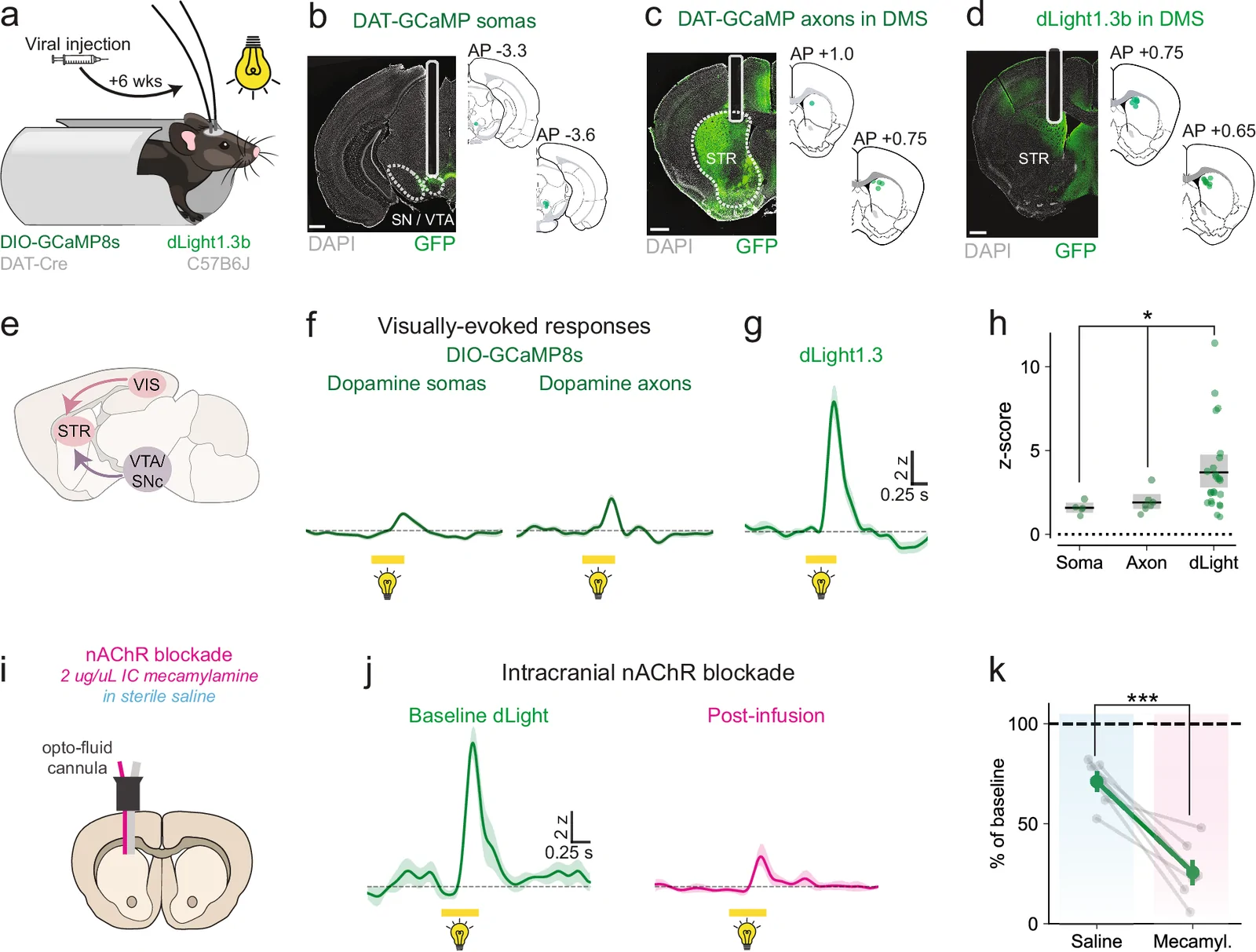
Salient sensory stimuli trigger nAChR-dependent striatal dopamine. **a** Experimental setup of head-fixed mice presented with white light (500 ms, 5.9 cd/m^2^). Mice were held in a dark sound-attenuating box and stimuli presentation spaced roughly every 60 s. Intracranial surgeries were carried out at least 6 weeks in advance. **b**–**d** Representative images of immuno-fluorescence from DAT-IRES-Cre mice expressing GCaMP8s in midbrain dopamine neurons with fiber implants in midbrain and DMS (**b**, **c**); or wildtype mice expressing dLight1.3b in DMS with fiber implants in DMS (**d**). Scale bars are 500 µm. Fiber placements are displayed on Allen Brain Atlas plates73. **e** Diagram depicts a canonical pathway (purple) for dopamine signals in the striatum generated from the ventral tegmental area (VTA) and substantia nigra compacta (SNc). An Additional putative pathway (pink) for the generation of nAChR-dependent striatal dopamine in response to visual stimuli. **f**,** g** Average example traces of visual stimulus evoked calcium signals (f) from midbrain fibers (dopamine neuron somas) and DMS fiber (axons) and dLight1.3 dopamine signals in DMS. **h** Maximum z-scored for evoked GCaMP8 (soma *n* = 5, axon *n* = 7) and dLight1.3 (*n* = 25) responses to light cue presentation (*p* = 0.04, one-way ANOVA). Each dot represents a mouse, black lines represent medians, and gray error bars represent the bootstrap 95% CI. **i** A subset of dLight-expressing mice were implanted with an optofluid cannula and received intracranial infusions of saline (vehicle) or mecamylamine (0.2 µg / µL). **j** Single session traces from a representative animal showing the visually-evoked dopamine response before (baseline) and after (magenta) intracranial infusion of mecamylamine. **k** Across-animal effects of intracranial saline and mecamylamine on visually-evoked dopamine responses (*p* = 0.0007, RM 2-way ANOVA). Amplitudes were normalized to baseline responses before infusion (*n* = 7 mice). Gray dots are individual animals, and green dots represent the mean +/– sem. For all traces, line and shade represent mean z-score ± sem.

We used fiber photometry to survey dopamine release and dopamine neuron activity in response to salient visual stimuli (Fig. 1a, e, “Methods”). DAT-cre mice expressing cre-dependent GCaMP8s in midbrain dopaminergic neurons were implanted with two fiber optic cannulae: one in the midbrain to measure somatic dopamine neuron activity, and another in the dorsomedial striatum (DMS) to measure axonal dopamine neuron activity (Fig. 1b, c). A separate group of mice, expressing the dopamine sensor dLight1.3b in DMS, were implanted with fiber optic cannulae in the DMS to measure striatal dopamine (Fig. 1d). All mice were implanted with a headframe and habituation to the headfixed chamber. Mice passively experienced visual stimuli (LED light, 5.9 cd/m^2^, 500 ms duration) delivered at random intervals averaging 60 s. Calcium responses time-locked to visual stimuli were recorded from both the somas and the axons of the dopamine neurons (Fig. 1f, h; mean z-score in somas 1.58 [1.28 − 1.88 bootstrap 95% CI], *n* = 5 animals; mean in axons 1.90 [1.53 − 2.39], *n* = 7 animals). Within the DMS, these same visual stimuli elicited a robust dopamine signal time-locked to stimulus onset, measured as large transients of dLight1.3b fluorescence (Fig. 1g, h; mean z-score 3.70 [1.53 − 2.39], *n* = 25 animals; significant effect of sensor and recording location *p* = 0.04, one-way ANOVA).

Given recent reports that striatal acetylcholine may amplify or regulate striatal dopamine^21,35–37^, we explored whether these visually-evoked dopamine signals were dependent on nicotinic receptor activation. We first tested the effect of systemic administration of mecamylamine, a non-competitive nicotinic receptor antagonist. We found that mecamylamine (10 mg/kg, i.p.) reduced visual-evoked dopamine signals to 41 ± 4.8% of baseline, while sparing the somatic calcium responses in dopamine neurons (Supplementary Fig. 1). These results from systemic drug administration hinted at a cholinergic contribution but could not distinguish between a striatal site of action and peripheral effects. To directly test the effects of mecamylamine in the striatum, we repeated the dopamine sensor experiments using an opto-fluid cannula that allows for intracranial infusion within the recorded area of mecamylamine (2 µg / µL) or vehicle (saline) (Fig. 1i). The effects were even larger – intracranial mecamylamine significantly decreased visually-evoked dopamine responses to 26 ± 5% (Fig. 1j-k; *n* = 7 animals; treatment *p* < 0.0001, one-way repeated-measures ANOVA).

**Fig. 2:**
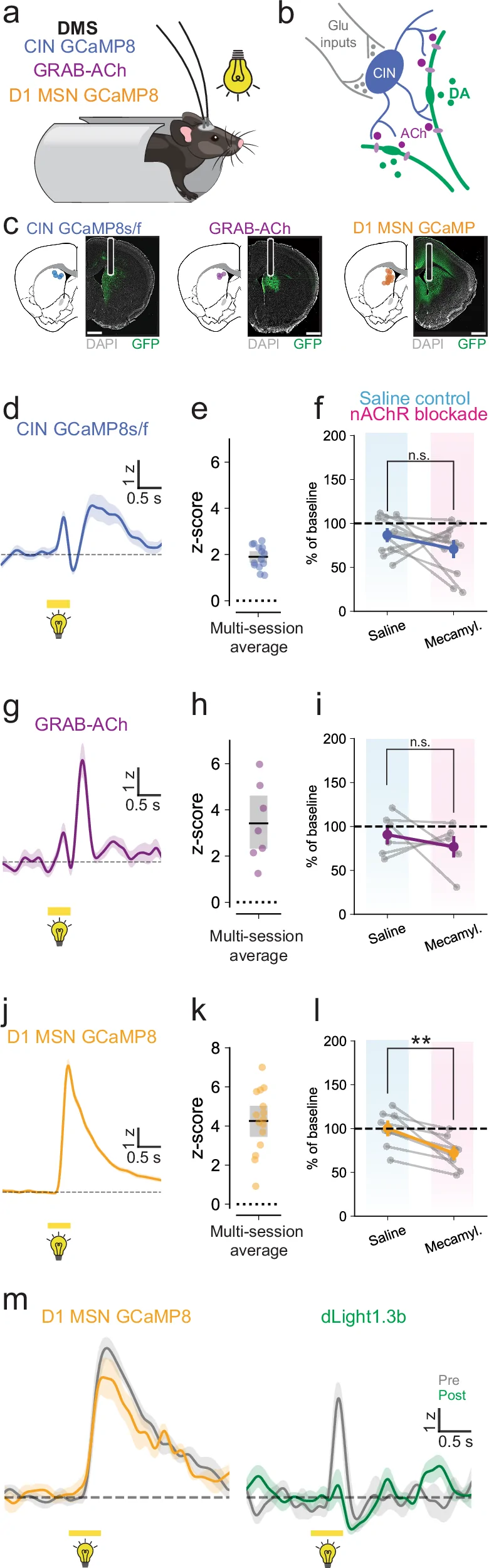
Visual stimuli recruit striatal CINs and drive acetylcholine responses. **a** Headfixed setup for light stimulus presentation (5.9 cd/m^2^, 500 ms). All mice underwent surgery at least 6 weeks in advance and received fiber implants in DMS. ChAT-IRES-Cre and Drd1-Cre mice were used to express GCaMP in CINs or D1-MSNs, respectively. Wildtype C57Bl-6J or ChAT-IRES-cre mice expressed GRAB-ACh in the dorsomedial striatum. **b** Diagram of the striatal microcircuitry possibly involved: cholinergic interneurons (blue) are recruited by glutamatergic inputs to the striatum (gray) and release acetylcholine (purple), which activates nAChRs on DA axons (green) to produce local dopamine release. **c** Fiber placements and histology from representative mice expressing GCaMP8 in CINs, GRAB-ACh, or GCaMP8 in D1-MSNs. Scale bars are 1 mm. Fiber placements are displayed on Allen Brain Atlas plates73. **d**,** g**,** j** Single-animal traces of GCaMP responses in CINs (**d**), GRAB-ACh (**g**), and GCaMP responses in D1-MSNs (**j**) aligned to light onset. Lines and shades are mean z-score ± sem. **e**,** h**,** k** Maximum z-score of visual-evoked responses for GCaMP in CINs (**e**, *n* = 12 mice), GRAB-ACh (**h**, *n* = 7 mice), and GCaMP in D1-MSNs (**k**, *n* = 9 mice). Each dot represents one animal. Black lines represent means, and gray error bars represent bootstrapped 95% CI. **f**,** i**,** l** Normalized effect of saline and nAChR antagonist mecamylamine (10 mg/kg, i.p.) on in vivo responses to light onset in mice expressing GCaMP in CINs (**f**, *n* = 11 mice; *p* = 0.21, RM 2-way ANOVA), GRAB-ACh (**i**, *n* = 6 mice; *p* = 0.43, RM 2-way ANVOA) or GCaMP in D1-MSNs (**k**, *n* = 8 mice; *p* = 0.004, RM 2-way ANOVA). Gray symbols and lines are paired data from individual hemispheres pre- and post-administration of saline (blue) or mecamylamine (pink). Black lines and shaded error bars represent the median and bootstrapped 95% CI. **m** Representative traces show visual-evoked responses from an animal expressing GCaMP8 in D1-MSNs in one hemisphere (left) and dLight1.3b in the other hemisphere (right). GCaMP signals in D1-MSN were only slightly reduced by mecamylamine administration, while dLight1.3b signals were dramatically reduced. All traces represent mean z-score ± sem.

We reasoned that a visually evoked, cholinergic-dependent dopamine signal would require the recruitment of striatal cholinergic interneurons (CINs) by visual stimuli. Striatal CINs have been shown to respond to salient sensory stimuli in a series of classical electrophysiology studies done in non-human primates^24,25^. To assess whether the visual stimulus drove striatal CINs and evoked local ACh release in the mouse DMS, we expressed unilateral GCaMP8s/f in CINs and/or unilateral GRAB-ACh3.0, an acetylcholine sensor (Fig. 2a–c, SupplementaryTable S1). Calcium signals from CINs increased in response to visual stimuli (Fig. 2d, e; mean 1.84 z [1.58 − 2.08], *n* = 12 animals), confirming a visual drive. Furthermore, visual stimuli increased acetylcholine in DMS, as measured with GRAB-ACh3.0 (Fig. 2g, h; mean 3.30 z [2.31 − 4.31], *n* = 7 animals). Note that both cue-evoked responses were notably multiphasic and heterogeneous, consisting of one, two, and sometimes even three phases (Supplementary Fig. 2), in line with recent reports^38^. Quantification was limited to the first phase of the response.

In contrast to its effects on dopamine signals, systemic mecamylamine (10 mg/kg, i.p) had no effect on visually-evoked calcium signals in CINs (Fig. 2f; *n* = 11 animals; *p* = 0.21, one-way repeated-measures ANOVA) nor acetylcholine release (Fig. 2i; *n* = 6 animals; *p* = 0.43, one-way repeated-measures ANOVA). This insensitivity to nicotinic receptor blockers is consistent with the candidate circuit mechanism (Fig. 2b), since CIN activity and acetylcholine release lie upstream of the proposed site of action of nACh receptors on the dopamine axon terminals. It also further rules out peripheral actions or even central action on other brain circuits of systemic mecamylamine administration.

As a control, we also confirmed that D1 receptor-expressing MSNs robustly respond to the visual stimulus using the same sensor, GCaMP8s (Fig. 2j, k; mean 4.06 z [2.96 − 5.04] *n* = 9 animals). Calcium signals in D1 MSNs persisted after systemic mecamylamine, though with a small decrease (Fig. 2l, m; *n* = 8 animals; *p* = 0.0035, one-way repeated-measures ANOVA), possibly due to the blunting of the dopamine response by mecamylamine. Together, the fact that cue-evoked responses in D1, CIN, and acetylcholine were largely insensitive to the nAChR blocker is evidence that visual information still reached the striatum and argues against a generalized impairment in visual pathways. Despite the presence of a preserved visual drive, we confirmed a dramatic decrease in visually-evoked dopamine responses during systemic mecamylamine administration (Fig. 2m, right), providing further evidence that activation of nicotinic ACh receptors is a key element underlying the sensory stimuli-evoked dopamine responses.

We also tested auditory responses to determine whether the findings generalized to other sensory modalities. In this anterior portion of the dorsomedial striatum, brief tone presentations (7.5 KHz at 70 dB for 500 ms) failed to produce any reliable responses in dopamine somas or axons, despite robust responses in somas to waterdrop reward delivery (Supplementary Fig. 3). Similarly, tones failed to produce consistent dopamine responses (Supplementary Fig. 4). Auditory cues also failed to trigger acetylcholine transients nor recruit CINs in this anterior DMS (Supplementary Fig. 4). Thus, the sensory-evoked striatal dopamine responses in the anterior DMS may be relatively selective for visual stimuli.

### Unlike frontal cortical areas, primary sensory areas do not synapse onto dorsomedialstriatal CINs

**Fig. 3:**
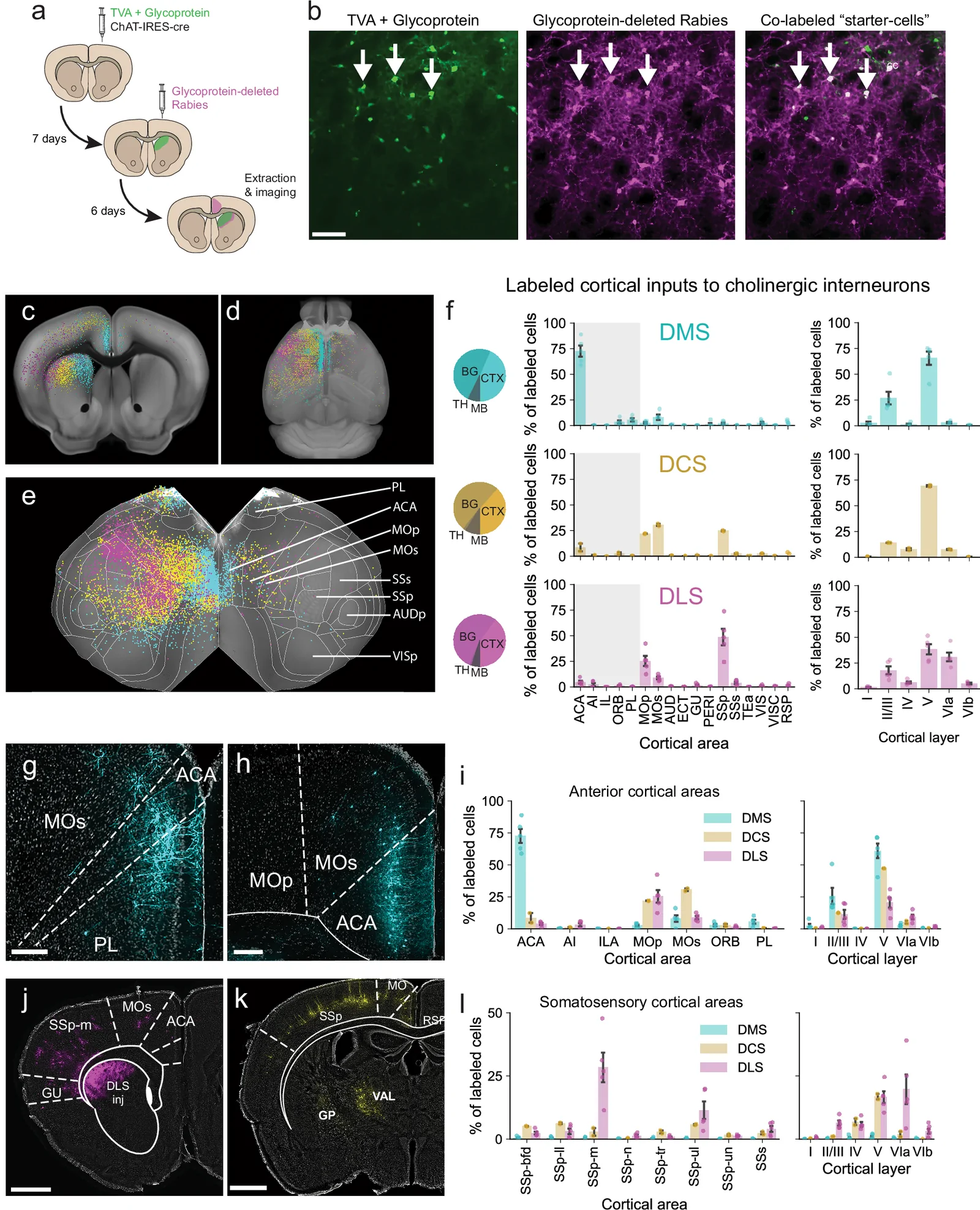
Retrograde rabies tracing demonstrates a widespread anterior bias towards frontal cortical connectivity with striatal cholinergic interneurons. **a** Experimental timeline for injecting ChAT^IRES-cre^ mice with cre-dependent TVA rabies receptor + glycoprotein, followed by a modified glycoprotein-deleted retrograde rabies vector to label CIN projecting cells. **b** Images of a DMS injection area showing cells expressing TVA-glycoprotein (left, green) and glycoprotein-deleted rabies (middle, magenta). Overlaid images (right) show co-labeled cells that serve as “starter” cells (white). **c**, **d**,** e** Reconstructions showing CIN-projecting cortical cells in coronal view (**c**), whole-brain view (**d**), and flattened brain view (**e**) from injections in DMS (cyan), DCS (yellow), and DLS (magenta). **f** Quantifications of CIN-projecting labeled cortical cells per area (left) and per cortical layer (right). Plots show averages of all labeled cortical cells for injection sites in DMS (*n* = 5 mice), DCS (*n* = 2 mice), and DLS (*n* = 5 mice). ACA, anterior cingulate area; PL, prelimbic; MOp, primary motor; MOs, secondary motor; SSp, primary somatosensory. Bar and error bars represent mean ± sem. See Supplementary Table S2 for other anatomical nomenclature. Inset pie charts represent percentages of labeled cells in cortex (CTX), basal ganglia (BG), thalamus (TH), and midbrain (MB). **g**,** h**,** j**, **k** Representative images of retrogradely labeled cortical neurons projecting to striatal CINs in DMS (**g**, **h**), to CINs in DCS (**j**), and to CINs in DLS (**k**) in coronal sections. Inset scale bars are 250 μm. **i**,** l** Quantifications of retrogradely labeled cortical cells per subregion (left) and layer (right) expressed as a percentage of total labeled cortical cells for frontal cortical areas (**i**) and somatosensory areas (**l**). (DMS *n *= 5 mice, DCS *n* = 2 mice, and DLS *n* = 5 mice; bars and error bars represent mean ± sem).

Next, we asked which striatal inputs could drive striatal CINs to promote local acetylcholine and, potentially, dopamine release. Primary sensory areas (visual, somatosensory, and auditory cortex) are known to project to the dorsal striatum. Though known to synapse with MSNs, it is not yet known whether they also synapse onto CINs. To address this, we used cell-type-specific monosynaptic retrograde rabies tracing to widely survey the frequency of cortical connections to striatal CINs in the dorsal striatum. We virally expressed cre-dependent TVA receptors and rabies glycoprotein in striatal CINs of ChAT-cre mice, which allowed a modified glycoprotein-deleted rabies virus to selectively infect cre-expressing CINs (“starter cells”) and label neurons that synapse onto them (Fig. 3a, b, Supplementary Table S1 and Supplementary Fig. 5; Methods). The majority of labeled projection cells were identified in the cortex (49–54%), although labeled cells were also found in the thalamus (5–9%), midbrain (1-2%), and locally within basal ganglia (38–44%; Fig. 3f, insets)

Labeling across the medial to lateral axis in the cortex mirrored injection sites across the same axis in the striatum. Injections in medial, central, and lateral dorsal striatum produced labeling in medial frontal areas, motor cortices, and somatosensory cortex, respectively (Fig. 3c–f). Most of this labeling was found in anterior cortical areas, with only sparsely labeled cells in more posterior areas, including primary visual cortex (VISp) and primary auditory cortex (AUDp, Fig. 3c–f).

Injections in the DMS led to robust labeling in the anterior cingulate area (ACA) with only sparsely labeled cells in sensory cortices (Fig. 3f, left). The majority of cells labeled in the cortex were located in layer 5 (66% of labeled cortical cells), although a smaller percentage were located in layers 2/3 (27%) (Fig. 3f, right), as expected for striatal projecting neurons^39–44^. Of all cortical cells labeled per mouse, an average of 73% were located in ACA (Fig. 3g–i), with additional labeling in the secondary motor cortex (8% of cortical cells, MOs) and prelimbic cortex (6%, PL) (Fig. 3g, i). Very few labeled cells were located in primary somatosensory cortex (SSp, 2%), VISp (3%), or AUDp (0.4%) (Fig. 3f, Supplementary Fig. 2). Injections in the dorsal central striatum (DCS) led to fairly even labeling across primary motor cortex (MOp, 22% of cortical cells), secondary motor cortex (MOs, 31%), and SSp (25%). Meanwhile, injections in the dorsal lateral striatum (DLS) led to labeling in the heaviest expression in SSp (49% of labeled cortical cells) and lesser expression in MOp (25%) and MOs (9%, Fig. 3f, k). Again, the majority of these cells in both DCS and DLS were located in layer 5 (69% and 38%, respectively), with smaller percentages in layer 6 (8% and 31%) and layers 2/3 (14% and 18%) (Fig. 3f).

SSp contains seven topographic sub-regions, each representing a part of the body. When we divided the DLS cell counts further into these sub-regions, we found that roughly half of the labeled SSp cells were located in the mouth/jaw area (SSp-m, 28% out of a total 49%), and one quarter of SSp cells were in the upper limb (SSp-ul, 11% out of 49%, Fig. 3k, l). The barrel field of somatosensory cortex (SSp-bfd) contained only 2% of the labeled cells across the cortex (Fig. 3k, l). In comparison, 3% of all labeled cells were also found across the visual cortex, which also consists of several sub-regions. VISp contained only 0.6% of labeled cells, while secondary visual areas (VISa, VISal, VISam, VISl, VISli, VISpm, and VISrl) contained less than 1% (0.05 − 0.7%) of labeled cells (Supplementary Fig. 2). The auditory cortex contained even fewer labeled cells than the visual cortex, with only 0.03 to 0.2% of cells in each auditory sub-region (Supplementary Fig. 2).

These results demonstrate that only a handful of medial frontal cortical areas synapse frequently with striatal CINs in the dorsomedial striatum.

### Frontal areas form reliable functional connections with striatal CINs, while primary sensory areas do not

**Fig. 4:**
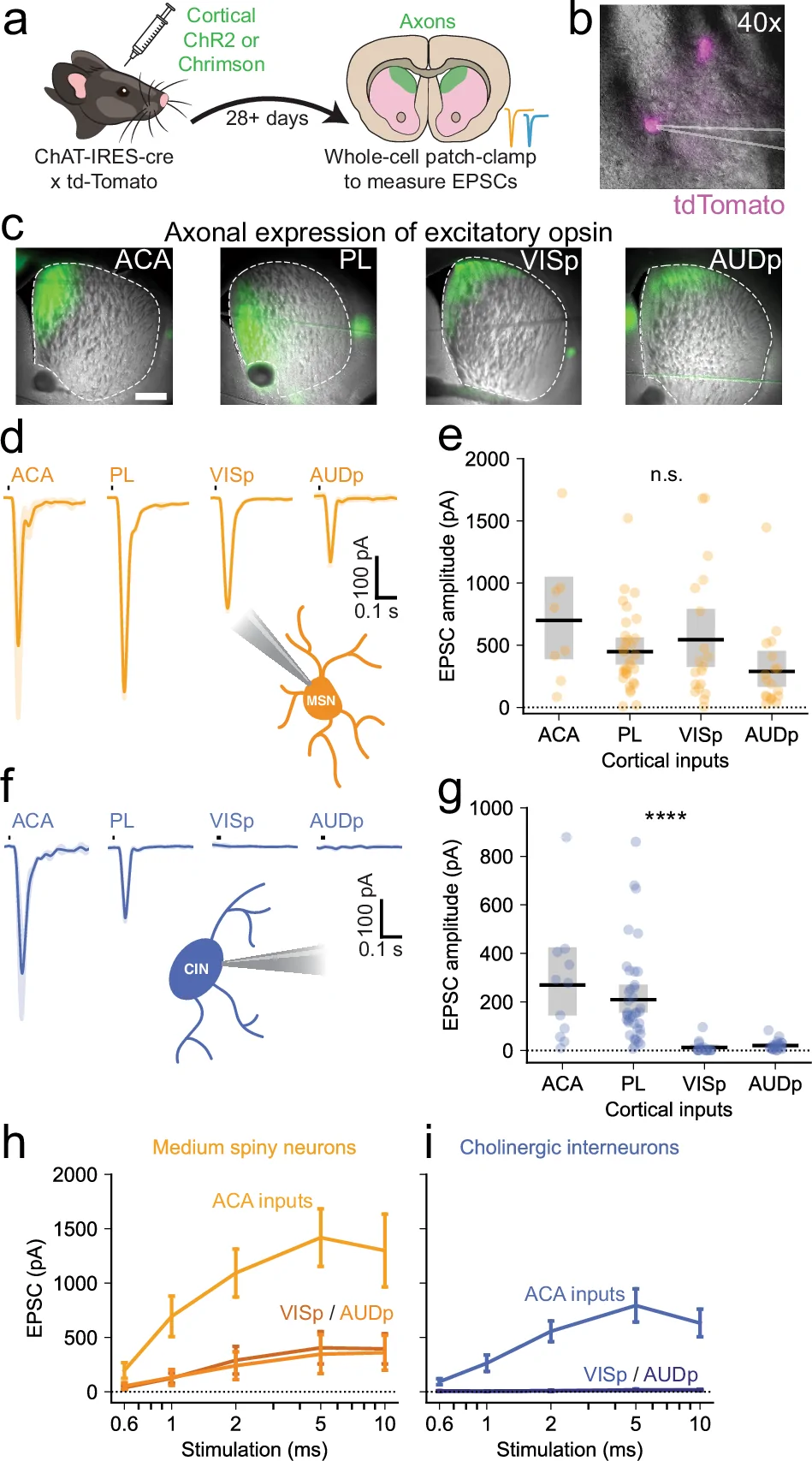
Frontal, but not visual or auditory, cortical areas form reliable functional connections with striatal CIN. **a** Mice with labeled CINs (ChAT-IRES-Cre x tdTomato) were injected with either ChR2-eYFP or ChrimsonR-tdTomato in the anterior cingulate (ACA), prelimbic (PL), visual cortex (VIS) or auditory cortex (AUD). Brains were extracted and sliced for ex vivo whole-cell patch clamp electrophysiology following a minimum four-week incubation. **b** Visualization of genetically encoded tdTomato expression in striatal CINs, as viewed on the electrophysiology rig. **c** Representative images show green fluorescent axonal projections expressing excitatory opsins in DMS, following injections in anterior cingulate (ACA), prelimbic (PL), visual (VISp), or auditory (AUDp) cortices. Scale bar is 500 μm. **d** Representative traces of excitatory postsynaptic currents (EPSC) recorded from medium spiny neurons (MSNs) in DMS in response to optogenetic stimulation of cortical projections. **e** Average MSN EPSC amplitudes evoked via optogenetic stimulation of different cortical area projections (ACA *n* = 11/3; PL *n* = 41/11; VIS *n *= 18/7; AUD *n* = 25/7 - slices/mice; *p* = 0.13, one-way ANOVA). Symbols represent data from individual cells; black lines and shaded error bars represent median and bootstrap 95% CI. **f** Representative EPSC traces recorded from CINs in DMS in response to optogenetic stimulation of cortical area projections. **g** Average CIN EPSC amplitudes evoked via optogenetic stimulation of cortical area projections. (ACA *n* = 8/3; PL* n* = 32/8; VIS *n* = 19/5; AUD *n *= 19/5 - slices/mice; *p* < 0.0001, one-way ANOVA). Symbols represent data from individual cells; black lines and shaded error bars represent median and bootstrap 95% CI. **h**,** i** Input-output curves showing EPSC amplitudes recorded from MSNs (orange) and CINs (blue) in response to increasing duration of optogenetic stimulation of corticostriatal projections. All traces are mean and shaded sem. (MSN: ACA *n* = 8/3, VIS *n *= 7/2, AUD* n* = 8/2; CIN: ACA *n *= 11/3, VIS *n* = 4/2, AUD *n* = 8/2 - slices / mice).

The retrograde rabies tracing identified specific cortical areas with a significant number of cells forming synapses onto striatal CINs, but it did not inform us about the strength of those synapses. We therefore sought to functionally validate the rabies results using ex vivo whole-cell patch-clamp electrophysiology to record excitatory postsynaptic currents (EPSCs) from striatal MSNs and CINs as we optogenetically stimulated different cortical inputs. Mice were injected with ChR2-eYFP or ChrimsonR-tdTomato in either the anterior cingulate cortex, prelimbic cortex, primary somatosensory, visual, or auditory cortex (Methods; Fig. 4a–c). All CIN recordings were conducted using ChAT-tdTomato mice to aid in cell identification, while MSN recordings were conducted using ChAT-tdTomato mice or other cre-recombinase lines on C57B6/J backgrounds (“**Methods**”).

Coronal brain slices showed distinctive patterns of labeled corticostriatal projections in the dorsomedial or dorsolateral striatum, depending on the targeted cortical areas (Fig. 4c). CIN and MSN recordings were made from the striatal region receiving labeled projections. 405 nm blue light (1.3 mW) and 590 nm orange light (0.7 mW) were used to optogenetically excite these terminals, as a subset of these animals expressed both opsins (see below, Supplementary Fig. 6). When recording from MSNs, optogenetic activation (1 ms) of ACA or PL inputs produced large excitatory postsynaptic currents (Fig. 4d, e; ACA: mean 699.4 [382.9 − 1062.8 bootstrap 95% CI] pA, *n* = 8 cells from 3 animals; PL: 448.1 [344.9 − 560.7] pA, *n* = 32 cells from 9 animals). In line with previous findings, sensory areas also produced measurable EPSCs in MSNs (Fig. 4d, e; VISp: 544.0 [327.2 − 792.4] pA, *n* = 19 cells from 5 animals; AUDp: 289.1 [165.4 − 452.6] pA, *n* = 19 cells from 5 animals). The tested cortical regions all formed robust synaptic connections with MSNs, and we found no significant difference in the mean responses (*p* = 0.12, one-way ANOVA).

When recording from CINs, however, we only saw large EPSCs in response to a 10 ms stimulation of ACA or PL (Fig. 4f, g; ACA: 269.6 [143.6 − 423.3] pA, *n* = 11 from 3 animals; PL: 209.4 [155.2 − 270.6] pA, *n* = 41 cells from 12 animals). Stimulation of inputs from primary sensory areas (10 ms) consistently produced little to no response in CINs (Fig. 4f, g**;** VISp: 12.2 [3.8 − 24.2] pA, *n* = 18 cells from 7 animals; AUDp: 20.2 [13.8 − 28.0] pA, *n* = 25 cells from 7 animals). Comparing the strength of the synaptic connections to CINs across the cortical regions revealed significant differences between ACA/Pl and the other sensory areas (*p* < 0.0001, one-way ANOVA). We explored the responses in CIN and MSNs by varying the duration of the stimulation (Fig. 4h, i). When considering the sensory areas, a 2-5 ms stimulation was sufficient to saturate the responses recorded from MSNs (Fig. 4h). Meanwhile, a 10 ms stimulation of sensory terminals failed to produce any response in CINs (Fig. 4i).

As an additional test of the selective connectivity from sensory cortices to MSNs and not CINs, a cohort of mice simultaneously expressed ChrimsonR in a primary sensory area and ChR2 in the frontal cortex (ACA / PL), which served as an internal control. This allowed us to compare how the same cell (a single MSN or single CIN) responded to two different cortical inputs (Supplementary Fig. 6). The paired data confirmed the findings that single CINs responded strongly to inputs from PL, but not any primary sensory area. This data also confirmed that single MSNs receive inputs from both frontal and sensory cortices. This experiment provided the benefit of paired data and internal control, but also restricted our recordings spatially to places with overlapping expression.

Our rabies tracing results showed that the primary somatosensory cortex (SSp) connects with CINs in DLS. We therefore repeated these electrophysiology experiments in DLS with stimulation of SSp terminals. While we did observe that SSp consistently drives MSNs, we did not observe evidence of strong connections between SSp terminals and CINs (Supplementary Fig. 7).

In summary, the ex vivo electrophysiology data functionally confirmed the anatomical findings from Fig. 3. While MSNs received inputs from all areas tested, we observed a heavy bias in connectivity towards frontal cortical areas when recording from CINs.

### Frontal cortical areas, but not sensory, produce local acetylcholine-dependent dopamine release

**Fig. 5:**
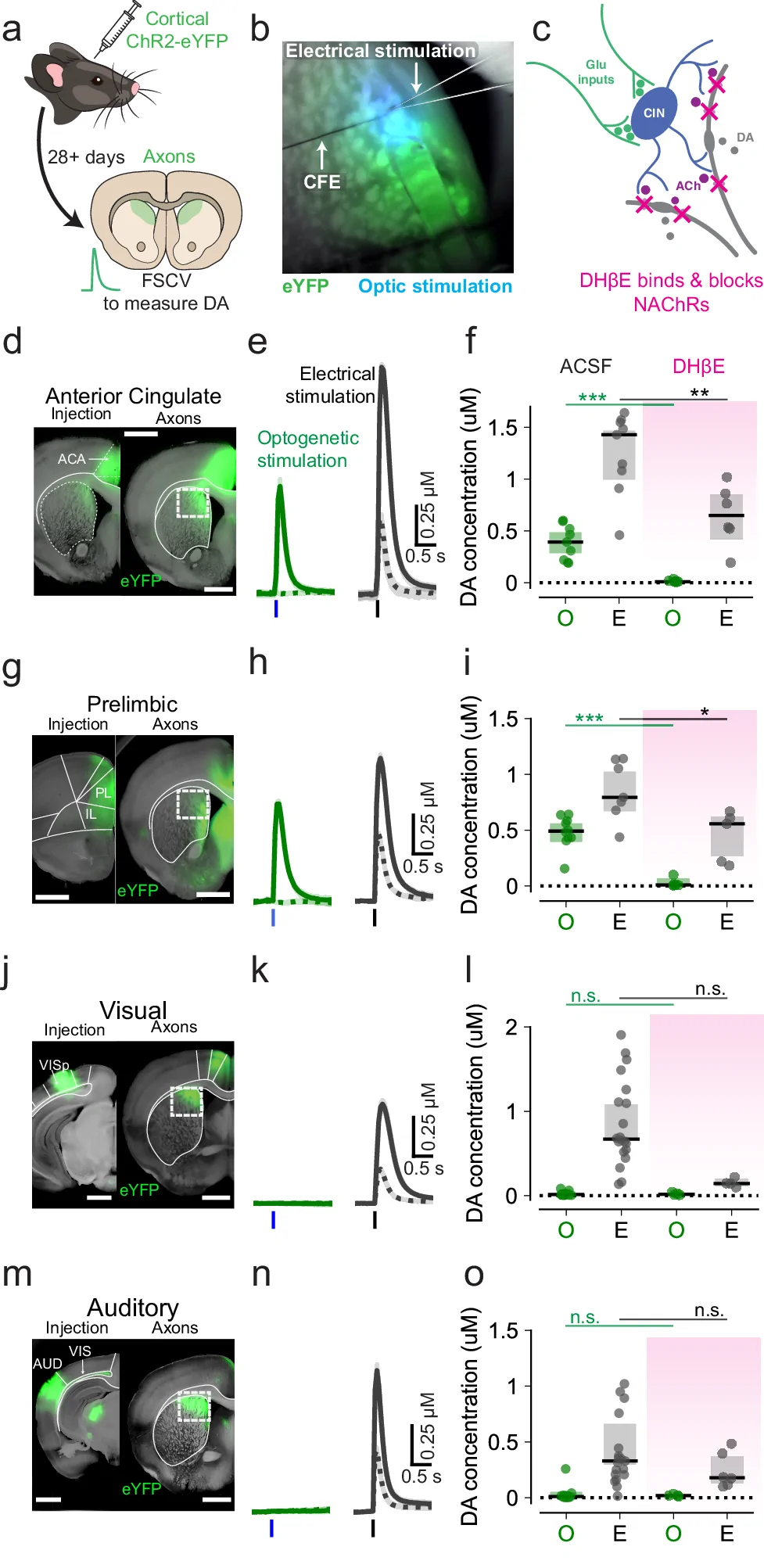
Corticostriatal projections from frontal, but not visual/auditory (sensory), cortical areas can evoke ex vivo local dopamine release in DMS. **a** Mice expressing ChR2-eYFP in frontal or sensory cortical areas were used for ex vivo brain slice recordings of dopamine with fast-scan cyclic voltammetry. **b** Fluorescent image of brain slice shows ChR2-eYFP (green) expression in DMS from cortical projections and placement of carbon fiber electrode (CFE) used for fast-scan cyclic voltammetry to measure dopamine and glass pipette for electrical stimulation. Optogenetic stimulation was delivered via optic fiber (blue light). **c** Diagram of the striatal microcircuitry proposed: cholinergic interneurons (blue) are recruited by glutamatergic corticostriatal inputs (gray) to release acetylcholine (purple), which activates nAChRs on DA axons (green) to evoke local dopamine release. DHβE application blocks nAChRs to prevent DA release. **d**, **g**,** j**,** m** Left, ChR2-eYFP expression (green) in cortical injected area: anterior cingulate cortex (**d**), prelimbic cortex (**g**), visual cortex (VIS, **j**), auditory cortex (AUD, **m**). Right, ChR2-eYFP expressing projections (green) from the respective cortical areas in the dorsomedial striatum. The white box shows the site of dopamine recordings. Scale bars are 1 mm. **e**,** h**,** k**,** n** Single-slice average of dopamine transients measured with fast-scanning cyclic voltammetry (FSCV) and evoked by either optogenetic stimulation of corticostriatal projections (green) or electrical stimulation (gray) in the same recording site. Dashed lines show the remaining dopamine responses after washing in a nicotinic receptor blocker (1 μM DHβE). Traces are mean, and shaded areas are ± SEM). **f**,** i**,** l**,** o** Average amplitudes of dopamine transient in DMS elicited by electrical stimulation (gray error bars) and optogenetic stimulation (green error bars) before and after DHβE bath application (pink shade) for animals expressing ChR2-eYFP in ACA (**f**), PL (**i**), VIS (**l**,), and AUDp (**o**) cortical regions. Dots represent data from individual slices. Lines and shaded error bars are median ± bootstrap 95% CI. (ACA opto-evoked ACSF (*n* = 9 slices / 3 animals) vs. DHBE (*n* = 6/3), *p *= 0.001; ACA electrical ACSF (*n* = 9/3) vs DHBE (*n* = 6/3), *p* = 0.002; PL opto-evoked ACSF (10/4) vs DHBE (*n* = 5/3), *p* = 0.0007, PL electrical ACSF (*n* = 10/4) vs DHBE (*n* = 5/3), *p* = 0.003; Not significant: V1 opto-evoked ACSF (*n* = 19/8) vs DHBE (*n* = 4/2), V1 electrical ACSF (*n* = 20/8) vs DHBE (*n* = 4/2), A1 opto-evoked ACSF (*n* = 19/4) vs DHBE (*n* = 4/2), A1 electrical ACSF (19/4) vs DHBE (*n* = 4/2); two-sided Mann-Whitney U test.

Our results in Fig. 1 provide in vivo evidence that the visual stimulus-evoked dopamine responses depend on acetylcholine. Therefore, we next sought to determine whether the cortical inputs identified in Fig. 3 and tested functionally in Fig. 4 were capable of driving striatal dopamine release via an acetylcholine-dependent mechanism. We expressed an excitatory opsin (either ChR2 or ChrimsonR) in various cortical areas of C57B6/J mice (Fig. 5a;“Methods”) and used ex vivo fast scanning-cyclic voltammetry to measure dopamine transients elicited by optogenetic stimulations (470 nm, 5 ms, 1.3 mW; Fig. 5b, c) versus short electrical pulses (0.2 ms, 400 uA), that served as positive control. Optogenetic stimulation of ACA terminals consistently produced dopamine transients in DMS, although electrical evoked dopamine transients in this area were slightly larger, likely due to the additional signal from direct stimulation of dopamine axons (Fig. 5d–f; optogenetic: mean 0.39 [0.29 − 0.49 bootstrap 95% CI] μM, *n* = 9 slices from 3 animals; electrical: 1.43 [1.00 − 1.47] μM, *n* = 9 slices from 3 animals). Stimulating PL corticostriatal terminals produced similar results (Fig. 5g–i; optogenetic: 0.49 [0.39 − 0.56] μM, *n* = 10 slices from 4 animals; electrical: 0.79 [0.67 − 1.03] μM, *n* = 7 slices from 3 animals). To confirm that the cortically-evoked dopamine signal depended on CIN recruitment, we washed on a high concentration of DHβE (1 μM), a nicotinic acetylcholine receptor antagonist. Application of DHβE completely abolished any optogenetic evoked dopamine transients and significantly reduced electrical evoked events (Fig. 5f; ACA optogenetic DHβE: 0.011 [0.0096 − 0.025] μM, *n* = 6 slices from 3 animals; ACA electrical DHβE: 0.65 [0.42 − 0.86] μM, *n* = 6 slices from 3 animals; ACA optogenetic ACSF vs DHβE, *p *= 0.001; electrical ACSF vs DHβE, *p* = 0.007, Mann Whitney U test; Fig. 5i; PL optogenetic DHβE: 0.026 [0.0064 − 0.063] μM, *n* = 5 slices from 3 animals; PL electrical DHβE: 0.45 [0.27 − 0.62] μM, *n* = 5 slices from 3 animals; PL optogenetic ACSF vs DHβE, *p *= 0.0007; PL electrical ACSF vs DHβE, *p* = 0.02, Mann Whitney U test). In contrast, we found that optogenetic stimulation of sensory (VISp, or AUDp) corticostriatal terminals failed to produce any measurable dopamine transients in DMS (Fig. 5j–l, VISp: 0.015 [0.013 − 0.30] μM, *n* = 19 slices from 8 animals; Fig. 5m–o, AUDp: 0.011 [0.0098 − 0.054] μM, *n* = 19 slices from 4 animals). Interleaved electrical stimulation consistently produced dopamine transients in the same brain slices and under the same conditions, serving as positive controls and demonstrating feasibility (Fig. 5k, l, VISp: 0.67 [0.65 − 1.08] μM, *n* = 20 slices from 8 animals; Fig. 5n, o, AUDp: 0.33 [0.30 − 0.67] μM, *n* = 19 slices from 4 animals).

Together, these experiments provide ex vivo evidence that, unlike sensory cortices, frontal cortical areas such as ACA and PL produce dopamine release through a local mechanism requiring nAChRs, and further suggest that these select corticostriatal inputs can drive synchronized activation of CINs.

### Corticostriatal projections from frontal cortices respond to visual stimuli and can recruit CINs and drive striatal dopamine and acetylcholine in vivo

**Fig. 6:**
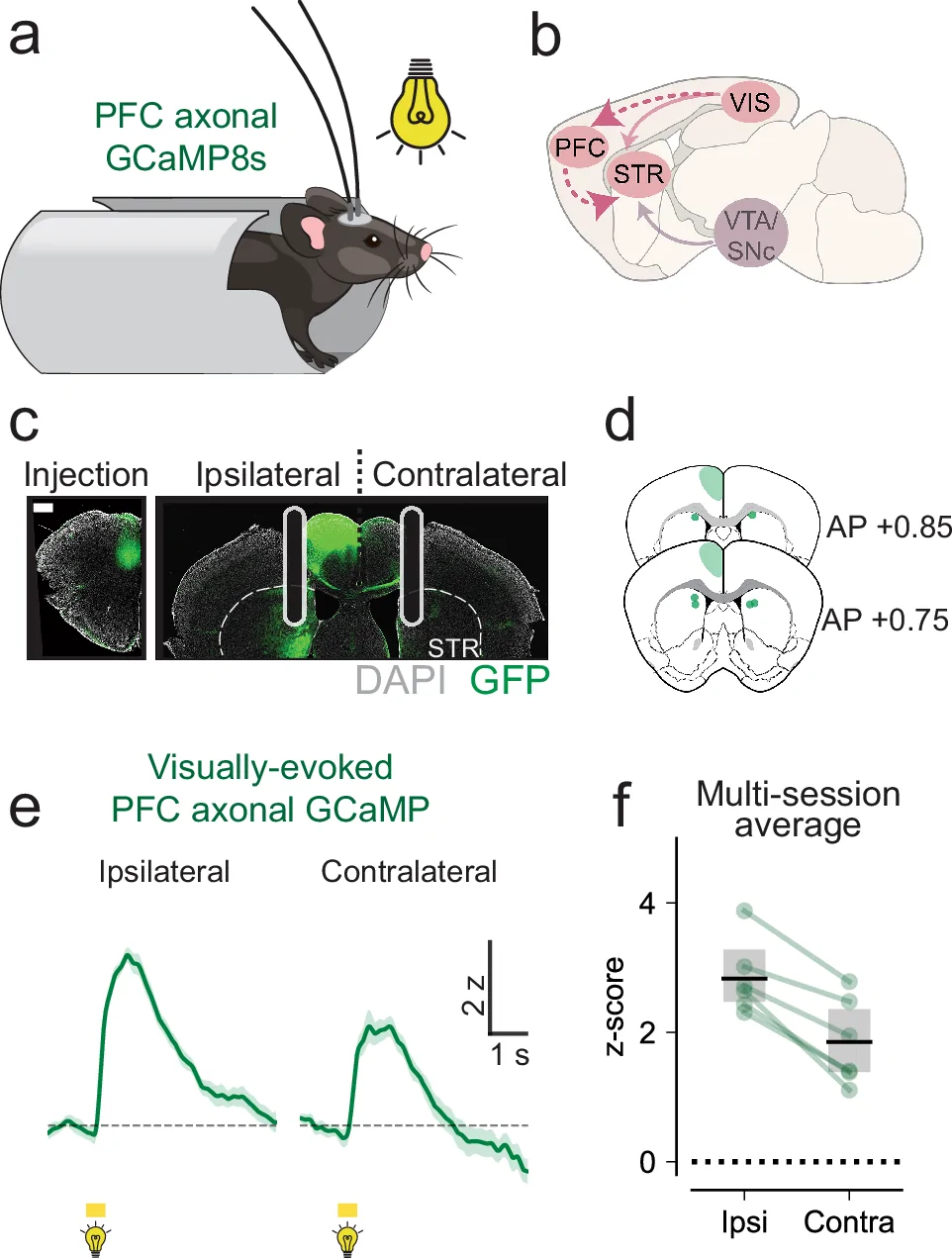
Visual stimuli produce time-locked activation of PFC corticostriatal projection. **a** GCaMP8s was expressed unilaterally in the prelimbic cortex and recording fibers implanted bilaterally in DMS. Animals were headfixed and presented with 500 ms visual stimuli, at least 6 weeks after surgery. **b** Diagram depicts a canonical pathway (purple) from VTA/SNc for nicotinic-independent striatal dopamine in DMS following visual stimuli. A putative additional pathway (pink) from PFC for the generation of nAChR-dependent striatal dopamine in DMS following visual stimuli. **c** Representative images of unilateral GCaMP8 expression in prelimbic cortex (right, injection site) and green fluorescent projections into DMS showing bilateral fibers (right). Scale bar is 500 µm. **d** Fiber placements in DMS for the animals used in the experiment. Fiber placements are displayed on Allen Brain Atlas plates^73^. **e** Representative average traces of visual-evoked calcium signals in PL axons in ipsilateral and contralateral DMS, relative to the injection site. Shaded regions are ± 95% bootstrapped CI. **f** Average z-score amplitude of PL axon calcium responses timelocked to light flashes recorded from the ipsilateral hemisphere and contralateral hemisphere relative to PL injection (*n* = 6 animals, lines represent the mean z-score and shaded error bars represent ± 95% bootstrapped CI).

We next returned to the in vivo preparation to ask whether PL/ACA axons projecting to DMS are activated in response to sensory stimuli (Fig. 6a, b). We expressed GCaMP8s in one hemisphere of the prelimbic cortex and recorded calcium signals bilaterally from the DMS (Fig. 6c, d). The ipsilateral hemisphere to the injection responded strongly to the light flash (Fig. 6e, f, left). The contralateral hemisphere, which receives fewer projections than the ipsilateral hemisphere, responded more weakly to the light flash (Fig. 6e, f, right).

**Fig. 7:**
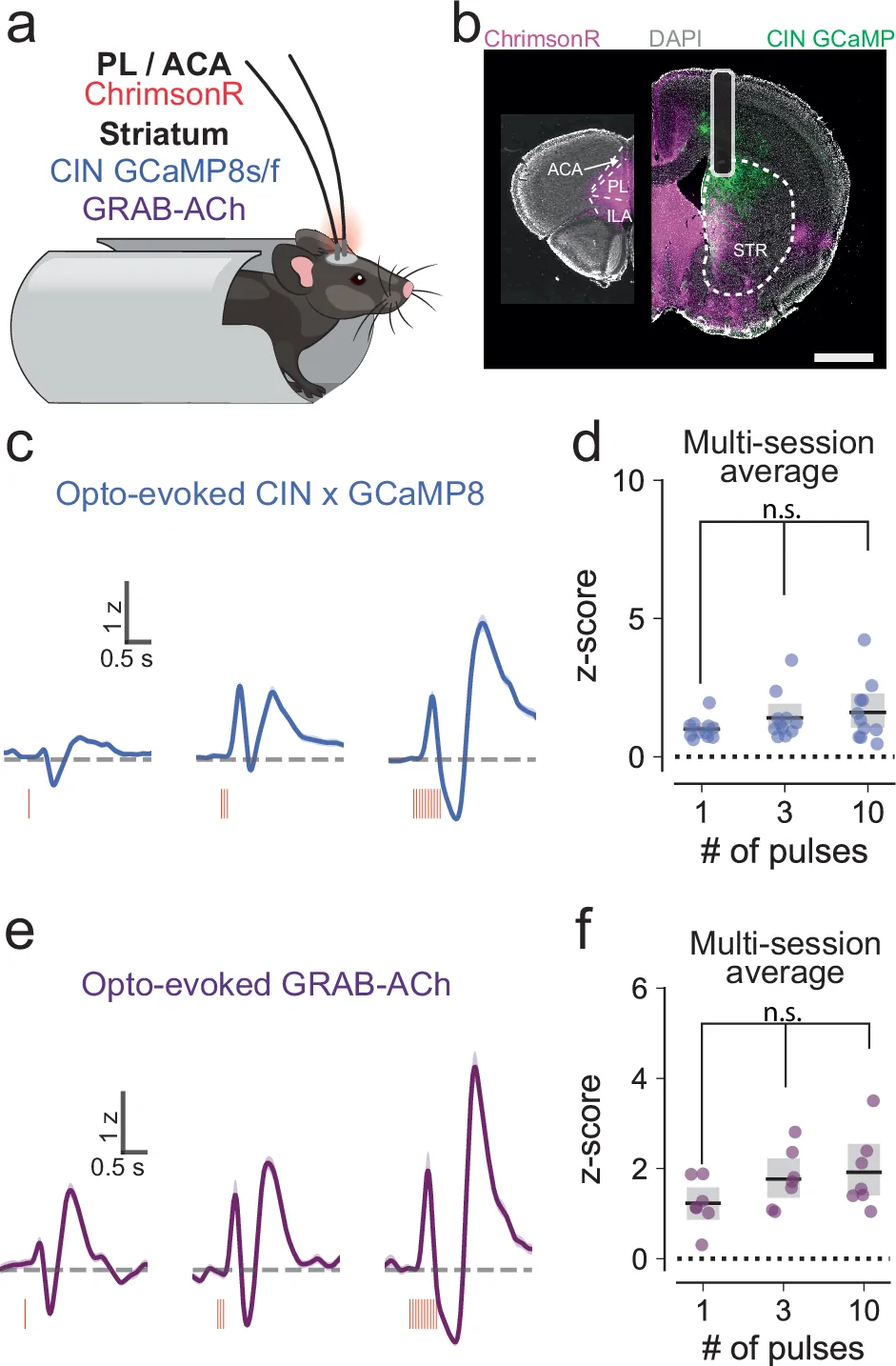
Corticostriatal projections from PFC recruit striatal CINs and drive acetylcholine release. **a** Head-fixed set up used for experiments for optogenetic stimulation of PFC corticostriatal projection in ChAT-IRES-Cre mice expressing GCaMP8 in CINs and wildtype mice expressing GRAB-ACh in DMS. **b** Example images of ChrimsonR-tdTomato expression in frontal cortical areas (pink) and their projection into dorsomedial striatum, also showing GRAB-ACh expression (green). Scale bar is 1 mm. **c**,** e** Representative traces of CIN GCaMP (**c**), and GRAB-ACh (**e**) responses to a single pulse (10 ms,625 nm), trains of three pulses at 20 Hz (center), or trains of 10 pulses at 20 Hz (right). **d**,** f** Maximum z-scored responses evoked by optogenetic stimulation of PFC projection in DMS for CIN GCaMP (**d**, *n* = 11 mice; *p* = 0.09, repeated measures one-way ANOVA) and GRAB-ACh (**f**, *n* = 7 mice; *p* = 0.08, repeated measures one-way ANOVA). Dots represent data from individual mice. Lines represent the mean, and gray error bars represent the bootstrapped 95% confidence interval.

Given that frontal corticostriatal projections carry visual information, we next sought to test whether in vivo optogenetic stimulation of PL/ACA terminals in DMS could recruit CINs and evoke acetylcholine signals, as seen with visual stimulation. ChAT-IRES-cre mice were injected bilaterally in PL/ACA with ChrimsonR-tdTomato and in the DMS with either a cre-dependent calcium sensor (DIO-GCaMP8s/f) or acetylcholine sensor (GRAB-ACh) in separate hemispheres (Fig. 7a, b). These mice were implanted bilaterally with fiber-optic cannulae, through which we could simultaneously record fluorescent changes and stimulate cortical terminals in DMS with short pulses of red light (625 nm, 5.5 mW; single pulse or trains of 3 and 10 pulses at 20 Hz, 30 s interval).

Stimulation of PL/ACA projections produced robust responses from both the calcium and acetylcholine sensors. These responses were also biphasic, consistent with the pattern seen in Fig. 2 and possibly aligned with the characteristic burst-pause-burst pattern of striatal CINs (Fig. 7c–f). As with sensory cues, the later phases of these multiphasic responses were heterogeneous (Supplementary Fig. S8). Therefore, for the quantitative analysis, we selected a time window corresponding to the first burst. A single pulse of PL/ACA terminal stimulation produced both CIN calcium responses (Fig. 7c, d; mean 1.0 [0.83 − 1.21 bootstrap 95% CI] z-score, *n* = 13 hemispheres from 11 animals) and acetylcholine responses (Fig. 6e, f; mean 1.23 [0.87 −1.59] z-score, *n* = 7 hemispheres from 7 animals). The amplitude of the first phase did not scale significantly with increasing stimulation (CIN GCaMP8s/f, 1 pulse: 1.00 [0.83 − 1.21] z-score; 3 pulses: 1.64 [1.12 − 2.41] z-score; 10 pulses: 1.84 [1.18 − 2.75] z-score, *p *= 0.09 one-way repeated measures ANOVA; GRAB-ACh, 1 pulse: 1.23 [0.86 − 1.58] z-score, 3 pulses: 1.77 [1.34 − 2.22] z-score; 10 pulses: 1.92 [1.40 − 2.53] z-score, *p* = 0.08 one-way repeated measures ANOVA Consistent with the proposed local circuit mechanism, neither sensor response decreased significantly after receiving a systemic injection of a nAChR blocker or vehicle (Supplementary Fig. 9).

**Fig. 8:**
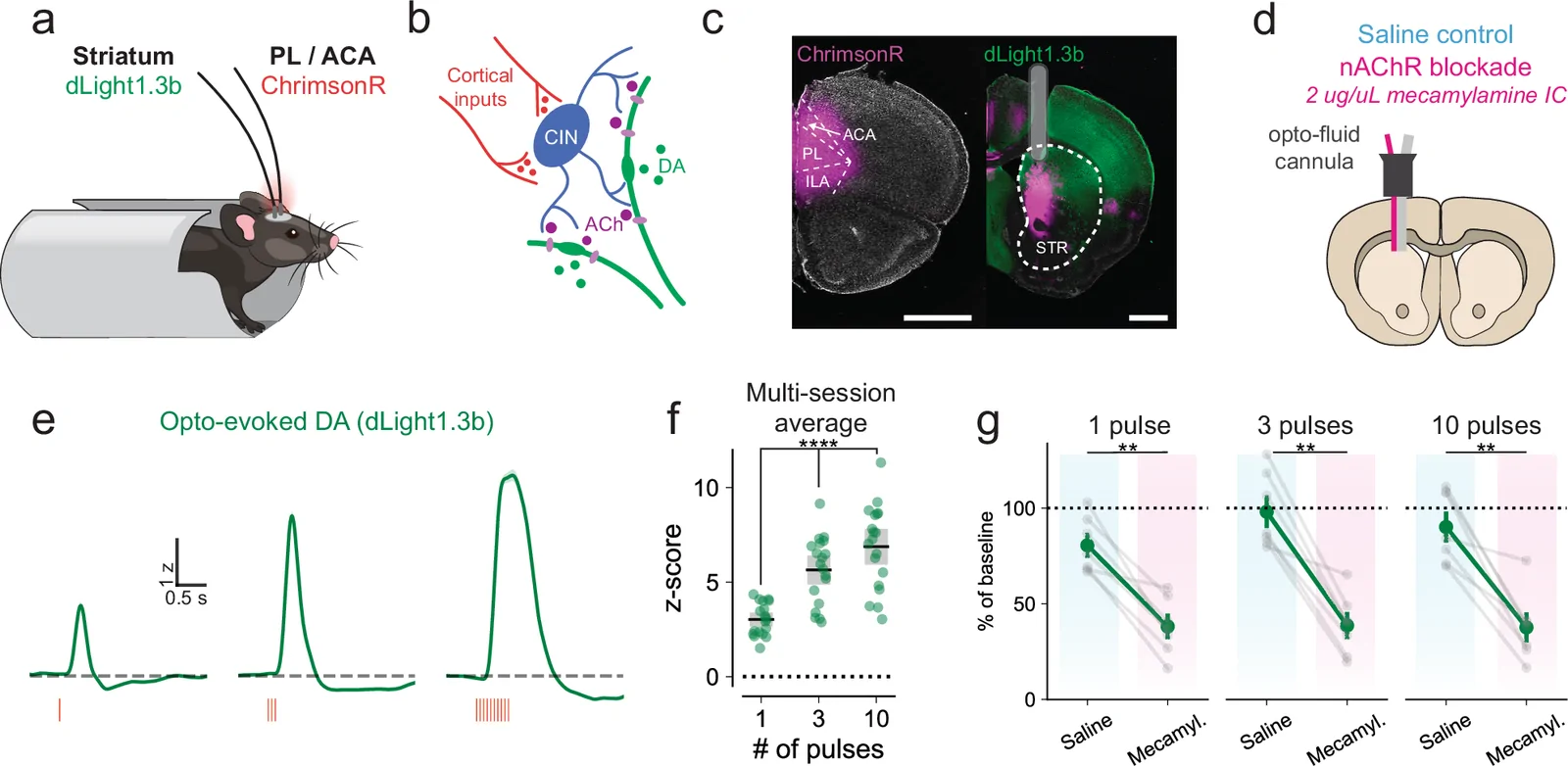
Stimulation of PFC corticostriatal projections triggers nAChR-dependent dopamine release in DMS. **a** Head-fixed experimental setup used for optogenetic stimulation of corticostriatal projections expressing Chrimson-tdTomato in the prelimbic or anterior cingulate cortex, bilaterally. Fiber implants in DMS bilaterally allow for optogenetic stimulation and recording of dLight, or GCaMP8 expressed in CINs, or GRAB-ACh expressed in the dorsomedial striatum. **b** Diagram of the proposed striatal microcircuitry: striatal cholinergic interneurons (blue) are recruited by glutamatergic inputs from corticostriatal ChrimsonR-expressing projections (orange) to release acetylcholine (purple), which activates nAChRs on DA axons (green) to produce local dopamine release. **c** Example images of ChrimsonR-tdTomato expression in frontal cortical areas (magenta) and their projection into the dorsomedial striatum, where dLight1.3b (green) was also expressed. **d** A subset of dLight-expressing mice were implanted with an optofluid cannula to allow intracranial infusions of saline or mecamylamine in DMS. **e** Representative traces of dLight1.3b responses evoked by optogenetic stimulation of PFC projections with a single pulse (10 ms, 625 nm) (left) and by trains of three pulses at 20 Hz (center) or trains of 10 pulses at 20 Hz (right). **f** Maximum z-scored dLight1.3b responses evoked by optogenetic stimulation of PFC projection in DMS (*n* = 11 mice; *p* < 0.0001, repeated measures one-way ANOVA). Green dots represent data from individual hemispheres. Lines represent the mean, and gray error bars represent the bootstrapped 95% confidence interval. **g** Effect of intracranial infusion of saline (blue) or mecamylamine (pink) on optogenetically-evoked dLight responses following single pulse, trains of 3 and 10 pulses stimulation. Amplitudes were normalized to each animal pre-infusion baseline. Error bars represent the mean ± sem. (*n* = 7 mice; treatment *p* = 0.0003, nPulses *p* = 0.04, interaction *p* = 0.15, repeated measures 2-way ANOVA).

Lastly, we asked whether stimulation of prefrontal corticostriatal terminals produced cholinergic-dependent dopamine release. Wildtype mice were injected bilaterally with ChrimsonR-tdTomato in the PL/ACA and dopamine sensor, dLight1.3b, in the DMS with bilateral fiber implants and headframes (Fig. 8a–c). A subset of these mice were also implanted unilaterally with an opto-fluid cannula to allow for intracranial delivery of mecamylamine during recordings (Fig. 8d). All mice received the same optogenetic stimulation protocol described above. Even a single 10-ms pulse of red light evoked measurable striatal dopamine responses (Fig. 8e, f, mean 3.02 2.95 − 3.75 bootstrap 95% CI] z-score, *n* = 19 animals, averaged across sessions). The amplitude of the dopamine response increased as the number of pulses increased, with evidence of saturation and prolonged duration with the longest stimulation train (Figs. 6e, f, 3 pulses: 5.65 [5.34 − 7.07] z-score; 10 pulses: 6.88 [6.45 − 8.75] z-score, averaged across sessions; *p* < 0.0001 one-way repeated measures ANOVA). We repeated these experiments in DLS with stimulation of SSp terminals and observed that stimulation produced small responses in CIN GCaMP8s/f and GRAB-ACh, but was unable to evoke positive dLight responses (Supplementary Fig. 10).

To test whether a component of this optogenetic evoked response was dependent on acetylcholine, a subset of mice received intracranial infusions of mecamylamine at the recording site (2 μg/μL at a rate of 0.06 μL/min). Blocking nAChRs produced significant decreases in dopamine at all stimulation levels (Fig. 8g; single pulse reduced to 38% ± 5.6%, 3 pulses reduced to 39% ± 5.9%, 10 pulses reduced to 38% ± 6.7%; treatment *p* = 0.0003, stimulation intensity *p* = 0.04, interaction *p* = 0.15, two-way repeated-measures ANOVA). Whereas only some mice received intracranial infusions, all mice received systemic intraperitoneal injections of 10 mg/kg mecamylamine (Supplementary Fig. 9). Systemic mecamylamine was not effective at suppressing the optogenetic-evoked dopamine (0% suppression vs 62% suppression with intracranial), unlike with visually-evoked dopamine responses. This may be due to a combination of factors: (A) the stronger over-drive of the projections achieved with optogenetic stimulation, and/or (B) the lower effective dose of blocker achieved by systemic administration, capped by peripheral effects.

Altogether, these in vivo experiments offer compelling evidence that brief activation of PL/ACA projections to the dorsomedial striatum can evoke cholinergic-dependent dopamine signals, via the local recruitment of CINs and subsequent ACh release.

## Discussion

This study provides new insights into how salient sensory stimuli control dopamine transmission in the striatum. Using a combination of anatomical and functional experiments, both in vivo and ex vivo, we identified which corticostriatal inputs can modulate striatal dopamine, and which cannot. We show that visual stimuli elicit striatal dopamine release in part through a cholinergic-dependent mechanism involving recruitment of CINs and acetylcholine release in the dorsomedial striatum. Despite the presence of prominent projections from the primary visual and auditory cortices to the dorsomedial striatum, these cortical areas do not connect strongly to striatal CINs and are unable to drive dopamine release. In contrast, frontal cortical areas such as the prelimbic and anterior cingulate cortices robustly recruit striatal CINs and acetylcholine to drive dopamine release ex vivo and in vivo. In mice, studies have demonstrated that the medial frontal cortex is increasingly activated by visual events throughout learning, and that inactivating this area dramatically affects an animal’s ability to perform visual tasks^45–47^. Thus, the current study identifies a key distinction between primary sensory and frontal cortical inputs to the striatum, with important implications for how the brain processes salient stimuli that support associative and reinforcement learning.

Previous studies showed that certain cortical and thalamic inputs can evoke striatal dopamine ex vivo, but two key questions remained: Which cortical areas selectively recruit CINs to regulate dopamine? Does this mechanism operate in vivo under behaviorally relevant conditions? Slice experiments demonstrated that inputs from the motor cortex and parafascicular thalamus strongly engaged CINs in the dorsal striatum and triggered dopamine-dependent plasticity^18^. The retrograde monosynaptic rabies tracing from our study supports these findings and identifies the dorsolateral and dorsocentral striatum as the target of the motor cortex and parafascicular thalamus inputs to CINs. Subsequent studies extended the findings to prelimbic cortex and intralaminar thalamus inputs^20,34^; and again, our anatomical and functional experiments here confirm those findings and further identify the dorsomedial subregion as the target from these PL/ACA inputs to CINs. In vivo stimulation of the prelimbic cortex was also shown to increase striatal glutamate, acetylcholine, and dopamine using microdialysis^20^, though with insufficient temporal precision to link dopamine release to behavioral events. Our results here address this gap by providing in vivo evidence of frontal cortex (PL/ACA) control over striatal dopamine with higher temporal precision. Crucially, we further demonstrate the ability of these corticostriatal inputs to recruit CINs and drive dopamine via nicotinic-dependent mechanisms. Functional and anatomical connectivity to CINs is restricted to specific cortical (frontal and motor) and thalamic inputs, and we show that it is absent in primary sensory cortices.

It is important to mention that in vivo optogenetic stimulation of cortical axons in the striatum is likely to also activate passing axons and axon collaterals that synapse onto other targeted areas. More specifically, PL/ACA projections to the dorsomedial striatum also send collaterals to dopaminergic midbrain regions, which likely contribute to a portion of the striatal dopamine signals we measured in vivo^48^. Also note that a recent study showed muscarinic receptors contribute to cholinergic regulation of dopamine in vivo, which could be responsible for the portion of the optogenetic response that remains after infusing mecamylamine (a nicotinic acetylcholine receptor antagonist)^49^. Thus, while not acting alone, a number of our experiments provide evidence for a local mechanism dependent on CINs and acetylcholine. First, intracranial local infusions of nAChR blocker mecamylamine successfully reduced dopamine responses evoked by both cue- and optogenetic stimulation of PL corticostriatal projections. Systemic mecamylamine dramatically reduced cue responses, but only slightly reduced the optogenetic-evoked dopamine responses, perhaps because the dose limitation for systemic administration was more easily overcome by the optogenetic over-stimulation of axons. Second, visual stimuli and ACA/PL projections also recruit CIN and drive acetylcholine release in vivo, as expected for local cholinergic-dependent dopamine release. Third, our ex vivo experiments isolated the local striatal microcircuitry and confirmed that corticostriatal projections from PL/ACA form strong functional synapses onto CINs and robustly recruit them to evoke local dopamine release, consistent with a direct cortical effect within the striatum. Because all axons from neurons projecting to the striatum are severed in ex vivo brain slice preparations, only the axonal projections within the slice are activated during the experiments. Any direct projections from the cortex to the midbrain dopamine neurons were excluded and cannot be responsible for the dopamine signals measured ex vivo. A last caveat to consider is that the acetylcholine release in the slices may arise from extra-striatal sources of acetylcholine axons, rather than striatal CINs. However, other studies have found that projections from the pedunculopontine nucleus or laterodorsal tegmentum do not produce dopamine signals in the striatum^50^. Altogether, we conclude that the unique ability of PL/ACA to evoke dopamine likely arises from the strength of its connections with CINs, a conclusion supported by both anatomical and functional data.

A strength of this study is its integration of dopamine, calcium, and acetylcholine measurements. However, direct comparison of signal magnitude and timing across these sensors is difficult because each differs in affinity and kinetics. At present, we can only interpret these timing differences cautiously. Importantly, a recent study examined the temporal relationship between acetylcholine and dopamine in vivo using precisely timed optogenetic stimulation of CINs combined with ultrafast dopamine imaging and identified a biphasic dopamine response^49^. The initial dopamine peak depended on β2-containing nicotinic receptors, which are high-affinity receptors expected to respond to even small increases in acetylcholine. Together, these findings support the idea that relatively small increases in acetylcholine may be sufficient to evoke dopamine release, as observed with single-pulse stimulation.

In considering our anatomical tracing data, it is important to note that rabies tracing results varied depending on the striatal location of the starter cells. When we targeted the anterior dorsomedial striatum, we observed projections from PL/AC to CINs. When we targeted central or lateral portions of the anterior striatum, we observed projections to CINs from the motor cortex and some somatosensory cortical areas. Thus, overall, there was a parallel pattern of medial-to-lateral segregation of inputs to CINs. Our tracing data refine previous work by detailing the cortical and thalamic sources of input to CINs within specific striatal subregions using an improved ChAT-IRES-Cre mouse line without common events of spontaneous recombination^51^. Different patterns would be expected for starter cells in the rostral portion of the striatum, the tail, or the nucleus accumbens. Future studies comparing these subregions are needed to assess whether similar connectivity rules apply.

While rabies tracing identifies the anatomical distribution and laminar origin of inputs to CINs, it cannot quantify the functional efficacy of the connections. To assess the functional synaptic strength, we therefore used electrophysiology. Our whole-cell recordings showed that CINs receive rare and weak inputs from visual and barrel cortices, whereas neighboring MSNs receive reliable synapses. Nonetheless, it remains possible that coincident activation from multiple sensory areas could summate to recruit CINs and evoke dopamine release under some specific conditions. Broadly, our functional results aligned with the anatomical data: rabies tracing revealed that CINs in the dorsomedial striatum (DMS) receive dense input from frontal regions, particularly ACA, whereas CINs in the dorsolateral striatum (DLS) are more strongly innervated by motor and somatosensory cortices. This topographical organization parallels the connectivity of cortical inputs to striatal MSNs^51–53^, reinforcing the view that the DMS is engaged in cognition and goal-directed behavior^54,55^, while the DLS supports action selection and habits^3,56,57^.

Our results provide direct support for the idea that visual stimuli and cortical inputs to the striatum can evoke dopamine release through a local circuit involving CINs. We acknowledge that the relationship between dopamine and acetylcholine is complex and remains controversial. Two studies showed that striatal acetylcholine and dopamine signals fluctuate in an anti-correlated manner in vivo^35,36^. These observations were further supported by recent findings from ex vivo slice experiments showing acetylcholine may, in some contexts, inhibit midbrain-evoked dopamine^21^, which could explain the anticorrelation observed in the in vivo signals. One of these studies reported that intracranial infusions of DHβE, a nicotinic receptor blocker, did not affect spontaneous dopamine signals, nor reward-driven signals^35^, which midbrain neurons would be expected to drive. Meanwhile, inhibiting acetylcholine release from CINs, striatum-wide, had significant effects on behavior and behaviorally evoked dopamine signals^36^. However, more local inhibition of acetylcholine release in the ventrolateral striatum had no effect in the same contexts^36^. These studies were also performed in the dorsolateral striatum and central striatum. Our results provide evidence for a local CIN-dependent circuit mechanism specifically within the DMS, and additional efforts will be required to understand possible regional differences in striatal circuit mechanisms that may eventually settle this important controversy.

More recent work in the nucleus accumbens shows compelling evidence that the local microcircuitry regulating striatal dopamine signals plays a role during both effortful tasks^30^ and active avoidance behavior^58^. Additional work in zebra finches has shown that the premotor cortex drives cholinergic-evoked dopamine necessary for song learning^59^. These studies align well with the current findings from our study, in which these local acetylcholine-dependent dopamine signals may work together and summate with midbrain-originated dopamine signals. Understanding the temporal dynamics of this cholinergic-dependent dopamine signal and how it integrates with midbrain-evoked dopamine is an important next step. Also, a deeper understanding of how CIN-dependent dopamine release contributes to the dopamine tone in vivo may clarify the long-standing discrepancies between tone and phasic dopamine regulation and functional role.

Lastly, we highlight a need to further explore the role of this acetylcholine-mediated control over dopamine throughout learning, stimulus-reward associations and also reinforcement learning, which may account for the historic role of CIN firing pattern in cue-reward learning.

In summary, our study reveals that sensory stimuli and frontal cortical regions, including the prelimbic and anterior cingulate cortices, robustly engage CINs and elicit acetylcholine signals to trigger striatal dopamine release in the anterior dorsomedial striatum. Primary sensory cortices, while projecting to the same striatal subregion, do not recruit CINs nor share the ability to drive striatal dopamine. Thus, we reveal a key distinction between sensory and frontal cortical inputs, identifying the latter as privileged drivers of striatal dopamine that may support associative and reinforcement learning. Disentangling the mechanisms behind cholinergic control over striatal dopamine and associated learning could prove critical to understanding the root cause of disorders involving dysregulation of striatal dopamine.

## Methods

### Animals

All experimental procedures were approved by the NIH Institutional Animal Care and Use Committee (IACUC) and complied with Public Health Service policy on the humane care and use of laboratory animals. Both female and male mice were used for all experiments. Electrophysiology experiments used either a ChAT-IRES-Cre (Δneo) line^60^ (for CIN or MSN recordings, B6.129S-*Chat*^*tm1(cre)Lowl*^/MwarJ; RRID: IMSR_JAX:031661), a Pvalb-IRES-Cre line (for MSN recordings, B6.129P2-Pvalbtm1(cre)Arbr/J; RRID: IMSR_JAX:017320), or a SST-IRES-CRE line^61^ (for MSN recordings, B6J.Cg-Ssttm2.1(cre)Zjh/MwarJ; RRID: IMSR_JAX:028864) crossed with an Ai14 Cre-dependent tdTomato line^62^ (B6.Cg-*Gt(ROSA)26Sor*^*tm14(CAG-tdTomato)Hze*^/J; RRID: IMSR_JAX:007914). Fast-scanning cyclic voltammetry experiments used wild-type C57B6/J mice or the ChAT-IRES-Cre (Δneo) x Ai14 cross. Rabies experiments were done in the ChAT-IRES-Cre (Δneo) line. C57BL6/J, DAT-IRES-cre^63^ (B6.SJL-Slc6a3tm1.1(cre)Bkmn/J, RRID: IMSR_JAX:006660), ChAT-IRES-Cre (Δneo), pDyn-IRES-cre^64^ (B6;129S-Pdyntm1.1(cre)Mjkr/LowlJ, RRID:IMSR_JAX:027958), and Drd1a-Cre (B6.FVB(Cg)-Tg(Drd1-cre)EY262Gsat/Mmucd, RRID: MMRRC_030989-UCD) mice were used for in vivo fiber photometry experiments.

### Fiber photometry

#### Surgeries

Animals (*N* = 63, 41 males, 22 females; age: 3 − 9 months, average 5 months, Table S1) were injected with AAV5-hSyn-dLight1.2^65^ (titer = 2.3E + 13; Addgene #111068-AAV5; RRID:Addgene_111068), AAV9-hSyn-dLight1.3b^65^ (titer = 2.5E + 13; Addgene #135762-AAV9; RRID:Addgene_135762), AAV9-Syn-FLEX-jGCaMP8s-WPRE^66^ (titer = 2.7E + 13, Addgene #162377-AAV9; RRID: Addgene_162377), AAV9-Syn-FLEX-jGCaMP8f-WPRE^66^ (titer = 2.0E + 13, Addgene #162379-AAV9, RRID: Addgene_162379) and/or AAV9-Syn-GRAB-ACh3.0(4.3)^67^ (titer  = 6.7E + 12, WZ Bioscience #YL001003-AV9-PUB) in DMS (400–500 nL at 180 nL / min; + 0.9 mm AP, ± 1.3 mm ML, − 2.8 mm DV from Bregma). Mice used for midbrain dopamine cell body recordings were injected with AAV9-Syn-FLEX-jGCaMP8s-WPRE in the VTA / SN (500 nL at 180 nL / mine; − 3.3 mm AP, − 0.6 mm ML, − 4.5 mm DV). A subset of these mice were additionally injected with AAV5-hSyn-ChrimsonR-tdTomato^68^ (titer = 1.20E + 13; Addgene #59171-AAV5; RRID:Addgene_59171) in prelimbic cortex (120 nL at 120 nL / min; + 2.1 mm AP, ±0.4 mm ML, − 2.3 mm DV from Bregma) or the anterior cingulate cortex (120 nL at 120 nL / min; + 1.0 AP, ± 0.3 mm ML, − 1.5 mm DV from Bregma). A custom headpost and optic fiber cannulae (DMS: 400um Ø, 0.37 NA, 2.5 mm long; VTA/SN: 200um Ø, 0.37 NA, 4.5 mm long; Doric) were implanted above the injection sites using Metabond (C&B Parkell).

#### Photometry system

Thorlabs LED drivers (LEDD1B) were used to drive Thorlabs 405 nm (M405F1), 470 nm (M470F4), and 625 nm (M625F2) fiber-coupled LEDs connected to a six-channel Doric filter cube (FMC6_IE(400-410)_E1(460-490)_F1(500-540)_E2(555-570)_F2(580-680)_S) by 0.5 NA / 400 μm Ø patch cords (Thorlabs, M301L01). Subject cords were custom-ordered from Doric (MFP_400/440/1100-0.37_1m_FCM-ZF1.25(FP)_LAF). Newport Femtowatt Receivers (Doric, NPM_2151_FOA_FC) were connected to the filter cubes using custom 0.5 NA / 600 μm Ø patch cords (Thorlabs). Femtowatt receivers fed into a Tucker Davis Technologies RZ5P processor. All fiber optic cords were photobleached for 24 h before the start of the experiment. 405 nm and 470 nm LEDs were driven at 215 and 326 Hz. Data were collected at 1017 Hz and demodulated using Tucker Davis Technologies Synapse software. An Arduino interfaced with the 625 nm LEDs and the RZ5P processor controlled and timestamped optical stimulation.

#### Sensory and optogenetic stimulation

405 nm and 470 nm LEDs were calibrated daily to 20 μW and 30 μW, respectively, as measured at the fiber tip. Mice were head-fixed in an acrylic tube for the duration of recordings. Mice were headfixed into a custom head fixation setup with their body resting within an acrylic tube. All behavioral tasks were performed in a dark, sound-attenuated behavior chamber (Med Associates) fitted with 20 mm black sound-insulating foam. All behavioral tasks and external stimuli were controlled using pyControl^69^ (Open Ephys Production Site). Mice only experienced each stimulus five times per session, with a maximum of two sessions per day, to avoid habituation.

Auditory cues were delivered at 7.5 KHz at 70 dB for 500 ms (a volume level comparable to normal conversation). Tones were delivered via an 8 ohm speaker (DigiKey; part number GF0401M-ND) placed 6 inches directly above the mouse’s head. The speaker was connected to a microcontroller (ATmega328P) where tones were generated using Arduino’s tone function. Visual cues were delivered via a pyControl houselight LED that uses three 16 lux LEDs on a plastic strip, placed in the behavior chamber above / slightly in front of the mouse. The luminance of the LED was measured to be 5.9 cd/m^2^ on the floor of the box, and 0.6 cd/m^2^ on the black foam sidewalls (light intensity comparable to a nightlight or natural light at dawn / dusk). The visual stimulus was intentionally dim to avoid a startle response that could be produced by a brighter light in contrast to the dark behavior box.

Optogenetic stimulation used 625 nm LEDs calibrated to 5.5 mW output. Optogenetic stimulation was delivered every 30 seconds in either single 10 ms pulses, 10 ms three-pulse trains at 20 Hz, or 10 ms ten-pulse trains at 20 Hz.

#### Pharmacology

Mecamylamine (2843/10, Tocris) was prepared fresh in 0.9% sterile saline and filtered for a final concentration of 2 ug / uL for intracranial infusion or 10 mg / 10 mL for intraperitoneal injection, allowing mice to be injected with a 10 mL / kg dosage. During pharmacology sessions, 7.5 min of optogenetic data were collected followed by 5 repetitions of each cue. For intracranial infusions, a SP101l syringe pump (world precision instruments) loaded with a 1700 series 10 μL gas-tight syringe (Hamilton, 80008) was used to start the infusion (mecamylamine or saline vehicle) at a rate of 0.06 μL / min and allowed to flow for ~ 10 minutes before the optogenetic stimulation and cue protocols were repeated. For intraperitoneal injections, mice were injected intraperitoneally with mecamylamine (10 mg / kg prepared for a final 10 mL / kg volume) or saline (10 mL / kg) and returned to their home cage for 30 minutes before repeating the optogenetic stimulation and sensory cue protocols.

#### Analysis

Data were converted to ΔF/F signals using GuPPy, an open-source Python package^70^ (RRID: SCR_02235345). ΔF/F was calculated by subtracting the isosbestic channel from the signal channel and dividing by the isosbestic. The same Python package was used to baseline-correct the traces and create PSTHs. The preprocessed data were then further analyzed and plotted in Python. Data were z-scored either over a single file (baseline non–pharmacological sessions) or over the “pre” and “post” files together (pharmacological sessions) by calculating the mean and standard deviation of the trace(s), subtracting the mean and dividing by the standard deviation. Baseline data were averaged per mouse across sessions. Pharmacological data were always collected and reported from a single session.

Peak amplitude of the evoked responses were calculated by finding the maximum value within 2 s from stimulation for dLight (which produced larger, longer responses with a single peak) or within a 0.6 s window from stimulation for GCaMP or GRAB-ACh (which tended to produce multiphasic responses with faster phases). Because later phases of the responses were heterogeneous, we limited the analysis to the first phase of the response, which was consistent across animals.

#### Histology

Mice were deeply anesthetized with isoflurane and transcardially perfused with phosphate-buffered saline, followed by 4% paraformaldehyde (PFA). Brains were extracted, post-fixed in 4% PFA at 4 °C overnight, and transferred to 30% sucrose in PBS for > 48 h at 4 °C. Brains were flash frozen in isopentane and stored at − 80 °C. 35 µm coronal cryo-sections were cut using a cryostat and collected in PBS containing 0.01% sodium azide. To verify viral expression and fiber placement, brain sections were gently rocked for 3 × 10 min in PBS, blocked with 10% normal goat serum (NGS; Jackson Immuno, 005-000-121; RRID: AB_2336990) in PBS containing 0.1% Triton X-100 (PBS-Tx) for 1 h at room temperature, and then incubated in rabbit anti-GFP, 1:500 (Invitrogen, G10362; RRID: AB_2536526) primary antibody in PBS-Tx at 4 °C for 24 h. Sections were rinsed 3 × 10 min with PBS and incubated in 488 anti-rabbit,1:800 (Jackson Immuno, 111-545-003; RRID: AB_2338046) secondary antibody in PBS-Tx for 2 h at room temperature. Sections were washed 3 × 10 min with PBS, mounted on slides with coverslip and ProLong™ Gold antifade reagent with DAPI (Invitrogen, P36935). All slides were imaged using consistent exposure settings on a ZEISS Axioscan 7 slide scanner or a Keyence BZ-X810 microscope.

### Slice physiology

#### Surgeries

Animals (*n* = 47; 21 males, 26 females; age: 2-13 months, median 3 months) were injected with AAV5-hSyn-ChR2(H134R)-eYFP (titer = 1.80E + 13; Addgene, #26973; RRID:Addgene_26973) and/or AAV5-hSyn-ChrimsonR-tdTomato (as above). PL injections (100-120 nL at 120nL/min) were targeted to + 2.1 mm AP, ± 0.4 mm ML, − 2.3 mm DV from Bregma. ACA injections (100-120 nL at 120nL/min) were targeted to + 1.0 mm AP, ± 0.3 mm ML, − 1.5 mm DV from Bregma. Somatosensory cortex injections (120 nL at 120 nL/min) were targeted to − 1.25 mm AP, ± 3.0 mm ML from Bregma, and − 0.5 mm DV from Pia. Visual cortex injections (120 nL at 120 nL/min) were targeted to −3.5 mm AP, ± 2.3 ML from Bregma, and − 0.5 mm from Pia. Auditory cortex injections (120 nL at 120nL/min) were targeted to − 2.7 mm AP, ±  4.3 mm from Bregma, and − 0.7 mm from Pia.

#### Slice preparation

Mice were anesthetized and decapitated at least four weeks post-surgery. Brains were extracted, mounted on a vibratome (VT-1200S, Leica Microsystems), and sliced coronally (240 μm) in oxygenated cutting solution heated to 32 °C, containing the following (in mM): 90 sucrose, 80 NaCl, 24 NaHCO3, 1.25 NaH2PO4, 10 glucose, 3.5 KCl, 0.5 CaCl2, 4.5 MgCl2, and 3 kynurenic acid. Slices were incubated for 20 min at 32 °C in ACSF containing the following (in mM): 124 NaCl, 1 NaH2PO4, 2.5 KCl, 1.3 MgCl2, 2.5 CaCl2, 20 glucose, 26.2 NaHCO3, and 0.4 ascorbic acid, and kept at room temperature after that until use. The recording chamber was perfused at 2 ml/min with ACSF heated to 32 °C using an inline heater (Harvard Apparatus).

#### Fast scanning-cyclic voltammetry

FSCV was performed in the dorsal striatum. Carbon-fiber electrodes (CFEs) were prepared with a cylindrical carbon fiber (7 μm diameter, ∼ 150 μm of exposed fiber) inserted into a glass pipette. Before use, the CFEs were conditioned with an 8-ms-long triangular voltage ramp (− 0.4 to 1.2 and back to − 0.4 V vs Ag/AgCl reference at 400 V/s) delivered every 15 ms. CFEs showing current > 1 .8 µA or < 1 .0 µA in response to the voltage ramp at ∼ 0.6 V were discarded. CFEs were held at − 0.4 V versus Ag/AgCl, and the same triangular voltage ramp was delivered every 100 ms. Using the same CFE and location, dopamine signals were evoked by alternating electrical and optical stimulation, delivered every 2 mins. For electrical stimulation, a glass pipette filled with ACSF was placed near the tip of the carbon fiber and a rectangular pulse (0.2 ms, 400 μA). For optogenetic stimulation, 470 nm blue light (1.3 mW) was delivered through either a 40 x objective via a CoolLED (pE-800) or a fiber-optic patch cord (200 μm diameter, 0.22 NA, ThorLabs) attached to a ThorLabs fiber-coupled LED (M470F4, driven by an LEDD1B driver). The light source was placed over the CFE to deliver square light pulses (5–10 ms). Data were collected with a retrofit headstage (CB-7B/EC with 5 mΩ resistor) using a Multiclamp 700B amplifier (Molecular Devices) after a low-pass filter at 3 kHz and digitized at 100 kHz using a data-acquisition board (NI USB-6229 BNC, National Instruments). Data acquisition and pre-processing were performed using custom-written software, VIGOR, in Igor Pro (Wavemetrics) using mafPC (courtesy of MA Xu-Friedman). Further analysis was conducted using Python. The current peak amplitudes of the evoked dopamine transients were converted to dopamine concentration according to a post-experimental calibration using 1–3 μm DA.

#### Electrophysiology

Striatal CINs were identified by fluorescence and confirmed by their characteristic spontaneous firing pattern. Whole-cell recordings were performed from CINs in the striatum using glass pipette electrodes with a resistance of ∼ 3-4 MΩ, filled with an internal solution (pH 7.25, 290-310 mOsm) containing the following (in mM): 120 CsMS, 10 CsCl, 10 HEPES, 0.2 EGTA, 10 sodium phosphocreatine, 4 Na2-ATP, and 0.4 Na-GTP. For input-output curves, the internal solution also contained 4.4 mM lidocaine to block sodium channels and prevent firing. Cholinergic interneurons were held at a holding potential of − 70 mV to keep these spontaneously firing neurons far away from firing threshold. Medium spiny neurons were held at a holding potential of − 70 mV or − 55 mV, where the cell input resistance is higher. All recordings were done in the presence of 5 μM CPP (Tocris, #0247) to block NMDA currents. Excitatory post-synaptic currents were recorded in response to 1 or 10 ms duration square light pulses (titrated based on synapse strength to avoid inducing action potentials; 10 ms was used when there was no response, to demonstrate that even very intense stimulations could not produce a response). A dual-color LED (Doric LEDC2_405/ 595) was used to alternate between pulses of violet-shifted (405 nm, 1.6 mW) or orange light (595 nm, 1.0 mW). Data were collected using a Multiclamp 700B amplifier after a low-pass filter at 1 kHz and digitized at 5 kHz using pClamp10 software (Molecular Devices).

#### Analysis

Data were analyzed in Python using pyABF (https://github.com/swharden/pyABF). Sweeps were filtered with a 100 Hz 4th-order lowpass Butterworth filter to remove high-frequency noise. Data were baseline corrected to a standard window prior to stimulation, and noise was subtracted to the height of the average peak within that window. Peaks were detected as the minimum (EPSCs) within a 300 ms window from stimulation. For visualization, the absolute values of the EPSCs were plotted as positive numbers. All data are presented as the mean and SEM.

### Anatomical tracing

ChAT-IRES-Cre (Δneo) (*n* = 12, 7 males, 5 females; age: 2-21 months, median 2 months) were injected unilaterally in DMS or DLS, as described above, with helper viral vector AAV8-DIO-Ef1a-TVA-Flag-2A-N2cG (Addgene #172360-AAV8; RRID:Addgene_172360), and rabies retrograde vector EnVA-CVS-N2c-tdTomato-FlpO one week apart^71^. Six days after the second injection, mice were deeply anesthetized with isoflurane and transcardially perfused with phosphate-buffered saline, followed by 4% paraformaldehyde (PFA). Brains were removed, fixed overnight in 4% PFA and transferred to 20% sucrose in PBS. After the brains had equilibrated in 20% sucrose/PBS, coronal sections were cut at 50 μm on a freezing microtome and collected in PBS. TdTomato labeling in trans-synaptically rabies labeled neurons was amplified immunohistochemically using sequential incubation in primary antibodies, rabbit anti-RFP (Rockland, 600-401-379) followed by secondary antibodies goat anti-rabbit Alexa Fluor 555 (ThermoFisher, A32732). Sections were mounted onto slides and labeled with the fluorescent blue Nissl stain Neurotrace 435 (ThermoFisher, N21479). All sections through the brain from the frontal pole to the brainstem were mounted sequentially onto slides (96 sections/mouse) and imaged at 10x (Zeiss PlanNeoFluor objective, 10x NA0.3) using a Zeiss AxioImagerM2 fluorescence microscope with an OrcaFlash4.0 camera and Ludl motorized 2axis stage controlled by Neurolucida software (MBF Bioscience, Williston, VT). Each 50 μm section was imaged in 7 μm steps, and the tiled images were then compiled into a single image and collapsed into a single plan using the DeepFocus function in Neurolucida. Analysis of labeled neurons by reconstructing the series of serial sections into a whole brain volume, detecting and marking labeled neurons and registering the location of neurons labeled with tdTomato into the Allen Brain Atlas using NeuroInfo Software^72^. To display the brain wide distribution of cortical neurons providing synaptic inputs to striatal ChAT neurons coordinates of presynaptic rabies labeled cortical neurons registered to the Allen CCF are projected along curved streamlines of the cortex to a flattened mapping of the cerebral cortex^73^. Data were further analyzed in Python and are presented as a percentage of the total labeled cells in each overall region (cortex, thalamus, midbrain, and basal ganglia).

### Statistics and reproducibility

Sample sizes were chosen based on previous studies performed using the same experimental techniques. Data for each experimental condition were replicated in at least two independent cohorts of mice.

The experimental design included negative and positive control conditions within the same animal whenever possible, alongside the test condition, to minimize inter-animal variability and allow within-animal comparison across conditions. Animals were assigned to experimental cohorts according to the requirements of the study design. When possible, experiments were replicated using slightly different viruses (ChR2 vs Chrimson, GCaMP8s vs GCaMP8f), or genotypes (SST-cre, PV-cre, ChAT-cre), or injection areas (PL, ACC). Histological analyses and evaluation of fiber placement were performed by investigators blinded to experimental conditions. No data were excluded from the analyses unless predefined technical or histological criteria were not met, including inaccurate fiber placement, loss of tissue integrity preventing reliable histological assessment, or technical failure during data acquisition. All exclusions, when applicable, were determined before statistical analysis.

Statistical analyses were performed using appropriate tests based on the structure of the data and experimental design. For comparisons between two groups, a one-way ANOVA or a Mann-Whitney U was used, as appropriate. For comparisons involving more than two groups or conditions, a two-way ANOVA was used. When repeated measurements or within-animal comparisons were analyzed, statistical tests accounting for paired or repeated-measures designs were used. Data are generally presented as mean ± sem, as indicated in the figure legends. Statistical significance was defined as *P* < 0.05.

### Reporting summary

Further information on research design is available in the Nature Portfolio Reporting Summary linked to this article.

## Supplementary information

Supplementary Information

Reporting Summary

Transparent Peer Review file

## Source data

Source Data

## Data Availability

All processed data used in this study are available in the source file. Raw data will be made available upon request, to prevent duplication and saturating storage demand due to the large size. Source data are provided in this paper.

## Code availability

All code used to produce the figures and run statistical testing can be found on Zenodo^74^
