# Supplementary material for: Cholinergic-dependent dopamine signals in mouse dorsomedial striatum are regulated by frontal but not sensory cortices
Source: Nat Commun. 2026 Sep 2;17:9601. doi: 10.1038/s41467-026-77168-x (PMC13554099; doi:10.1038/s41467-026-77168-x)
Supplement: Supplementary file 1 [file 41467_2026_77168_MOESM1_ESM.pdf]

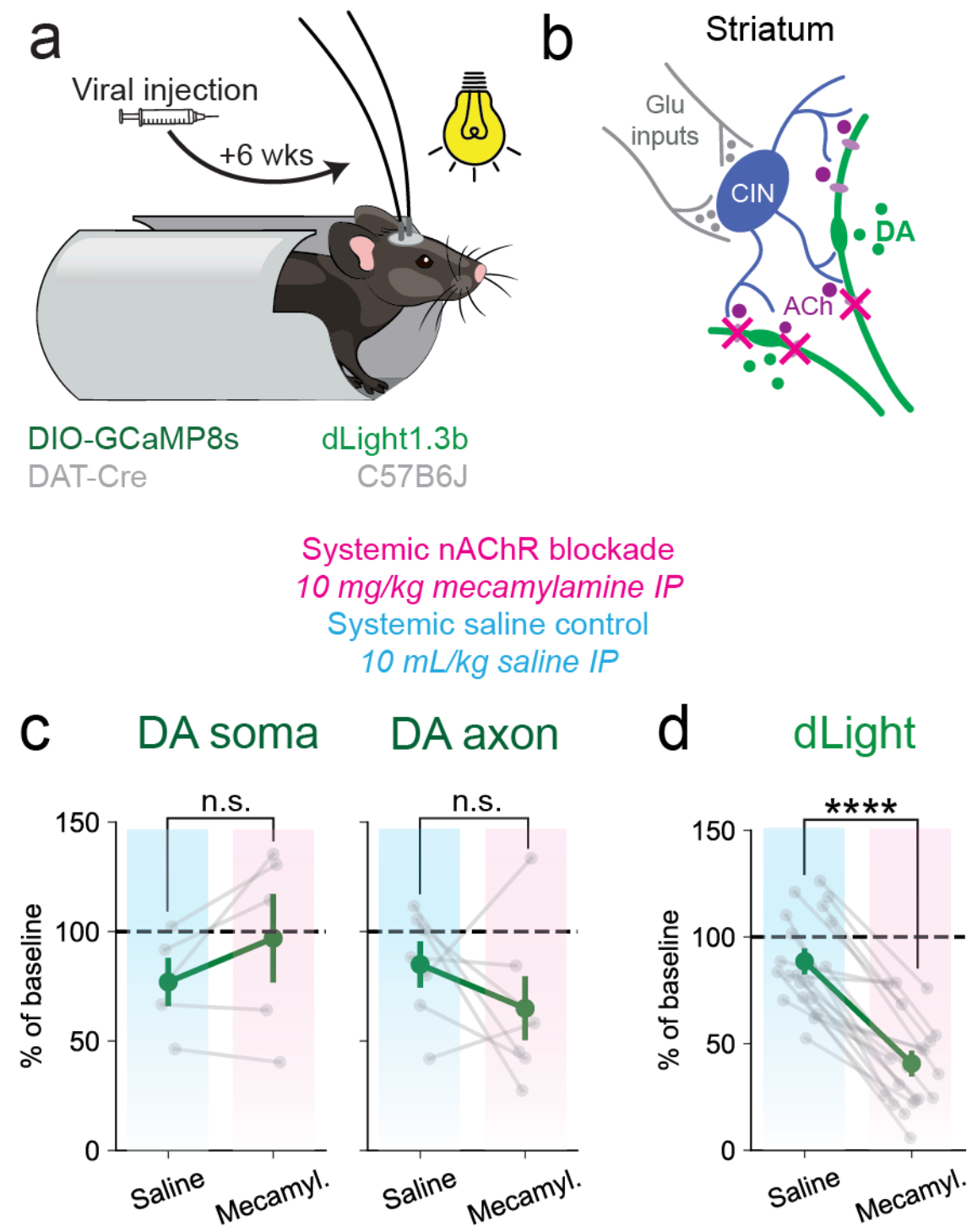

Supplemental Figure 1. Systemic mecamylamine suppresses striatal cue-evoked dopamine responses

a. Experimental setup. DAT-IRES-cre mice expressing DIO-GCaMP8s or wildtype mice expressing dLight1.3b in DMS were head-fixed and presented with white light (500 ms, 5.9 cd/m^2) and tones (7.5 kHz, 500 ms), spaced roughly every 30 seconds. DAT-GCaMP signals were recorded from both VTA/SNc (somas) and DMS (axons).

b. Diagram of proposed striatal microcircuitry: cholinergic interneurons (CIN, blue) are driven by glutamatergic inputs (gray), leading to release of acetylcholine (purple), which activates nAChRs on dopamine neuron axons (green) to produce local dopamine release.

c. Neither intraperitoneal saline injections nor mecamylamine injection affected DA somas (n = 5 animals) or axons (n = 7 animals). Saline reduced soma responses to 77% of baseline vs. a reduction to 97% by mecamylamine. Saline reduced axonal responses to 85% of baseline vs. a reduction to 65% by mecamylamine (treatment p = 0.26, recording location p = 0.42, interaction p = 0.45; two-way repeated measures ANOVA). Gray lines are individual animals and green lines represent the group mean ± sem.

d. Intraperitoneal mecamylamine injection significantly reduced dLight1.3 responses to the light stimulus. Saline reduced dLight responses to 89 ± 4.8% of baseline (mean ± sem). Mecamylamine reduced dLight responses to 41 ± 4.8% of baseline (n = 20 animals, treatment p < 0.0001, one-way repeated measures ANOVA). Gray lines are individual animals and green lines represent the group mean ± sem.

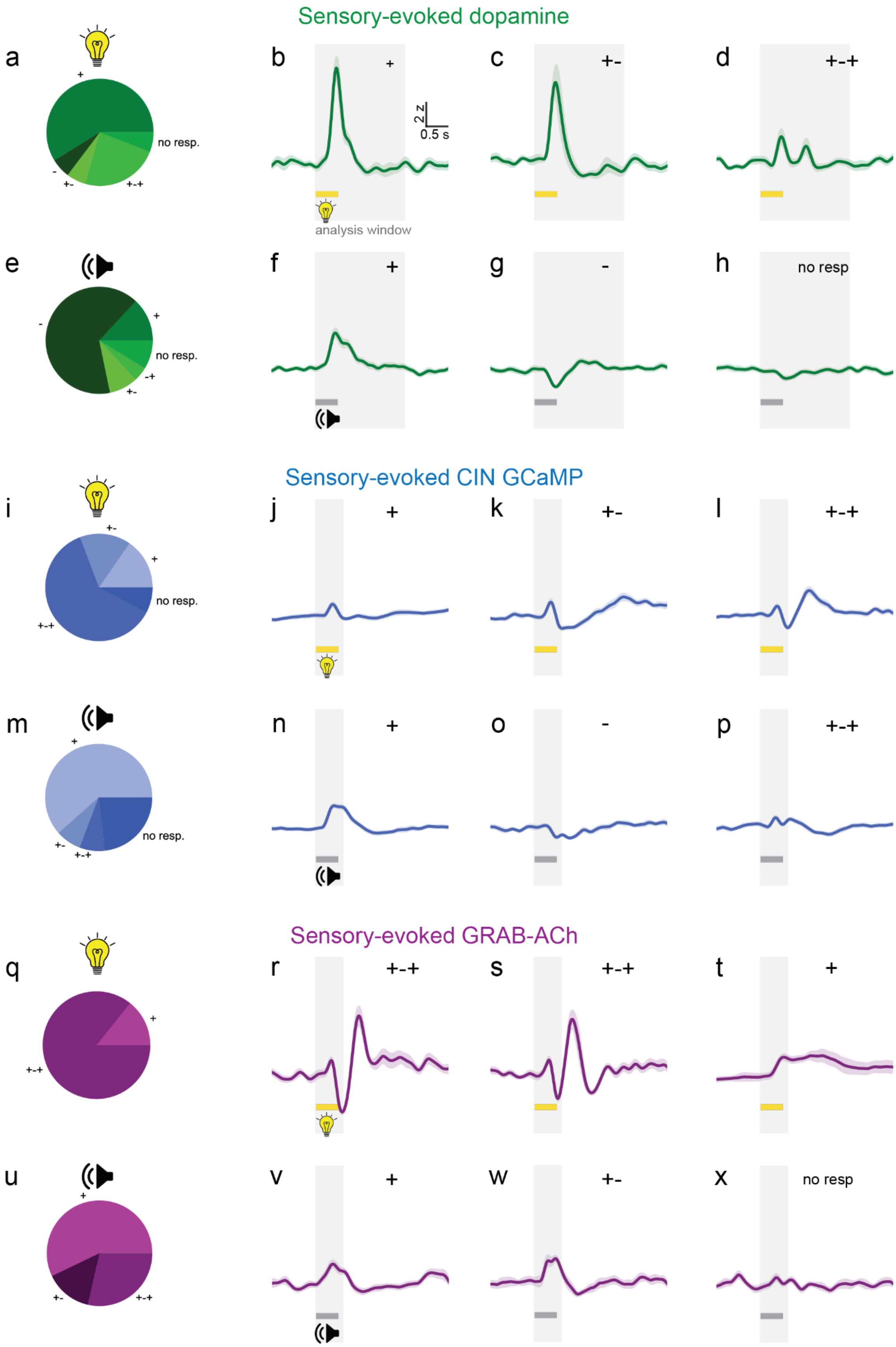
Sensory-evoked dopamine
a
+
no resp.
-
+-
+-+
b
+
2 z
0.5 s
analysis window
c
+-
d
+-+
e
+
-
no resp.
-+
+-
f
+
g
-
h
no resp
Sensory-evoked CIN GCaMP
i
+-
+
no resp.
+-+
j
+
k
+-
l
+-+
m
+
no resp.
+-
+-+
n
+
o
-
p
+-+
Sensory-evoked GRAB-ACh
q
+
+-+
r
+-+
s
+-+
t
+
u
+
+-
+-+
v
+
w
+-
x
no resp

Supplemental Figure 2. Heterogeneity in cue responses across mice

a, e. Percentages of mice that exhibited positive monophasic (+), inverted monophasic (-), biphasic (+-), inverted biphasic (-+), and triphasic dopamine responses to cue and tone flashes (+-+).

b-d, f-h. Example traces from three different mice, demonstrating heterogeneity in dopamine responses. Gray shaded regions indicate the time window used for initial peak-detection (2 s).

i, m. Percentages of mice that exhibited positive monophasic (+), biphasic (+-), and triphasic CIN GCaMP responses to cue and tone flashes.

j-k, n-p. Example traces from three different mice, demonstrating heterogeneity in CIN GCaMP responses. Gray shaded regions indicate the time window used for initial peak-detection (0.6 s).

q, u. Percentages of mice that exhibited positive monophasic (+), biphasic (+-), and triphasic GRAB-ACh responses to cue and tone flashes.

r-t, v-x. Example traces from three different mice, demonstrating heterogeneity in acetylcholine responses. Gray shaded regions indicate the time window used for initial peak-detection (0.6 s).

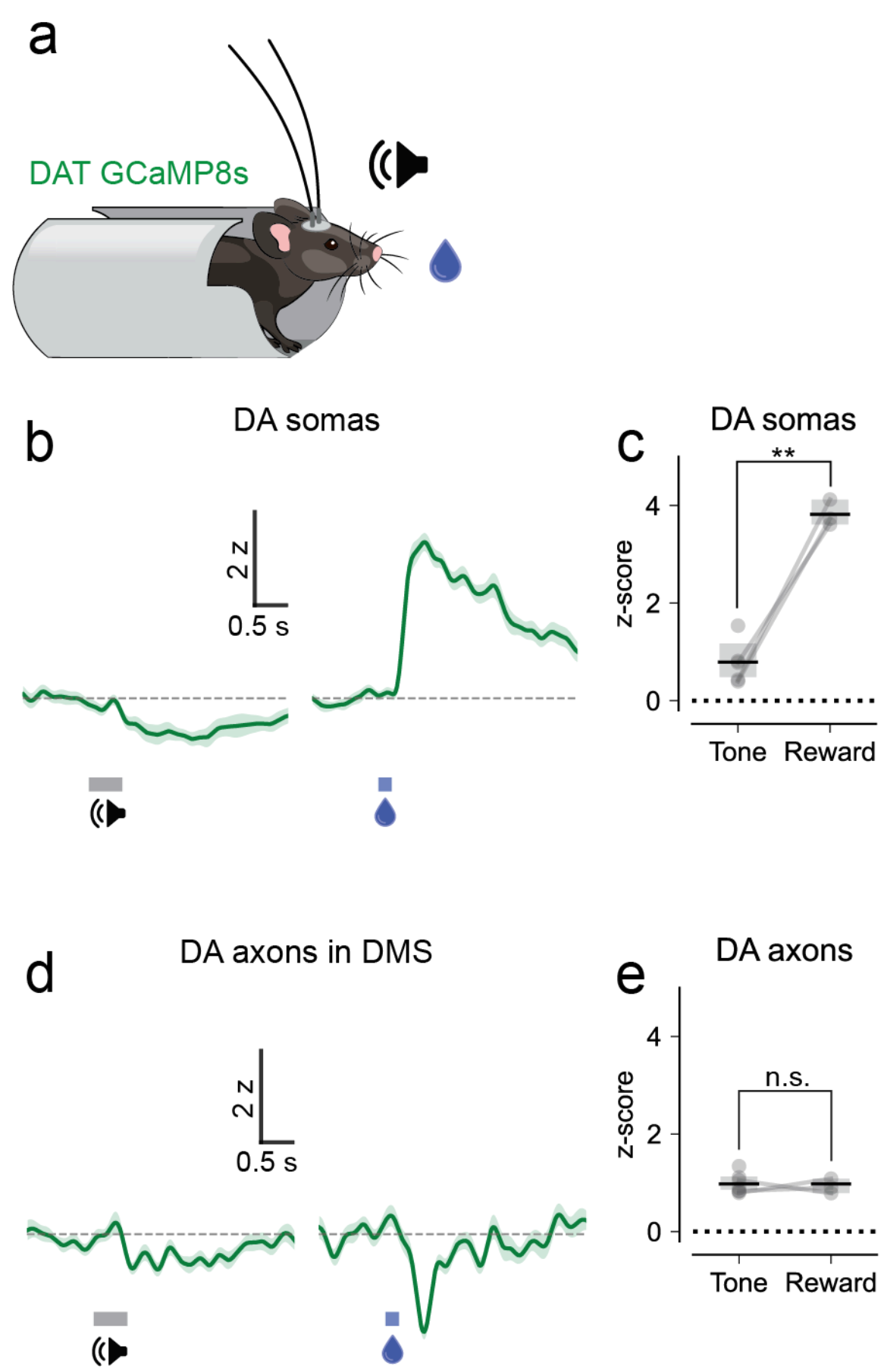

Supplemental Figure 3. Dopamine neurons respond more strongly to reward than to tone

a. Water-restricted DAT-IRES-cre mice expressing DIO-GCaMP8s were headfixed and presented with auditory tones and water-drop rewards while fluorescent calcium signals were recorded from midbrain and DMS.

b. Representative GCaMP8s fluorescence traces from DA somas in response to auditory tones and water reward droplets.

c. Maximum z-scored GCaMP8s responses across animals (tone, mean 0.79 z [0.48 - 1.1] bootstrap 95% CI; reward, 3.8 z [3.6 - 4.1]; n = 3 animals, p = 0.005, one-way repeated measures ANOVA).

d. Representative GCaMP8s fluorescence traces from DA axons in response to auditory tones and water reward droplets.

e. Maximum z-scored GCaMP8s responses across animals (tone, mean 0.98 z [0.85 - 1.1] bootstrap 95% CI; reward, 0.92 z [0.77 - 1.1]; n = 3 animals, p = 0.86, one-way repeated measures ANOVA).

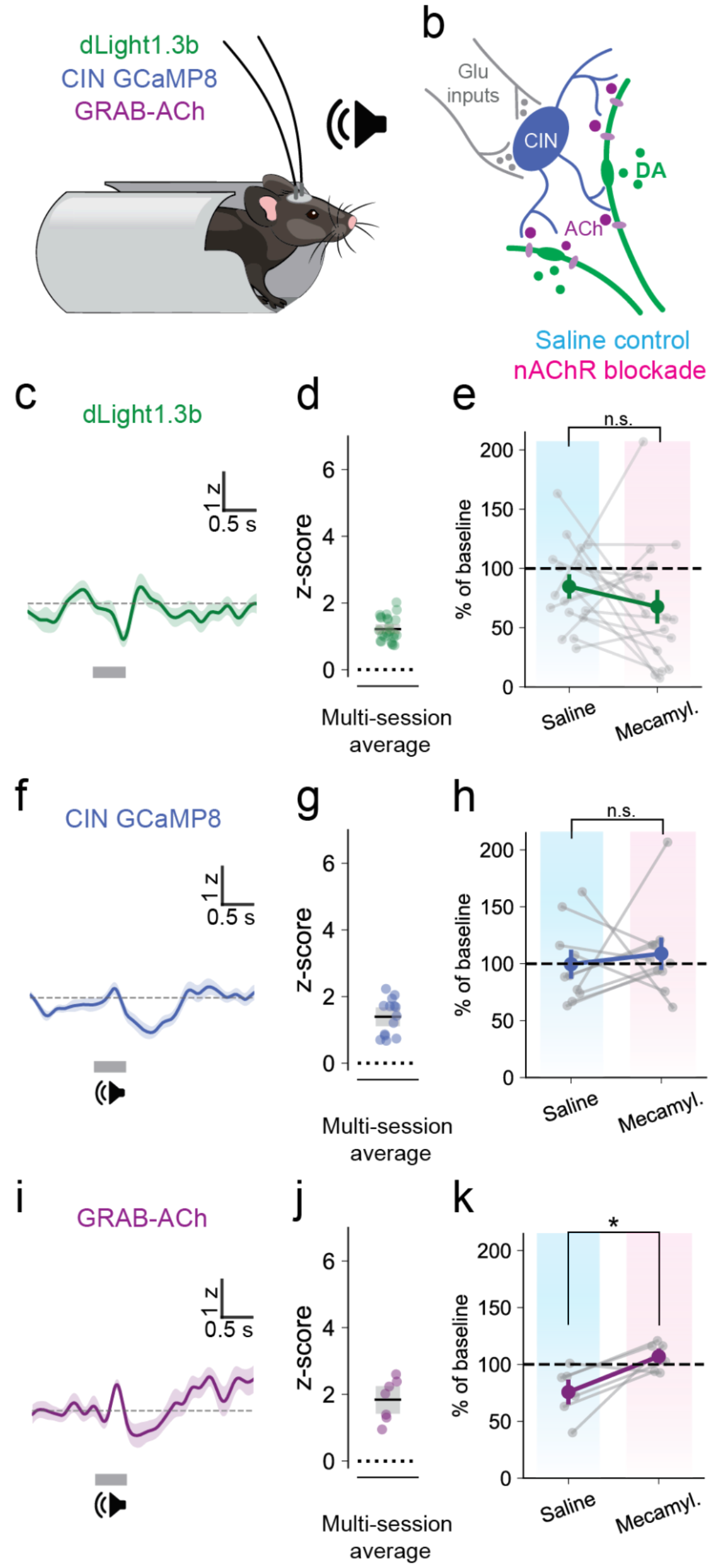

dLight1.3b
CIN GCaMP8
GRAB-ACh
b
Glu inputs
CIN
DA
ACh
Saline control
nAChR blockade
c
dLight1.3b
1 z
0.5 s
d
z-score
Multi-session average
e
n.s.
% of baseline
Saline
Mecamyl.
f
CIN GCaMP8
g
h
i
GRAB-ACh
j
k
*

Supplemental Figure 4. Systemic mecamylamine does not affect tone-evoked CIN or acetylcholine responses

a-b. Implanted mice expressing dLight1.3b, GCaMP8 in CINs or D1-MSNs, or GRAB-ACh were headfixed and presented with 500 ms tones. Mice were presented with the stimuli before and after receiving an intraperitoneal injection of saline or 10 mg/kg mecamylamine.

c-d. Representative traces from one animal expressing dLight1.3b in DMS (c) and maximum evoked z-scored responses across animals (d, n = 21 animals, mean response 1.2 z [1.1 - 1.4] bootstrapped 95% CI).

e. Neither saline nor systemic mecamylamine affected tone-evoked dLight1.3b responses (n = 20 animals; saline mean 85% ± 8.6% SEM; mecamylamine mean 67% ± 12% SEM; p = 0.29, one-way repeated measures ANOVA).

f-g. Representative traces from one animal expressing GCaMP8s in striatal CINs (f) and maximum evoked z-scored responses across animals (g, n = 12 animals mean response 1.4 z [1.1 - 1.7] bootstrapped 95% CI).

h. Neither saline nor systemic mecamylamine affected tone-evoked CIN-GCaMP8 responses (n = 11 animals; saline mean 100% ± 11% SEM; mecamylamine mean 89% ± 12% SEM; p = 0.95, one-way repeated measures ANOVA).

i-j. Representative traces from one animal expressing GRAB-ACh in striatal CINs (f) and maximum evoked z-scored responses across animals (g, mean response 1.8 z [1.4 - 2.3] bootstrapped 95% CI).

k. Saline reduced tone-evoked GRAB-ACh responses mildly more than mecamylamine (n = 6 animals; saline mean 76% ± 9% SEM; mecamylamine mean 107% ± 5% SEM; p = 0.035, one-way repeated measures ANOVA).

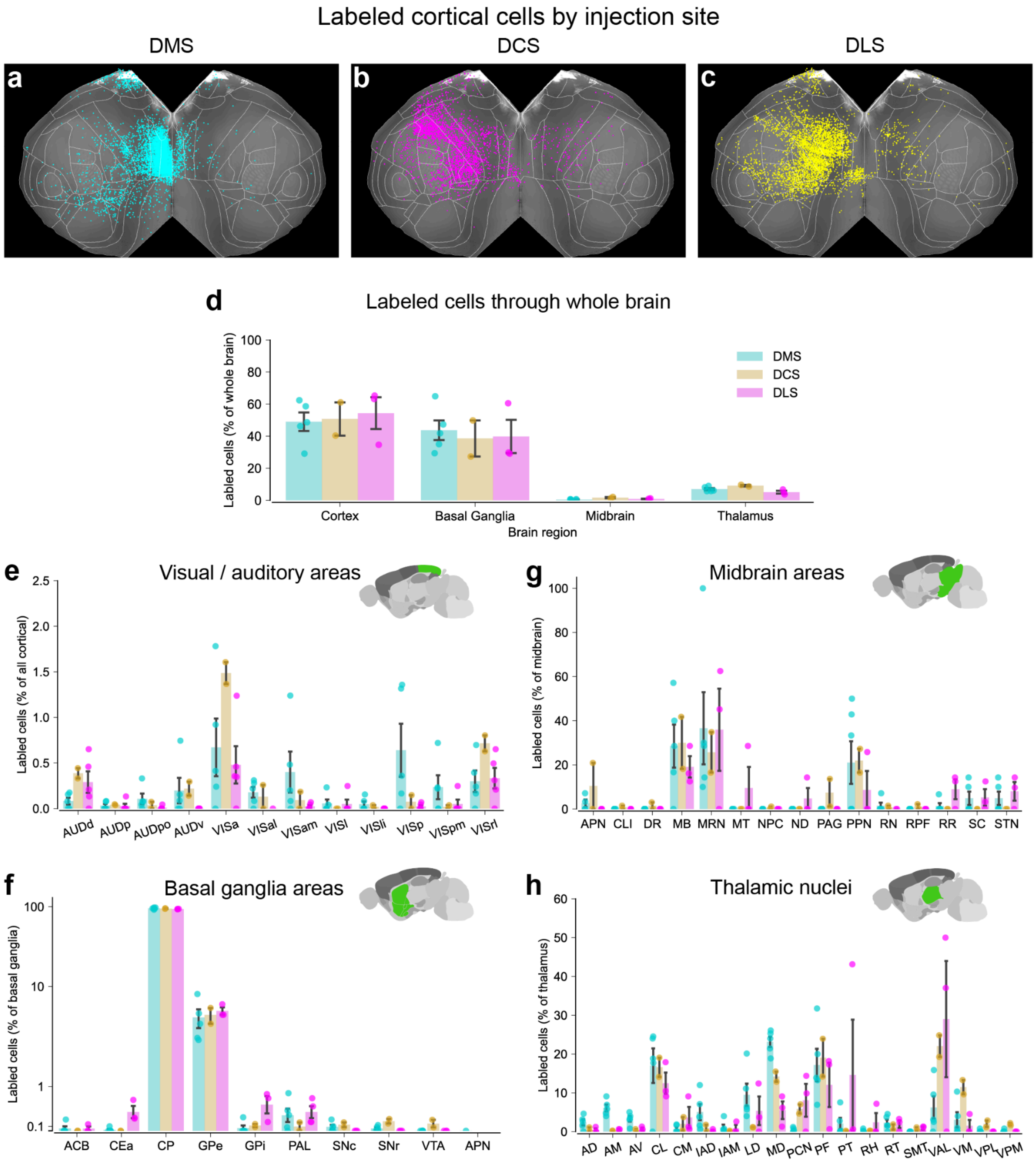

Labeled cortical cells by injection site
DMS
DCS
DLS
a
b
c
d
Labeled cells through whole brain
Labled cells (% of whole brain)
DMS
DCS
DLS
Cortex
Basal Ganglia
Midbrain
Thalamus
Brain region
e
Visual / auditory areas
Labled cells (% of all cortical)
AUDd
AUDp
AUDpo
AUDv
VISa
VISal
VISam
VISl
VISli
VISp
VISpm
VISrl
g
Midbrain areas
Labled cells (% of midbrain)
APN
CLI
DR
MB
MRN
MT
NPC
ND
PAG
PPN
RN
RPF
RR
SC
STN
f
Basal ganglia areas
Labled cells (% of basal ganglia)
ACB
CEa
CP
GPe
GPi
PAL
SNc
SNr
VTA
APN
h
Thalamic nuclei
Labled cells (% of thalamus)
AD
AM
AV
CL
CM
IAD
IAM
LD
MD
PCN
PF
PT
RH
RT
SMT
VAL
VM
VPL
VPM

Supplemental Figure 5. Retrograde rabies tracing of monosynaptic projections to CINs - regional labeling

a-c. Top-down whole brain views of presynaptic rabies labeled cortical neurons providing inputs to CINs in DMS (cyan, left), DCS (yellow, center), and DLS (magenta, right).

d. Labeled cells across the cortex, basal ganglia, thalamus, and midbrain as a percentage of all cells labeled throughout the brain. Regardless of injection site, expression was more widespread across the cortex and basal ganglia than in the midbrain or thalamus.

e. Cells labeled in primary and secondary visual and auditory cortices, as a percentage of all labeled cortical cells. Only a very small percentage of labeled cells (0.5-1.5%) were found in these regions. However, it should be noted that more labeling was found in some secondary visual areas (VISa, VISrl) than in the primary visual cortex.

f. Percent of labeled cells within basal ganglia subregions, as a percent of all labeled cells in basal ganglia. Note that the y-axis is log-scaled. Most labeled cells were located locally within the striatum (CP, caudate-putamen), with additional labeling of the globus pallidus (GPe, GPi). Lesser expression was found throughout the rest of the basal ganglia (ACB, PAL, SNc, SNr, ACB, VTA, CEa).

g. Labeled cells within the midbrain, by subregion, as a percentage of all labeled cells in the midbrain. Labeling in the midbrain was sparse; however, most labeling was seen in MRN, PPN, and other unspecified midbrain areas (MB).

h. Percent of labeled cells within the thalamus by subregion, as a percentage of all labeled cells in the thalamus. Injections across the dorsal striatum produced labeling in CL and PF, while medial injections produced more labeling in MD, and lateral injections produced more labeling in PCN and VAL. For all panels, the area name and abbreviation follow Allen Institute naming conventions (**Table S2**). All error bars are mean ± sem.

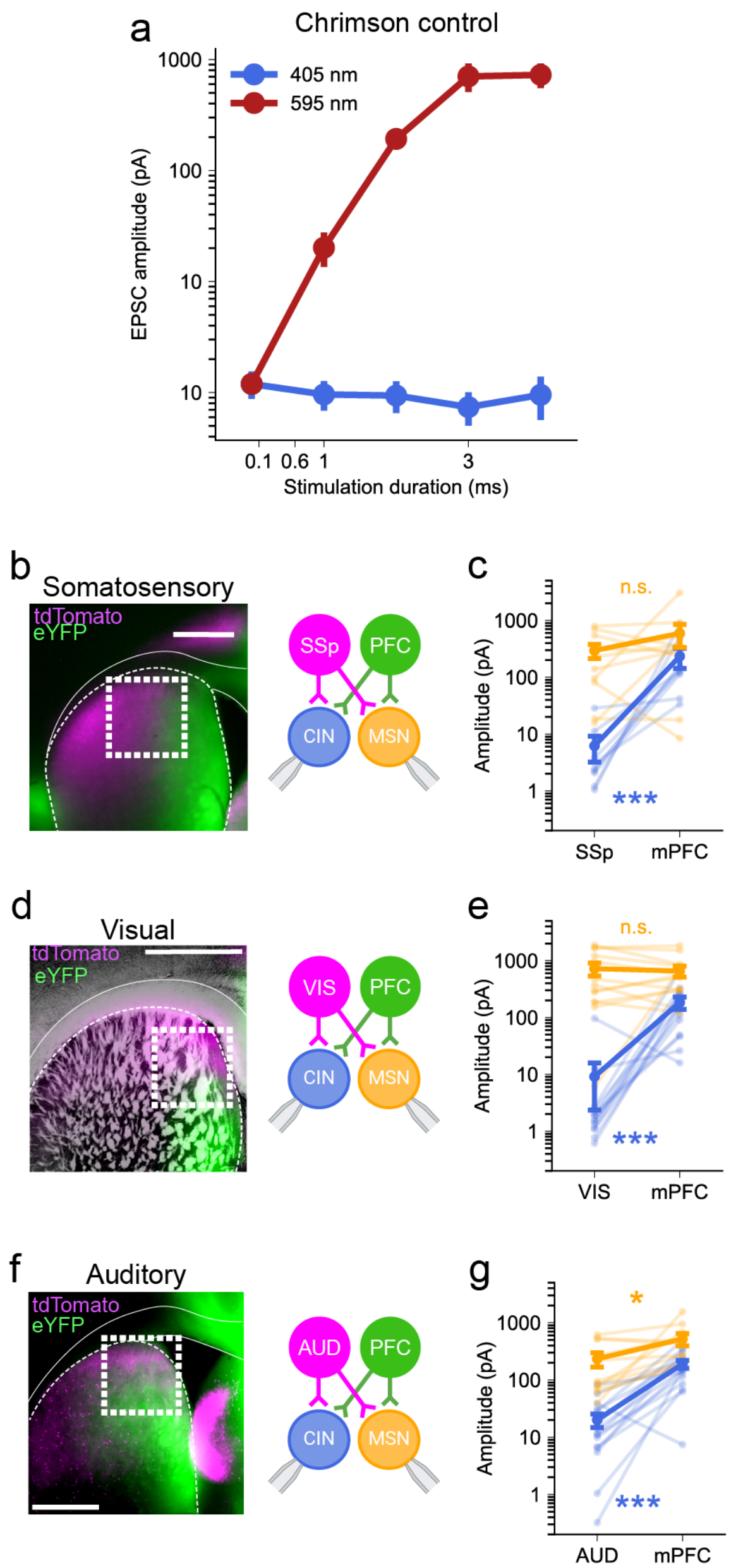
a
Chrimson control
405 nm
595 nm
EPSC amplitude (pA)
Stimulation duration (ms)
b
Somatosensory
tdTomato
eYFP
SSp
PFC
CIN
MSN
c
n.s.
***
Amplitude (pA)
SSp
mPFC
d
Visual
tdTomato
eYFP
VIS
PFC
CIN
MSN
e
n.s.
***
Amplitude (pA)
VIS
mPFC
f
Auditory
tdTomato
eYFP
AUD
PFC
CIN
MSN
g
*
***
Amplitude (pA)
AUD
mPFC

Supplemental Figure 6. Whole-cell voltage clamp data using dual optogenetic stimulation

To directly compare how single cells respond to input from sensory areas and frontal areas, we expressed in the same animal ChrimsonR-tdT in a sensory area and ChR2-eYFP in the prelimbic cortex. This allowed us to record from striatal cells in areas of overlapping expression and measure synaptic responses evoked by 405 nm violet light to drive ChR2-expressing terminals and 590 nm light to drive ChrimsonR-expressing terminals. The opsin's selection was made with the intention of minimizing false positive responses when stimulating sensory cortex axons. ChR2 is activated by blue light, which can also spuriously activate ChrimsonR. Red light, however, provides cleaner activation of only ChrimsonR. Therefore, we expressed ChR2 in prelimbic terminals and ChrimsonR in sensory terminals. This way, red light activation of ChrimsonR would exclusively activate sensory terminals. If the expression were swapped, blue light stimulation of sensory terminals would also stimulate the stronger projections from the prelimbic cortex, resulting in false positives.

a. Effects of 405 nm (off-target) and 595 nm (on-target) stimulation in an animal that only expressed ChrimsonR. 405 nm violet light does not activate ChrimsonR terminals.

b, d, f. Coronal brain sections from ChAT-tdTomato reporter mice expressing ChR2-eYFP in PL (green) and ChrimsonR-tdTomato in either SSp (a), VISp (c), or AUDp (e, magenta). Dual opsin optogenetic stimulation was used to probe for evoked synaptic responses while recording from striatal CINs (blue) and MSNs (orange) in areas of axonal overlap (white squares). Excitatory synaptic responses were measured using whole-cell voltage-clamp electrophysiology. All white scale bars are 1 mm.

c, e, g. Mean amplitude of EPSCs recorded from CINs (blue) and MSNs (orange) following paired optogenetic stimulation of PL and sensory corticostriatal projections. All tests were two-sided Mann-Whitney U. SSp MSNs: $n = 10$ cells/3 animals, $p = 0.65$; SSp CINs: $n = 9/2$, $p = 0.0004$; VIS MSNs: $n = 12$ cells / 3 animals, $p = 0.75$; VIS CINs: $n = 14/5$, $p < 0.0001$; AUD MSNs: $n = 17/3$, $p = 0.06$; AUD CINs: $n = 17/3$, $p < 0.0001$. Dark lines represent population mean ± SEM, and light dots represent data from each cell recorded.

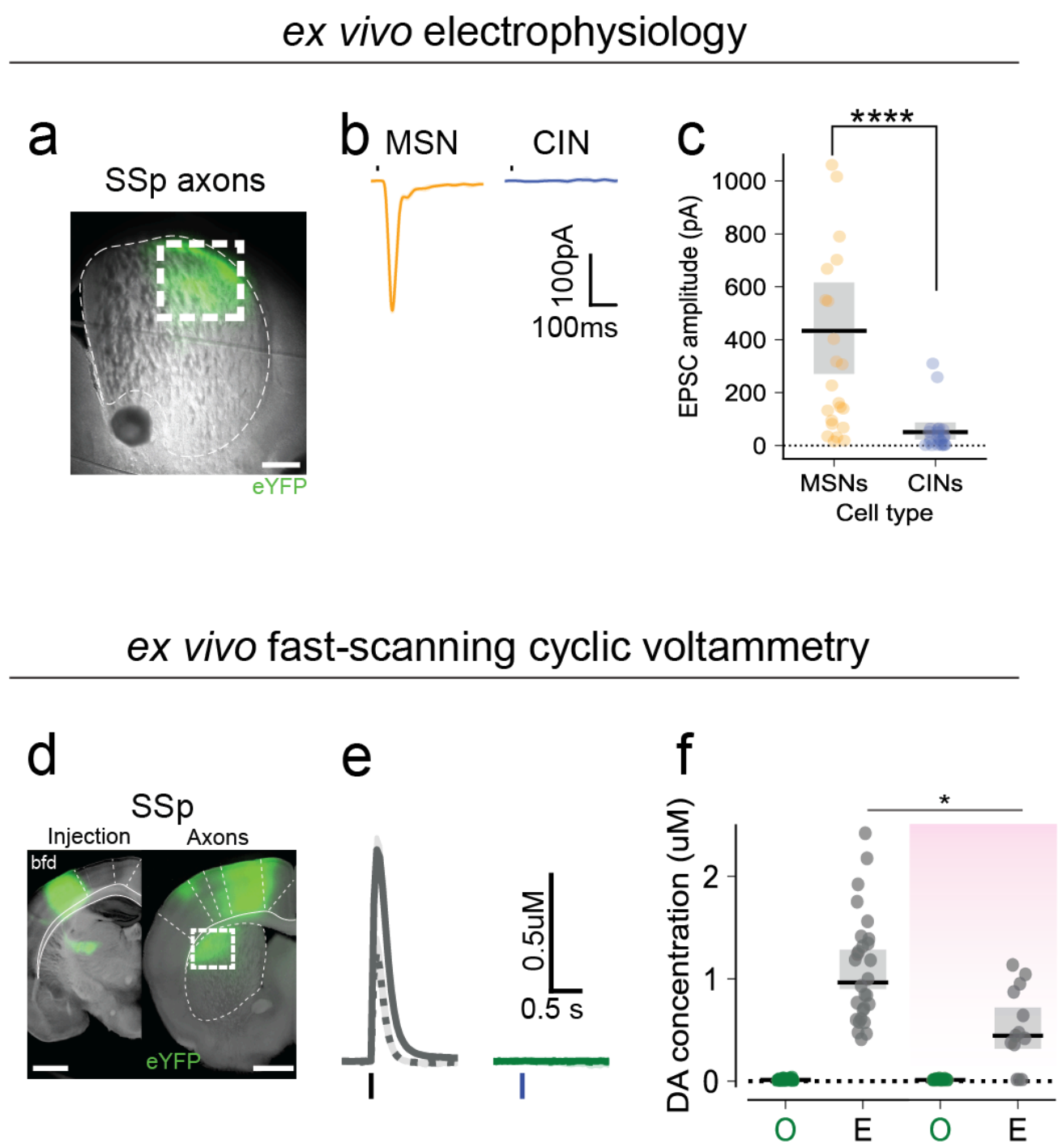
ex vivo electrophysiology
a
SSp axons
eYFP
b
MSN
CIN
100pA
100ms
c
****
EPSC amplitude (pA)
1000
800
600
400
200
0
MSNs
CINs
Cell type
ex vivo fast-scanning cyclic voltammetry
d
SSp
Injection
Axons
bfd
eYFP
e
0.5uM
0.5 s
f
*
DA concentration (uM)
2
1
0
O
E
O
E

Supplemental Figure 7. Somatosensory terminals in dorsolateral striatum do not recruit CINs or produce cholinergic-dependent dopamine

Mice with labeled CINs (ChAT-IRES-Cre x tdTomato) were injected with either ChR2-eYFP or ChrimsonR-tdTomato in primary somatosensory cortex (SSp). Brains were extracted and sliced for ex vivo whole-cell patch clamp electrophysiology and fast-scanning cyclic voltammetry following a minimum four-week incubation.

a. Representative image of ChR2-eYFP labeled SSp projections in striatum. Recordings were conducted within the white box. Scale bar is 1 mm.

b. Representative traces of excitatory postsynaptic currents recorded from an MSN and a CIN in DLS.

c. Average MSN EPSC amplitudes evoked via optogenetic stimulation of SSp projections (MSN mean 433 pA [270 - 622] bootstrapped 95% CI n = 16 slices from 4 mice; CIN mean 50 pA [22 - 87], n = 14 slices from 5 mice; $p < 0.00001$, one-way ANOVA).

d. Left, ChR2-eYFP expression (green) in SSp. Right, ChR2-eYFP expressing projections (green) from SSp in dorsomedial striatum. The white box shows the site of dopamine recordings. Scale bars are 1 mm.

e. Single-slice average of dopamine transients measured with fast-scanning cyclic voltammetry (FSCV) and evoked by either optogenetic stimulation of corticostriatal projections (green) or electrical stimulation (gray) in the same recording site. Dashed lines show the remaining dopamine responses after washing in a nicotinic receptor blocker (1 μM DHβE). Traces are mean and shaded areas are ± SEM).

f. Average amplitudes of dopamine transient in DLS elicited by electrical stimulation (gray bars, in ACSF mean 1.09 uM [0.90 - 1.3] bootstrapped 95% CI, n = 28 slices from 8 animals; in DHBE mean 0.52 uM [0.32 - 0.72], n = 13 slices from 5 animals; ACSF vs DHBE p = 0.005, Mann-Whitney U) and optogenetic stimulation (green bars, in ACSF mean 0.013 uM [0.011 - 0.016]; in DHBE mean 0.014 [0.011 - 0.017]) before and after DHβE bath application (pink shade). Dots represent data from individual slices. Lines and shaded bars are mean ± sem.

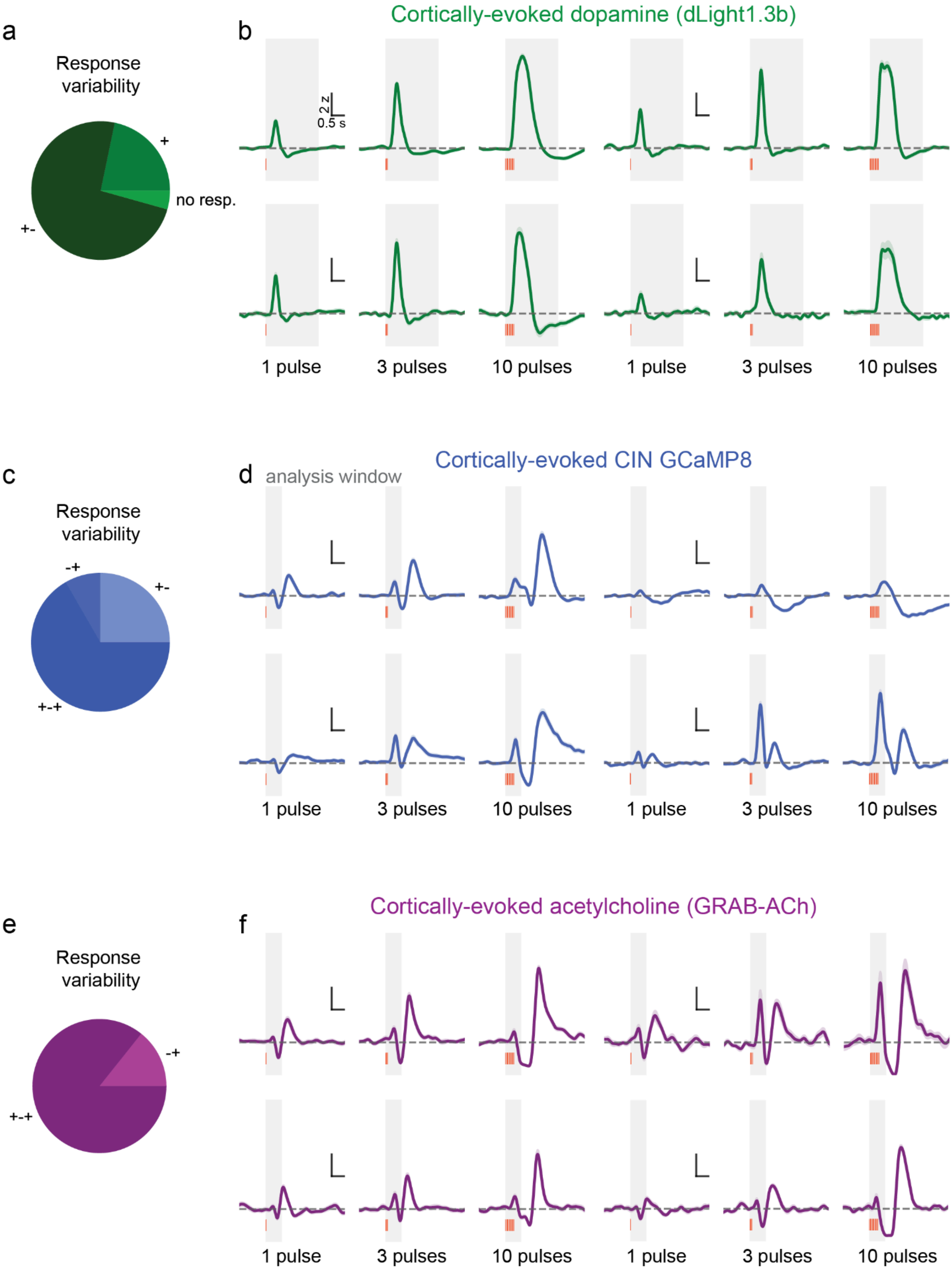
a
Response
variability
+
no resp.
+-
b
Cortically-evoked dopamine (dLight1.3b)
2 z
0.5 s
1 pulse
3 pulses
10 pulses
1 pulse
3 pulses
10 pulses
c
Response
variability
-+
+-
+-+
d
analysis window
Cortically-evoked CIN GCaMP8
1 pulse
3 pulses
10 pulses
1 pulse
3 pulses
10 pulses
e
Response
variability
-+
+-+
f
Cortically-evoked acetylcholine (GRAB-ACh)
1 pulse
3 pulses
10 pulses
1 pulse
3 pulses
10 pulses

Supplemental Figure 8: Heterogeneity in cortically-evoked responses

a. Percentages of animals exhibiting monophasic (+), biphasic (+-) or no dopamine response to cortical stimulation.

b. Example traces of cortically-evoked dopamine responses from four animals, demonstrating heterogeneity in the second phase of the response. Gray shaded region represents the 2 s time window used for analysis.

c,e. Percentages of animals exhibiting biphasic (+-), inverse biphasic (-+), or triphasic CIN GCaMP © or GRAB-ACh (e) responses to cortical stimulation.

d, f. Example traces of cortically-evoked CIN GCaMP (d) or GRAB-ACh (f) responses from four animals, demonstrating heterogeneity across all three phases of the response. Because of this heterogeneity, analysis was limited to 0.6s after stimulation, which consistently captured the first phase of the response.

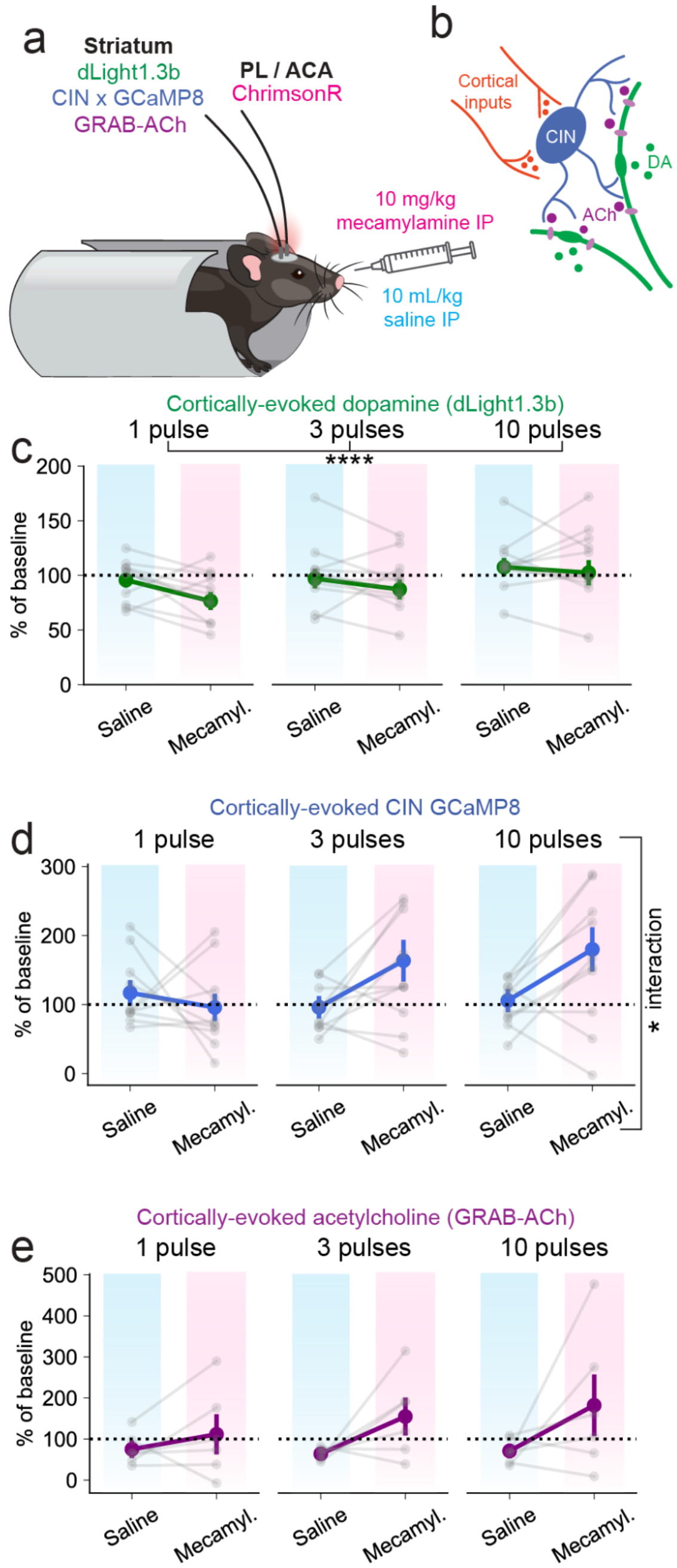

a
Striatum
dLight1.3b
CIN x GCaMP8
GRAB-ACh
PL / ACA
ChrimsonR
10 mg/kg
mecamylamine IP
10 mL/kg
saline IP
b
Cortical
inputs
CIN
DA
ACh
Cortically-evoked dopamine (dLight1.3b)
c
1 pulse
3 pulses
10 pulses
****
% of baseline
Saline
Mecamyl.
Cortically-evoked CIN GCaMP8
d
* interaction
Cortically-evoked acetylcholine (GRAB-ACh)
e

Supplemental Figure 9: Systemic saline and mecamylamine do not significantly reduce optogenetically evoked responses

a-b. Implanted mice expressing dLight1.3b, GCaMP8 in CINs, or GRAB-ACh in DMS were headfixed and received optogenetic stimulation of PL / ACA striatal terminals (see Figure 6, Methods). Recordings were conducted before and after injecting the animal intraperitoneally with 10 mL / kg sterile saline (vehicle) or 10 mg / kg mecamylamine.

c. Systemic saline and mecamylamine had limited effects on cortically-evoked dopamine responses, though there was an effect of stimulation pattern (n = 11 mice, treatment p = 0.31, stimulation p = 0.00007, interaction p = 0.18, two-way repeated measures ANOVA).

d. Systemic saline and mecamylamine had no effect on cortically-evoked CIN GCaMP responses, though there was an effect of the interaction between treatment and stimulation pattern (n = 10 animals, treatment p = 0.13, stimulation p = 0.48, interaction p = 0.048, two-way repeated measures ANOVA).

e. Systemic saline and mecamylamine had no effect on cortically-evoked GRAB-ACh responses (n = 5 animals, treatment p = 0.08, stimulation p = 0.57, interaction p = 0.55, two-way repeated measures ANOVA).

In all plots, individual gray lines represent data from one animal. Dark lines represent the mean and sem across all animals.

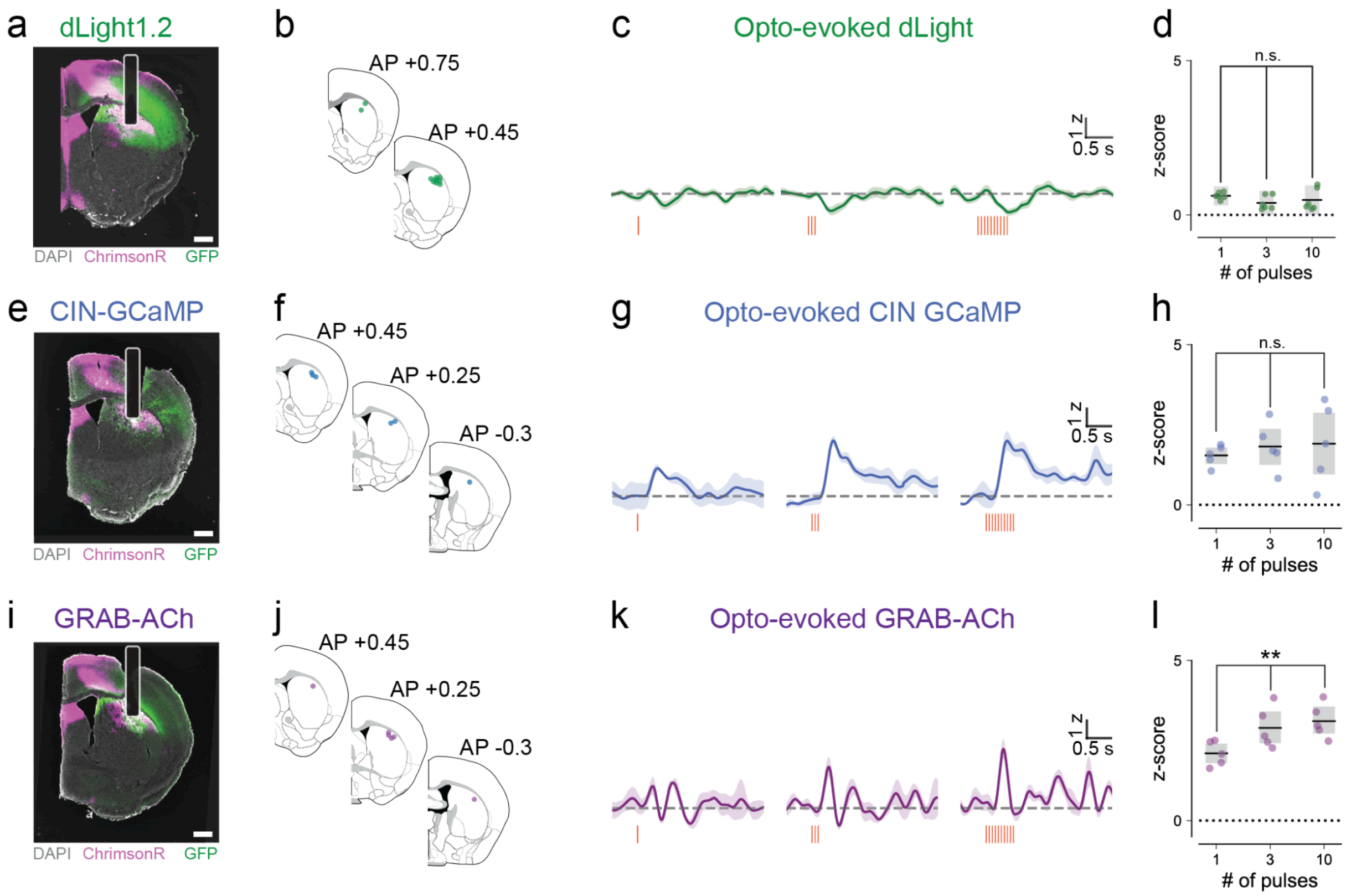

Supplemental Figure 10: Somatosensory cortex drives CINs and acetylcholine release in dorsolateral striatum, but not dopamine

a,e,i. Images from ChAT-IRES-cre or wildtype mice were injected with ChrimsonR in primary somatosensory cortex and with dLight1.2, DIO-GCaMP8s, or GRAB-ACh in dorsolateral striatum (green). Animals were implanted with a headpost and bilateral fiber optic cannula in DLS, allowing for stimulation of SSp terminals (magenta). Scale bars are 0.5mm.

b, f, j. Fiber placements in DLS, n = 6 animals per sensor. Animals expressing dLight1.3b were implanted bilaterally. The average of the two hemispheres is presented as one datapoint. Fiber placements are displayed on Allen Brain Atlas plates[73].

c, g, k. Representative traces from three animals expressing dLight1.3b, GCaMP8s in CINs, or GRAB-ACh in DLS. Optogenetic stimulation protocol included a single 10ms pulse of 590 nm red light, trains of 3 pulses at 20 Hz, and trains of 10 pulses at 20 Hz, with each stimulation occurring 30s apart.

d, h, l. Maximum z-scored optogenetically-evoked responses across animals expressing dLight1.3b (d), CIN GCaMP (h), and GRAB-ACh (i). Stimulation intensity did not affect dLight1.3b or CIN GCaMP (dLight $p = 0.07$, CIN GCaMP $p = 0.48$, one-way repeated measures ANOVA), though it did have an effect on GRAB-ACh responses ($p = 0.0004$, one-way repeated measures ANOVA).

Supplemental Table 1: Fiber photometry animals & viral expression

Mouse	Sex	Age (m)	Genotype	Left Hem.	Right Hem.	Chrimson
**Ch-18**	**F**	9	WT	dLight1.2b	dLight1.2b	SSp
**Ch-25**	M	9	WT	dLight1.2b	dLight1.2b	SSp
**Ch-26**	M	9	WT	dLight1.2b	dLight1.2b	SSp
**HG-01**	M	6	WT	dLight1.2b	dLight1.2b	SSp
**HG-02**	M	6	WT	dLight1.2b	dLight1.2b	SSp
**HG-03**	M	6	WT	dLight1.2b	dLight1.2b	SSp
**HG-04**	M	4	ChAT-Cre		GCaMP8s	PL
**HG-05**	M	6	ChAT-Cre		GCaMP8s	PL
**HG-06**	M	7	ChAT-Cre	ACh3.0	GCaMP8s	PL
**HG-08**	**F**	3	ChAT-Cre	ACh3.0	GCaMP8s	PL
**HG-09**	**F**	3	ChAT-Cre	ACh3.0	GCaMP8s	PL
**HG-10**	**F**	3	ChAT-Cre	ACh3.0	GCaMP8s	PL
**HG-11**	**F**	6	ChAT-Cre	GCaMP8f	dLight1.3b	PL
**HG-12**	**F**	6	ChAT-Cre	GCaMP8f	dLight1.3b	PL
**HG-14**	M	6	ChAT-Cre	GCaMP8f	dLight1.3b	PL
**HG-15**	**F**	3	ChAT-Cre	GCaMP8f	GCaMP8f	ACA
**HG-16**	**F**	3	ChAT-Cre	GCaMP8f	GCaMP8f	ACA
**HG-17**	M	3	ChAT-Cre	GCaMP8f	dLight1.3b	ACA
**HG-18**	M	5	WT	ACh3.0	dLight1.3b	ACA
**HG-19**	**F**	5	WT	ACh3.0	dLight1.3b	ACA
**HG-20**	M	4	WT	ACh3.0	dLight1.3b	ACA
**HG-21**	**F**	5	DAT-Cre	GCaMP8s		PL
**HG-22**	M	6	DAT-Cre	GCaMP8s		PL
**HG-23**	M	6	WT	dLight1.3b		PL
**HG-24**	M	6	WT	dLight1.3b*		PL
**HG-25**	M	7	ChAT-Cre	GCaMP8s	ACh3.0	SSp
**HG-26**	M	7	ChAT-Cre	GCaMP8s	ACh3.0	SSp
**HG-27**	M	7	ChAT-Cre	GCaMP8s	ACh3.0	SSp
**HG-28**	M	3	DAT-cre	GCaMP8s		PL
**HG-29**	M	3	DAT-cre	GCaMP8s		PL
**HG-30**	M	3	DAT-cre	GCaMP8s		PL
**HG-31**	M	3	ChAT-Cre	GCaMP8s	ACh3.0	SSp
**HG-32**	M	3	ChAT-Cre	GCaMP8s	ACh3.0	SSp
**HG-33**	M	3	ChAT-Cre	GCaMP8s	ACh3.0	SSp

Supplemental Table 1 continued: Fiber photometry animals & viral expression

Mouse	Sex	Age (m)	Genotype	Left Hem.	Right Hem.	Chrimson
**HG-34**	**F**	3	DAT-cre	GCaMP8s		PL
**HG-35**	**F**	3	DAT-cre	GCaMP8s		PL
**HG-39**	M	3	WT	dLight1.3b*		PL
**HG-40**	M	3	WT	dLight1.3b*		PL
**HG-41**	M	3	WT	dLight1.3b*		PL
**HG-42**	**F**	3	WT	dLight1.3b*		PL
**HG-43**	**F**	3	WT	dLight1.3b*		PL
**HG-44**	**F**	3	WT	dLight1.3b*		PL
**RR-03**	M	4	WT	dLight1.3b	dLight1.3b	PL
**RR-05**	M	4	WT	dLight1.3b	dLight1.3b	PL
**RR-07**	M	4	WT	dLight1.3b	dLight1.3b	PL
**RR-09**	M	4	WT	dLight1.3b	dLight1.3b	PL
**LA-11**	M	7	D1-Cre	GCaMP8s	GCaMP8s	
**LA-12**	M	7	D1-Cre	GCaMP8s	GCaMP8s	
**LA-13**	M	7	D1-Cre	GCaMP8s	GCaMP8s	
**LA-15**	M	9	D1-Cre	GCaMP8s	GCaMP8s	
**LA-16**	M	9	D1-Cre	GCaMP8s	GCaMP8s	
**LA-18**	**F**	8	D1-Cre	dLight1.3b	GCaMP8s	
**LA-19**	**F**	8	D1-Cre	dLight1.3b	GCaMP8s	
**LA-20**	**F**	8	D1-Cre	GCaMP8s	dLight1.3b	
**LA-28**	M	6	D1-Cre	PFC axon ipsi	PFC axon contra	
**LA-29**	M	6	D1-Cre	PFC axon ipsi	PFC axon contra	
**LA-30**	**F**	6	D1-Cre	PFC axon ipsi	PFC axon contra	
**LA-33**	M	6	Pdyn-Cre	PFC axon ipsi	PFC axon contra	
**LA-34**	**F**	6	Pdyn-Cre	PFC axon ipsi	PFC axon contra	
**LA-35**	**F**	6	Pdyn-Cre	PFC axon ipsi	PFC axon contra	
**LA-36**	**F**	6	Pdyn-Cre	PFC axon ipsi	PFC axon contra	

* optofluid cannula

Supplemental Table 2: Allen Institute Brain Region Nomenclature

Abbreviation	
**ACA**	Anterior Cingulate Area
**ACB**	Accumbens
**AD**	Anterodorsal Nucleus
**AI**	Agranular Insular Area
**AM**	Anteromedial Nucleus
**APN**	Anterior Pretectal Nucleus
**AUDp**	Primary Auditory Area
AUDd	*Dorsal Auditory Area*
AUDpo	*Posterior Auditory Area*
AUDv	*Ventral Auditory Area*
**AV**	Anteroventral Nucleus
**BG**	Basal Ganglia
**CEa**	Central Amygdalar Nucleus
**CL**	Claustrum
**CLI**	Central Linear Nucleus Raphe
**CM**	Central Medial Nucleus
**CP**	Caudoputamen
**CTX**	Cortex
**DR**	Dorsal Nucleus Raphe
**ECT**	Ectorhinal Area
**GPe**	Globus Pallidus External
**GPi**	Globus Pallidus Internal
**GU**	Gustatory Area
**IAD**	InfralimbicArea
**IAM**	Interanteromedial Nucleus
**IL**	Infralimbic Area
**LD**	Lateral Dorsal Nucleus
**MB**	Midbrain
**MD**	Mediodorsal Nucleus
**MRN**	Midbrain Reticular Nucleus
**MT**	Medial Terminal Nucleus
**ND**	Nucleus of Darkschewitsch
**NPC**	Nucleus of the Posterior Commissure
**MOp**	Primary Motor Area
**MOs**	Secondary Motor Area
**ORB**	Orbital Area
**PAG**	Periaqueductal Gray
**PAL**	Pallidum
**PCN**	Paracentral Nucleus
**PERI**	Perirhinal Area
**PF**	Parafascicular Nucleus

Abbreviation	
**PL**	Prelimbic Area
**PPN**	Pedunculopontine Nucleus
**PT**	Parataenial Nucleus
**RH**	Rhomboid Nucleus
**RN**	Red Nucleus
RPF	Retroparafascicular Nucleus
**RT**	Reticular Nucleus
**RSP**	Restrosplenial Area
**SMT**	Submedial Nucleus
**SNc**	Substantia Nigra Pars Compacta
**SNr**	Substantia Nigra Pars Reticulata
**SSp**	Primary Somatosensory Area
SSp-bfd	Barrelfield Area
SSp-ll	Lower Limb Area
SSp-m	Mouth Area
SSp-n	Nose Area
SSp-tr	Trunk Area
SSp-ul	Upper Limb Area
SSp-un	Undefined Area
**SSs**	Secondary Somatosensory Area
**TEa**	Temporal Association Area
**TH**	Thalamus
**VAL**	Ventral Anterior-lateral Complex
**VISC**	Visceral Area
**VISp**	Primary Visual Area
VISa	Anterior Visual Area
VISal	Anterolateral Visual Area
VISam	Anteromedial Visual Area
VISpm	Posteromedial Visual Area
VISrl	Rostrolateral Visual Area
VISl	Lateral Area
**VM**	Ventral Medial Nucleus
**VPL**	Ventral Posterolateral Nucleus
**VPM**	Ventral Posteromedial Nucleus
**VTA**	Ventral Tegmental Area
