# Supplementary material for: Cholinergic-dependent dopamine signals in mouse dorsomedial striatum are regulated by frontal but not sensory cortices
Source: Nat Commun. 2026 Sep 2;17:9601. doi: 10.1038/s41467-026-77168-x (PMC13554099; doi:10.1038/s41467-026-77168-x)
Supplement: Supplementary file 3 [file 41467_2026_77168_MOESM3_ESM.pdf]

nature portfolio

Peer Review File

Cholinergic-dependent dopamine signals in mouse dorsomedial striatum are regulated by frontal but not sensory cortices

Corresponding Author: Dr Veronica Alvarez

This file contains all reviewer reports in order by version, followed by all author rebuttals in order by version.

Version 0:

Reviewer comments:

Reviewer #1

(Remarks to the Author)
This study from Goldbach and colleagues tackles a question of great interest in the field of basal ganglia research: how is the release of DA from axons regulated locally within the striatum by cholinergic interneurons (CINs)? This question has been the subject of many papers over the past 10 years, with good consensus amongst studies performed in rodent brain slices, but some disagreement with studies evaluating the prevalence of such mechanism in vivo. This manuscript describes experiments taking place both in slices and in vivo, all from mice. It makes a number of compelling observations: 1) salient sensory stimuli strongly drive DA release in the dorsomedial striatum (DMS) in vivo, 2) this DA release partially depends on nicotinic receptor signaling, 3) frontal cortico-striatal inputs provide strong excitatory drive onto DMS CINs in brain slices, but sensory cortico-striatal inputs do not, 4) stimulation of frontal cortico-striatal inputs drives CIN-dependent DA release in brain slices, but stimulation of sensory cortico-striatal inputs does not, and 5) optogenetic stimulation of frontal cortical axons is sufficient to drive striatal DA release in vivo, in a manner that is partially sensitive to nicotinic receptor blockade.

Overall, this study is of high quality: it is very clearly written, the references are adequate and the figures are crystal clear. The finding that frontal (but not sensory) cortico-striatal axons drive CINs (points 3 and 4 above) in brain slices is novel and ought to be of great interest to many. This study also provides a detailed retrograde tracing map of presynaptic inputs to CINs in different regions of dorsal striatum, which will be a great resource to the field (even if another similar experiment had been performed earlier, though not with the same attention to detail). The in vivo results are compelling too, and make a strong case that cortical drive can evoke DA release. The mechanisms involved, however, remain somewhat less clear. A case can certainly be made for the mechanism favored by the authors, but alternative possibilities are not excluded. As such, this study (in its current form) does not settle the debate in vivo. I still think that this study has great value and suggest experiments that, if performed, will greatly strengthen the manuscript, and if not, ought to be acknowledged and given ample space/discussion to help guide future experiments.

First, regarding the novel finding that sensory areas cortical afferents to striatum do not innervate CINs. It was not clear to me whether this reflects a novel finding that generalizes to all striatal regions (i.e. cortico-striatal afferents from sensory regions NEVER innervate CINs anywhere in striatum, but frontal cortico-striatal afferents do), or whether this reflects the choice made by the authors to focus their recordings in DMS. In other words, how much of this ‘specificy’ stems from differential innervation of striatum by cortical areas? Indeed, the retrograde tracing studies clearly make the case that the majority of cortico-striatal afferents to DMS originate in frontal cortices, not sensory, while the opposite applies to more ‘sensory’ striatal regions such as DLS. Frontal cortical afferents are therefore unlikely to drive CINs in DLS either, which the title of the study does not reflect. This issue would greatly benefit from clarification throughout the text, and the title and conclusions should modified to specify “in DMS”.

On this same topic, other studies have reported cortically-evoked and CIN-dependent release of dopamine in DLS in brain slices when stimulating motor cortical axons, or thalamic axons. Assuming that sensory afferents are not capable of driving CINs anywhere in striatum, including in regions where their innervation pattern in densest, they would be the only afferents incapable of doing so. This should be the most exciting takeaway, with interesting molecular/developmental implications. Currently, the title and text seem to implicate a specialized role for frontal/executive cortical areas in controlling DA release, but this unlikely to be true, even in DMS where motor and thalamic afferents also exist (but their contribution was not

investigated). Further refinements of the title, text and conclusions would therefore be needed for clarity.

Second, regarding the in vivo experiments. A major limitation of this study is that the effects of systemic nAChR blockade are solely interpreted in the context of CIN-evoked DA release from axons locally within the striatum. While nAChRs do exist on DA axon terminals (and can drive DA release in slices, as show here and in many other studies), this is not the only site of action of nAChRs. They are, for example, strongly expressed on the soma and dendrites of DA neurons in the midbrain, which receive strong cholinergic inputs from pedunculopontine and laterodorsal tegmental nuclei (see PMID: 19523928, 24881574 for review). This contribution cannot be excluded. Figure 2 does a noble job of excluding gross alterations in sensory perception and the recruitment of dorsal striatal circuits, but the somatic response of midbrain DA neurons are the elephant in the room! As the debate between somatic vs axonal regulation of DA release in in vivo drags on, it is no longer sufficient to ignore what happens in the cell body region. If the authors performed calcium imaging in the midbrain and 1) found light-evoked responses (which they certainly will) and 2) a substantial decrease in this response following systemic nAChR antagonist treatment (which they may), then the conclusions of this study would need to be revised, but the impact would remain, as there is a clear need to figure out what is goes on in vivo. The conclusions would be interesting and compelling in either case.

Figures 1 and 2 make the case that sensory stimuli drive DA release in striatum in a nAChR-dependent manner. At face value, this seems to run counter to the title and main point of the slice experiments, which show that sensory inputs do not drive CINs in DMS. The paper then dives into mechanisms local to striatum, but no attempt is made to loop back to this original set of experiments; we are left wondering how to interpret the findings. Is it the case that light flashes recruit frontal cortical areas, which go on to drive DMS CINs, and DA release? Or is it a completely unrelated circuit, possibly originating in the midbrain cholinergic system? The value of these findings may be limited without any investigation of what happens in frontal cortical neurons, and most importantly, midbrain DA neurons.

Figure 6 is the most direct test of the proposed model, but it still avoids the elephant in the room and glosses over details that directly speak to the prevalence of the proposed model in vivo:
- A clear case is made that optogenetic stimulation of frontal cortex drives DA release in the striatum in proportion to pulse number. However, this response is barely affected by systemic nAChR block for 2 out of 3 pulse patterns, and not at all for the third, suggesting that nAChR signaling accounts for a minority of the DA released in this assay. Where does larger nAChR-independent component come from?
- DA release scales with stimulation intensity, but CIN and ACh responses do not.
- Single pulses drive DA release, but do not significantly engage CINs or ACh release (at short latencies) in DMS.
- Cortical stimulation evokes a delayed pause in CIN and dip in ACh followed by a pronounced (and even more delayed) burst in CIN activity and ACh release. This delayed component does not seem to affect DA release at all, further calling into question the importance of local nAChR-evoked DA release in vivo.
- No attempt is made to exclude the null hypothesis that cortical stimulation drives DA neuron activity in the midbrain in a manner that is mildly affected by systemic nAChR antagonist treatment. Anatomical evidence supports direct innervation of DA neurons by cortical neurons (PMID: 22681690).

The impact of this study would be greatly enhanced by performing calcium imaging of DA neurons in SNc/VTA in this task. But short of this, the authors ought to devote considerable space in their discussion to mention these important caveats and to suggest these and other future experiments that would be needed to get to the bottom of this important issue.

MINOR COMMENTS:
- In vivo N is specified for hemispheres and mice: which is used for statistical analyses? If hemispheres are used, then statistical tests ought to factor in that multiple hemispheres are taken from the same mouse (i.e. each N is not a statistically independent measure).
- Can the authors provide non-experts a sense for how loud or bright the sensory stimuli used in Fig 1 are? Might they be considered equally salient, or aversive, or startling? Could this be a factor in explaining the difference between light and sound stimuli?
- I am surprised that animals (and DA responses) don't habituate to sensory stimuli that are not paired with rewards. There is evidence that sensory-evoked DA responses decrease with time under similar conditions (PMID: 28054919; 39415006). What may the difference be here?
- Line 354: why is it difficult to rule out the engagement of DA neurons following cortical stimulation? As shown here, photometric imaging of calcium in well within your reach.
- Ref 23 and 32 are now published.
- Line 399: This study does not study ACh-mediated control over DA throughout learning either; this distinction should therefore not be made to highlight the value of this work over others'.
- Line 404: Please clarify what is meant by 'proprietary' role of frontal cortex, and how ref. 52 (in prior sentence) implicating thalamic inputs in ACh-mediated DA release support such a 'proprietary' role.
- Methods: Why are Pvalb-Cre and Sst-Cre lines listed for 'MSN' recordings??
- Methods: Why is 405 nm light delivered at a lesser power than 470 nm light, especially since 405 nm light barely generates any signal and penetrates tissue less well than 470 nm light? How is the 405 nm signal used to correct or clean up the 470nm signal? More details of how photometry signals are analyzed using these packages to obtain df/f values (delta from what?) and z-scored is important.

Reviewer #2

(Remarks to the Author)
The manuscript by Goldbach et al. examines the afferent-specificity of evoked CIN-mediated dopamine release in the dorsomedial striatum. Using a combination of in vivo imaging, circuit tracing, slice electrophysiology and neuromodulator biosensors/measurements the authors show that frontal cortical regions (in particular the anterior cingulate and prelimbic) can reliably evoke CIN-mediated dopamine release in the dorsomedial striatum, whereas sensorimotor regions cannot. This is due to heterogenous anatomical and functional connectivity of diverse cortical afferents to CINs in the dorsal striatum. This is an exciting and important study, with profound implications for both reinforcement learning and numerous dopamine-related disorders. In my opinion, a few issues should be addressed to strengthen the intriguing conclusions of this important study.

Major:
1. The authors show in figure 1 that CIN-dependent dopamine release is stimulus-specific in the dorsomedial striatum: visual stimuli evoke nAChR-dependent dopamine release, whereas auditory stimuli do not. The interpretation is that the auditory stimulus used does not engage CINs as efficiently as the visual stimulus does. In figure 2 the authors then nicely show how a visual stimulus promotes CIN activity and ACh and dopamine release. The auditory stimulus may not evoke dopamine release in the dorsomedial striatum, but does it promote CIN activity and ACh release? Mechanistically, this would be important to know.

2. Related to point 1, previous studies have shown that auditory stimuli can produce complex responses in CINs (or at least in “tonically active neurons”) in non-human primates. How do the authors think these observations fit with the data in this manuscript? I don’t think this needs to be addressed with new experiments, but a discussion would be helpful.

3. For slice electrophysiology experiments in figure 4, “CIN and MSN recordings were made from the striatal region receiving labeled projections.” This makes sense, but it does not directly translate to the general question of what is going on in the dorsomedial striatum. For example, while since SSp axons are mostly found in the dorsolateral striatum, were any CINs recorded in response to SSp stimulation in the dorsomedial striatum, the region where ACh and dopamine release measures were made? I understand that the expectation is that responses would be very small if axon density is low, but this detail is central to the paper’s conclusions.

4. The authors nicely show in figure 6 that stimulation of PL/ACA inputs evokes CIN-dependent dopamine release in the dorsomedial striatum in vivo (and in earlier figures show that anatomical/functional connectivity of sensory and motor cortex to CINs in this region is minimal). A useful control in figure 6 would be to show that stimulation of SSp (for example) in vivo does not evoke dopamine release (or CIN activity or ACh release) in the dorsomedial striatum.

5. Considering the title and main conclusions refer broadly to “sensory cortices” and “sensory stimuli,” the limited scope of the in vivo experiments (visual and auditory only) should be acknowledged. Especially given that the effect of SSp stimulation on dopamine release in vivo was not tested (see point 4).

Minor:
1. Some findings that overlap or corroborate known cortical connections to MSNs and CINs should be cited (Johansson & Silberberg 2020; Assous & Tepper 2019).

2. Identification of some of the specific SSp body territories innervating the DLS seems to be at odds with prior work (e.g. SSp-m as the major DLS input- relevant experimental images lack clear mouth-specificity). It would probably be best to either avoid specifying the exact somatic body-map input territory, or clearly disambiguate the relevant SS subfields that do and do not project to DLS and whether they drive EPSCs in CINs and ACh/DA release.

3. The manuscript generally overstates the lack of connectivity between sensory cortex and CINs in the DLS. Considering Fig. 3 and S4 show both anatomical and functional connections between SSp and DLS CINs, line 140 (“…primary sensory areas do not synapse onto dorsal striatal CINs”) and other statements characterizing SSp input to CINS as “failing to produce any response” do not seem accurate and should be clarified.

4. Extra-striatal sources of ACh have not been directly tested or discussed. In Figs. 1, 2, 5, and 6, cholinergic afferents from the PPN and LDT could, in principle, contribute to GRAB-ACh, dLight, or FSCV signals. This concern is easy to address by citing work from others that has effectively ruled out the possibility (Brimblecombe et al. 2018).

5. In Figure 1 there is no panel “g”.

6. Figure 4 legend does not refer to panel “i”.

7. In general, it would help to indicate significance in all graphs, even if it’s stated in the legends. For example, an asterisk to indicate if DHBE significantly reduces DA concentration in figure 5f and others.

8. Page 8, line 240: should “Figure 2” and “Figure 3” be “Figure 3” and “Figure 4”?

9. Page 6, lines 192-193 states that mice were injected with ChR2-eYFP or ChrimsonR-tdTomato for slice electrophysiology experiments in figure 4. I may have missed it in the methods, but were recordings for each opsin balanced?

10. In Figure 4d and 4f it’s not clear if the representative traces (for cortex to MSN and cortex to CIN) are both 1 ms stim

duration, or 1 ms for MSNs and 10 ms for CINs, as stated in the text. If those traces from CINs are 10 ms, the visual marker for the stimulation should be lengthened.

11. Page 3, lines 73-74: the word “directly” is repeated.

12. Line 132: “prevailed” has a loaded connotation and should be replaced with “persisted”.

13. Line 490: “optogentic” should be “optogenetic”.

Reviewer #3

(Remarks to the Author)
Goldbach and colleagues investigated whether distinct cortical inputs to cholinergic interneurons (CINs) can drive acetylcholine (ACh)-dependent dopamine (DA) release in the dorsomedial striatum (DMS) via nicotinic receptor (nAChR) activation on dopaminergic terminals. Using a combination of in vivo fiber photometry, electrophysiology, selective optogenetic stimulation of glutamatergic inputs from various cortical regions, rabies retrograde tracing, and fast-scan cyclic voltammetry (FSCV), the authors propose that frontal cortices, but not sensory cortices, are strongly connected to CINs and capable of inducing local ACh-mediated DA release.
While connectivity from frontal cortices to CINs has been previously documented, the novelty of this study lies in demonstrating their ability to regulate local striatal DA release in contrast to sensory cortices. The manuscript is well-written and provides valuable insights, however, additional experiments and discussion are needed prior to its acceptance for publication to prevent overinterpretation of the current data and to strengthen the conclusions.

Major comments:
1)The authors suggest that DA transients evoked by visual stimuli (Figures 1 and 2) or optogenetic stimulation of prefrontal cortex projections (Figure 6) are cholinergic-dependent, based on their reduction following systemic administration of the nAChR blocker mecamylamine. However, this approach does not directly confirm this claim, as nAChRs are ubiquitously expressed throughout the brain, including midbrain DA neurons. The authors only partially controlled for potential disruption of visual pathways to the striatum, but to prove the proposed mechanism, further experiments are needed, specifically disrupting striatal cholinergic signaling, for example by selectively inactivating CINs/ACh release, or the nAChRs located on dopaminergic terminals, as done in previous studies (Krok et al., 2023; Chantranupong et al., 2023). Otherwise, the conclusions should be significantly tempered. Additionally, the authors should address why systemic mecamylamine completely abolished DA transients evoked by sensory stimuli (Figures 1h and 2m), suggesting a purely local origin of DA release, despite evidence that salient stimuli can activate DA neuron firing.

2)The current controversies regarding DA and ACh interactions in the striatum are mentioned in the Discussion, citing the studies that have reported anti-correlated signals in vivo. However, the authors have not made any comments or attempted to analyze the correlation between DA and ACh transients or compare their latencies in response to visual stimuli (Figures 1 and 2) or optogenetic activation of cortical projections (Figure 6).

3)The rationale for co-expressing ChR2 in the prelimbic cortex and ChrimsonR in sensory cortices for paired stimulation experiments (Figure S3) was nicely explained. However, it remains unclear which opsin was used for experiments examining the inputs from single cortical areas (Figure 4). Were animals injected with one opsin or the other per cortical area? Were the EPSCs pooled across opsins for the same cortical area? Or was ChR2 consistently used for frontal cortices and ChrimsonR for sensory cortices? This should be clarified, and the same opsin should have been consistently used for an appropriate comparison. Additionally, while the text mentions that both opsins were used for FSCV experiments, Figure 5 suggests only ChR2 was used.

4)The authors explored different durations of single-pulse optical stimulation in electrophysiology experiments (Figure 4). Given prior evidence that motor cortex inputs to CINs facilitate and can drive robust ACh release, it would be beneficial to examine different stimulation patterns and not just single pulses to validate these negative findings.

5)Why was a 5 ms optogenetic pulse used in FSCV experiments (Figure 5) to engage ACh-mediated DA release, when a 10 ms pulse was required to evoke detectable EPSCs in CINs (Figure 4)?

6)Although anatomical and functional experiments showed that frontal cortices but not sensory cortices (Vis, Aud, SSp) were primarily connected to CINs in the DMS, and no DA release was detected by FSCV, in vivo experiments would serve as important controls for these negative results. Specifically, examining CIN activation, ACh release, and DA release with single vs. multiple stimuli, as it was done for prefrontal cortex inputs (Figure 6).

7)Optogenetic stimulation of SSp-mj inputs to CINs in the DLS evoked detectable EPSCs (~80 pA, Figure S4d). Although the authors describe these as “smaller and more infrequent” (line 230), the frequency is not reported, and it appears all recorded CINs responded to single stimulation. This contrasts with the absence of EPSCs in DMS CINs (~0 pA). Despite the lack of DA release in ex vivo FSCV experiments upon SSp-mj terminals activation, it would be valuable to assess in vivo responses in the DLS and not just in the DMS.

Minor comments
Figure 3b – Include scale bar information in the legend.

Line 278 – Rephrase the heading because CINs cannot be “released”.
Materials and methods. Animals section. - Several mouse lines that were not used in the present study are mentioned.
Line 489 – Place “or” after the dose of mecamylamine and not before.

Reviewer #4

(Remarks to the Author)
This manuscript by Goldhach et al. investigated the circuit mechanisms underlying ACh-dependent DA release in the dorsomedial striatum (DMS). The authors combine anatomical tracing, in vivo fiber photometry, optogenetics, and ex vivo electrophysiology to demonstrate that frontal cortical inputs, but not sensory cortices, selectively drive cholinergic interneurons (CINs), leading to ACh-dependent DA release. Major findings include 1) light, but not auditory stimuli, triggers a DA transient that can be eliminated by i.p. injection of a nicotinic receptor antagonist; 2) CINs within the DMS prefer to receive inputs from frontal cortical areas; 3) stimulation of frontal cortical areas can trigger CIN firing and DA release both in vivo and ex vivo.

Major
1) It is unclear why the authors chose DMS to conduct the research, given that the striatum is a large brain region and functionally heterogeneous. Is the observed sensory-induced DA release unique to DA axons within the DMS? It is necessary to do a full characterization of the light-evoked DA release within other subregions of the striatum.
2) Please clarify the rationale for using head-fixed mice. Would similar responses be observed in a freely moving configuration?
3) The light intensity used in the study needs more justification. 5.9 cd/m^2 seems to be relatively dim. It is better to titrate light intensity and evoked response to clearly demonstrate their relationship. I wonder whether the response would be more complex as the light stimulation increases.
4) It is unclear why the ACh release evoked by sensory input in fig.2d-i (monophasic) is totally different from that in fig.6g-I (triphasic).
5) To demonstrate that sensory input is not jeopardized by systematic inhibition of nAChRs, the authors showed that the D1 MSNs can still respond to light input with or without mecamylamine. However, the experiment cannot rule out the possibility that the procedure does not influence the input to CINs, since MSNs and CINs receive different afferents (as shown in this study). Why not perform local nAChR inhibition at the recording site to more specifically address the point?
6) For circuit tracing and recording, the authors used a driver line (Chat-Cre) for specific labeling and manipulation of CINs. While this line works, it also suffers from significant ectopic recombination and mosaic expression. An immunostaining is needed to demonstrate the specificity of the labeling.
7) When measuring the functional connections, the author used both CHR2 and Chrimson to trigger the inputs from different cortices (fig.4). It is unclear which opsin is used for recordings in CINs and MSNs and why they must use two different opsins.
8) In Fig.5, the authors carried out an experiment where they triggered DA release using ChR2 injected at different brain regions and claimed that stimulation of the frontal cortex would evoke more DA release. Since there is no projection in the recording area (fig.5), it is not surprising that no DA release is evoked. It would be more interesting to test if DA release can be induced in regions with dense innervation of axons instead of only focusing on the DMS region. This is related to my comment 1), where I wonder whether DA axons innervating different subregions of the striatum are all regulated by CINs, but receive sensory input from distinct upstream brain regions.
9) Not sure what the S3 can tell us, given that 1) the response highly depends on the recording site. Recording made closer to the tdTomato-positive area will naturally receive more input from Chrimson-expressing axons and vice versa; 2) CHR2 stimulation (PFC) will inevitably recruit some Chrimson-positive fibers (sensory terminals), which explains why CHR2 stimulation always generates a larger response.
10) Why does ip administration of mecamylamine have very limited effect when ACh-DA is driven by optogenetic stimulation of the PFC (Fig. 6d-f vs Fig. 1e-f).
11) It is unclear how the evoked responses are quantified, especially when the traces include multiple phases (i.e., fig. 1e tone). It seems there should be a change in tone-induced response before and after nAChR blockade.
12) A loss-of-function study is required to demonstrate that the light-induced DA release is indeed caused by the PFC-CIN pathway.

Minor
Fig.1e figure legend should include a description for the scale bar.

Reviewer #5

(Remarks to the Author)
I co-reviewed this manuscript with one of the reviewers who provided the listed reports. This is part of the Nature Communications initiative to facilitate training in peer review and to provide appropriate recognition for Early Career Researchers who co-review manuscripts.

Version 1:

Reviewer comments:

Reviewer #1

(Remarks to the Author)
The authors satisfactorily addressed all of my comments, with many new experiments and analyses, and considerable modifications to the text. The revised manuscript presents a compelling story with strong evidence supporting exciting claims of broad interest. This study provides the best evidence to date of acetylcholine-evoked dopamine release in vivo; it ought to accepted for publication and will be cited abundantly as this is a hot topic with broad implications.

My only minor suggestion is that the author specify which variant of GCamp8 they use throughout the manuscript (including figures): I am guessing 8s, but this is not always specified.

Reviewer #2

(Remarks to the Author)
The authors have adequately addressed my concerns. I commend them on their exciting and important contribution!

Reviewer #3

(Remarks to the Author)
All points raised in the initial review have been adequately addressed. The additional experiments strengthen the conclusions. I recommend acceptance of the manuscript in its current form.

Reviewer #4

(Remarks to the Author)
The authors have done an excellent job in addressing my concerns, in most cases with experimental data. I have no further questions.

Figures 1b–d, j, 6c, and 7b. scale bar label/statement missing

In the rebuttal letter for reviewer #4, points 6) and 7) contain a strange statement that appears out of nowhere:
"The results from those experiments provide further support for our conclusions that sensory cortices do not have access to cholinergic-dependent dopamine signaling."

Reviewer #5

(Remarks to the Author)
I co-reviewed this manuscript with one of the reviewers who provided the listed reports. This is part of the Nature Communications initiative to facilitate training in peer review and to provide appropriate recognition for Early Career Researchers who co-review manuscripts.

Open Access This Peer Review File is licensed under a Creative Commons Attribution 4.0 International License, which permits use, sharing, adaptation, distribution and reproduction in any medium or format, as long as you give appropriate

credit to the original author(s) and the source, provide a link to the Creative Commons license, and indicate if changes were made.
In cases where reviewers are anonymous, credit should be given to 'Anonymous Referee' and the source.
The images or other third party material in this Peer Review File are included in the article's Creative Commons license, unless indicated otherwise in a credit line to the material. If material is not included in the article's Creative Commons license and your intended use is not permitted by statutory regulation or exceeds the permitted use, you will need to obtain permission directly from the copyright holder.
To view a copy of this license, visit https://creativecommons.org/licenses/by/4.0/

RESPONSE TO REVIEWER COMMENTS - NCOMMS-25-78202

We thank the reviewers for their thoughtful and constructive feedback. In response, we performed extensive new experiments, incorporated the resulting data, and restructured the manuscript to improve the clarity of both the methods and the interpretation. Following the reviewers' recommendations, the revised manuscript now focuses on the dorsomedial striatum, and the title has been revised accordingly. Below we summarize the major revisions before providing detailed point-by-point responses.

(1) Intracranial drug infusion strengthens support for proposed circuit mechanism: Several reviewers raised concerns about off-target effects of systemic antagonist administration. To address this, we performed new experiments using local intracranial infusion of nicotinic blockers. Local infusion robustly suppressed both visually-evoked and optogenetic-evoked dopamine responses, providing direct evidence that striatal acetylcholine locally regulates dopamine signaling. These new data in Figures 1 and 8 now replace the systemic administration data in the former Figures 1 and 6; the original systemic data have been moved to Supplemental Figures 1 and 7.

(2) Activity of dopamine neurons in response to visual stimuli: Reviewers also asked whether our findings could be instead explained by activation of midbrain dopamine neurons. We therefore added calcium recordings from midbrain dopaminergic cell bodies, dopaminergic axons in the striatum (revised Figure 1). These experiments showed that activation of dopamine neurons contributes to the signals but does not fully explain it. We revised the text and conclusions accordingly: Salient visual stimuli elicit striatal dopamine through both midbrain and local cholinergic mechanisms (line 97-124)

(3) Activity of prefrontal corticostriatal inputs in response to visual stimuli: To examine cortical contribution, we added calcium recordings from prefrontal corticostriatal inputs to the dorsomedial striatum during visual cue presentation. These axons responded strongly to light flashes (new Figure 6), supporting a role for prefrontal inputs in visually-evoked dopamine signaling and motivating the follow-up optogenetic and intracranial infusion experiments (Figures 7 and 8).

(4) Completing dataset on cholinergic interneurons: We also added the previously missing calcium recordings of striatal cholinergic interneuron and acetylcholine responses evoked by the tone to Supplemental Figure 3, which were previously missing.

(5) Improve clarity of methods: We expanded and clarified the methods, including the photometry analysis and the rationale for the dual-opsin experiments.

(6) Reorganized focus on the dorsomedial striatum: Based on the reviewers' comments, we substantially reframed the manuscript to focus on the dorsomedial striatum and its inputs. This revision is also consistent with a recent study published after our initial submission showing important differences in the dorsolateral striatum[1]. Accordingly, the *in vivo* data dorsolateral striatum has been moved to revised Supplemental Figure 5.

We thank the reviewers again for their helpful suggestions. The new experiments and extensive revisions address the major concerns and substantially strengthen the manuscript. We are confident that the revised study now offers compelling *in vivo* and *in vitro* evidence that

dopamine transmission in the dorsomedial striatum is regulated by a local cholinergic-dependent mechanism that can be recruited by frontal, but not sensory, cortical inputs.

POINT-BY-POINT RESPONSE

Reviewer #1:

This study from Goldbach and colleagues tackles a question of great interest in the field of basal ganglia research: how is the release of DA from axons regulated locally within the striatum by cholinergic interneurons (CINs)? This question has been the subject of many papers over the past 10 years, with good consensus amongst studies performed in rodent brain slices, but some disagreement with studies evaluating the prevalence of such mechanism in vivo. This manuscript describes experiments taking place both in slices and in vivo, all from mice. It makes a number of compelling observations: 1) salient sensory stimuli strongly drive DA release in the dorsomedial striatum (DMS) in vivo, 2) this DA release partially depends on nicotinic receptor signaling, 3) frontal cortico-striatal inputs provide strong excitatory drive onto DMS CINs in brain slices, but sensory cortico-striatal inputs do not, 4) stimulation of frontal cortico-striatal inputs drives CIN-dependent DA release in brain slices, but stimulation of sensory cortico-striatal inputs does not, and 5) optogenetic stimulation of frontal cortical axons is sufficient to drive striatal DA release in vivo, in a manner that is partially sensitive to nicotinic receptor blockade.

Overall, this study is of high quality: it is very clearly written, the references are adequate and the figures are crystal clear. The finding that frontal (but not sensory) cortico-striatal axons drive CINs (points 3 and 4 above) in brain slices is novel and ought to be of great interest to many. This study also provides a detailed retrograde tracing map of presynaptic inputs to CINs in different regions of dorsal striatum, which will be a great resource to the field (even if another similar experiment had been performed earlier, though not with the same attention to detail). The in vivo results are compelling too, and make a strong case that cortical drive can evoke DA release. The mechanisms involved, however, remain somewhat less clear. A case can certainly be made for the mechanism favored by the authors, but alternative possibilities are not excluded. As such, this study (in its current form) does not settle the debate in vivo. I still think that this study has great value and suggest experiments that, if performed, will greatly strengthen the manuscript, and if not, ought to be acknowledged and given ample space/discussion to help guide future experiments.

1) First, regarding the novel finding that sensory areas cortical afferents to striatum do not innervate CINs. It was not clear to me whether this reflects a novel finding that generalizes to all striatal regions (i.e. cortico-striatal afferents from sensory regions NEVER innervate CINs anywhere in striatum, but frontal cortico-striatal afferents do), or whether this reflects the choice made by the authors to focus their recordings in DMS. In other words, how much of this ‘specificity’ stems from differential innervation of striatum by cortical areas? Indeed, the retrograde tracing studies clearly make the case that the majority of cortico-striatal afferents to DMS originate in frontal cortices, not sensory, while

the opposite applies to more ‘sensory’ striatal regions such as DLS. Frontal cortical afferents are therefore unlikely to drive CINs in DLS either, which the title of the study does not reflect. This issue would greatly benefit from clarification throughout the text, and the title and conclusions should be modified to specify “in DMS”.

Thank you for this thoughtful and constructive comment. In response, we revised the manuscript to focus specifically on the dorsomedial striatum (DMS) and its cortical inputs. The text, figures, and title have all been updated accordingly to reflect this revised emphasis. In addition, the *ex vivo* dorsolateral striatum data have been moved to revised Supplemental Figure 7.

We have also clarified in the revised Methods and Results sections that recordings were obtained from regions with the densest cortical innervation. These revisions further strengthen our conclusion that sensory cortical areas do not evoke striatal dopamine release, even in regions where their projections are most dense.

2) On this same topic, other studies have reported cortically-evoked and CIN-dependent release of dopamine in DLS in brain slices when stimulating motor cortical axons, or thalamic axons. Assuming that sensory afferents are not capable of driving CINs anywhere in striatum, including in regions where their innervation pattern is densest, they would be the only afferents incapable of doing so. This should be the most exciting takeaway, with interesting molecular/developmental implications. Currently, the title and text seem to implicate a specialized role for frontal/executive cortical areas in controlling DA release, but this unlikely to be true, even in DMS where motor and thalamic afferents also exist (but their contribution was not investigated). Further refinements of the title, text and conclusions would therefore be needed for clarity.

We followed the reviewer’s suggestion by revising the title and corresponding text. The revised manuscript now also acknowledges that other inputs to the DMS capable of engaging CINs are likely to share similar properties and locally regulate dopamine signaling. For example, inputs from M1 and the intralaminar thalamus have been shown to evoke dopamine signals in vitro, a point we now state more clearly[2,3]. These issues are clarified and discussed on lines 375–377.

3) Second, regarding the in vivo experiments. A major limitation of this study is that the effects of systemic nAChR blockade are solely interpreted in the context of CIN-evoked DA release from axons locally within the striatum. While nAChRs do exist on DA axon terminals (and can drive DA release in slices, as show here and in many other studies), this is not the only site of action of nAChRs. They are, for example, strongly expressed on the soma and dendrites of DA neurons in the midbrain, which receive strong cholinergic inputs from pedunculopontine and laterodorsal tegmental nuclei (see PMID: 19523928, 24881574 for review). This contribution cannot be excluded. Figure 2 does a noble job of excluding gross alterations in sensory perception and the recruitment of dorsal striatal circuits, but the somatic response of midbrain DA neurons are the elephant in the room! As the debate between somatic vs axonal regulation of DA release in in vivo drags on, it

is no longer sufficient to ignore what happens in the cell body region. If the authors performed calcium imaging in the midbrain and 1) found light-evoked responses (which they certainly will) and 2) a substantial decrease in this response following systemic nAChR antagonist treatment (which they may), then the conclusions of this study would need to be revised, but the impact would remain, as there is a clear need to figure out what is goes on in vivo. The conclusions would be interesting and compelling in either case.

The reviewer raises several important concerns, and we thank them for these thoughtful suggestions. In response, we now address this central issue from two complementary angles: first, by using intracranial mecamylamine infusions to directly test local regulation of dopamine by striatal acetylcholine; and second, by performing calcium recordings from midbrain dopamine cell bodies and striatal dopamine axons. Together, these new experiments allow us to disentangle local intrastriatal regulation from any contribution of midbrain dopamine neuron activity to the observed dopamine signals.
First, to address the possibility that systemic mecamylamine affects cholinergic inputs to dopamine cell bodies outside the striatum, we performed a new set of experiments combining local drug delivery with fiber photometry. We expressed dLight1.3b in the dorsomedial striatum of C57BL/6J mice and implanted opto-fluid cannulas to infuse solutions directly at the recording site. Local infusion of mecamylamine into the striatum nearly abolished sensory-evoked dopamine signals, whereas saline had no effect. Importantly, the effect of local intrastriatal mecamylamine was much stronger than that of our previous systemic injections. These new data now replace the systemic results previously shown in Figure 1, which have been moved to Supplemental Figure 1.
Second, to directly examine the possible contribution of midbrain dopamine neurons, we expressed DIO-jGCaMP8s in the midbrain of DAT-Cre mice and recorded simultaneously from dopaminergic cell bodies in the midbrain and their axons in the dorsomedial striatum. To our surprise, we observed only very small responses to either light in both midbrain somata and striatal axons (Figure 1. Following systemic mecamylamine administration, the axonal response to light was slightly reduced, whereas the already weak responses in midbrain cell bodies were unaffected. These data are now presented in Supplemental Fig 1 and lines 115–120.
Taken together, these two new experiments provide strong evidence that the cholinergic component of the sensory-evoked dopamine response is mediated locally within the striatum, rather than arising indirectly from disrupted cholinergic input to dopaminergic cell bodies in the midbrain.

4) Figures 1 and 2 make the case that sensory stimuli drive DA release in striatum in a nAChR-dependent manner. At face value, this seems to run counter to the title and main point of the slice experiments, which show that sensory inputs do not drive CINs in DMS. The paper then dives into mechanisms local to striatum, but no attempt is made to loop back to this original set of experiments; we are left wondering how to interpret the findings. Is it the case that light flashes recruit frontal cortical areas, which go on to drive

DMS CINs, and DA release? Or is it a completely unrelated circuit, possibly originating in the midbrain cholinergic system? The value of these findings may be limited without any investigation of what happens in frontal cortical neurons, and most importantly, midbrain DA neurons.

We appreciate these insightful comments and the helpful suggestions for follow-up experiments. In response, we performed all of the suggested calcium photometry experiments to directly test both candidate pathways. Specifically, we added calcium photometry from frontal cortical axons in the striatum during cue presentation (new Figure 6), as well as calcium recordings from dopamine cell bodies and axons in the same animals described in Experiment 2 above (new Figure 1). In addition, to complete the circuit logic, we performed optogenetic stimulation of prefrontal corticostriatal inputs and showed that these inputs recruit striatal CIN activity in vivo and increase striatal acetylcholine levels (new Figure 7).
For the frontal cortex experiment, mice received unilateral injections of a jGCaMP8s viral vector into the prelimbic cortex and were implanted with bilateral fiber-optic cannulas in the DMS to record corticostriatal axonal activity. These axons responded strongly to light flashes (new Figure 6), supporting a role for prefrontal inputs in visually evoked dopamine signaling. Note, responses were stronger in the ipsilateral hemisphere to the injection, as expected.
These results motivated the follow-up optogenetic experiments showing stimulation of prefrontal corticostriatal projections recruited striatal CINs and elicited acetylcholine responses (Figures 7).
We also directly examined the possible contribution of midbrain dopamine neurons. Calcium recordings from dopaminergic cell bodies and axons showed small cue-evoked responses that were insensitive to nAChR blocker, indicating that midbrain activity may contribute but is unlikely to fully account for the observed dopamine signal. We therefore revised the manuscript to clarify that salient sensory stimuli engage striatal dopamine through both local cholinergic mechanisms and a more limited contribution from midbrain dopamine neurons. These new experiments resolve the apparent disconnect between the sensory-cue experiments and the slice data and substantially strengthen our interpretation of the *in vivo* circuit mechanism.

5) Figure 6 is the most direct test of the proposed model, but it still avoids the elephant in the room and glosses over details that directly speak to the prevalence of the proposed model in vivo:
   - A clear case is made that optogenetic stimulation of frontal cortex drives DA release in the striatum in proportion to pulse number. However, this response is barely affected by systemic nAChR block for 2 out of 3 pulse patterns, and not at all for the third, suggesting that nAChR signaling accounts for a minority of the DA released in this assay. Where does the larger nAChR-independent component come from?

These are important questions, and we addressed them in the revised manuscript with new experiments using intracranial antagonist infusion through dual opto-fluid cannulas.

Local infusion of the nicotinic receptor blocker mecamylamine reduced optogenetically evoked dopamine responses by 62%, indicating that a substantial portion of the signal is nAChR-dependent (new Figure 8).
Notably, intracranial blockade was more effective than systemic administration and suppressed responses across all stimulation conditions (line 328): single pulse, 62% ± 5.6%; 3 pulses, 61% ± 5.9%; and 10 pulses, 62% ± 6.7% reduction (treatment, p = 0.0003; stimulation intensity, p = 0.04; interaction, p = 0.15; two-way repeated-measures ANOVA).
The remaining component that was insensitive to nicotinic blockade may be mediated, at least in part, by muscarinic acetylcholine receptors. A recent study published during revision showed that muscarinic receptors also contribute to cholinergic regulation of dopamine in vivo[4]. Consistent with this possibility, muscarinic receptors are expressed on dopamine axons and are known to enhance dopamine release[5]. This is discussed in line 382 - 384.
Finally, it is important to note that in vivo optogenetic stimulation of cortical axons in the striatum may also activate passing fibers and axon collaterals that project to other targets. In particular, PL/ACA projections to the dorsomedial striatum also send collaterals to dopaminergic midbrain regions, which could contribute to part of the striatal dopamine signal measured in vivo[6]. We now discuss this point on line 372.
In summary, although this mechanism is unlikely to act in isolation, several of our new experiments provide strong evidence for a local circuit mechanism in the striatum that depends on CINs and acetylcholine.

6) DA release scales with stimulation intensity, but CIN and ACh responses do not.

This process likely involves nonlinear properties that we do not yet fully understand. The limited summation in measured acetylcholine signals may reflect saturation of the high-affinity GRAB-ACh sensor, which saturates rapidly. In contrast, the greater increase in dopamine release may arise from acetylcholine integrating over time and space to engage more distant and/or lower-affinity receptors. This interpretation is consistent with recent findings from Flink et al. (2025), who used ultrafast imaging to identify two dopamine peaks following CIN activation, involving distinct acetylcholine receptor components, including high- and low-affinity nicotinic receptors as well as muscarinic receptors. We now consider these issues in the revised Discussion.
Finally, we note that dopamine was measured with dLight, a relatively low-affinity sensor that is well suited to detect signal summation. A less linear pattern of summation might have been observed had we used GRAB-DA, which has higher affinity for dopamine.

7) Single pulses drive DA release, but do not significantly engage CINs or ACh release (at short latencies) in DMS.

We appreciate the reviewer's insightful questions. The relative magnitude and timing of these sensor signals are difficult to compare directly because they depend strongly on the properties of each sensor, including affinity and kinetics.

At present, we can only speculate about the timing differences, although we expect that ongoing work in the field will soon clarify these mechanisms.
Importantly, a recent study examined the temporal relationship between acetylcholine and dopamine signals in vivo using precisely timed optogenetic stimulation of CINs combined with ultrafast dopamine imaging. That study identified a biphasic dopamine response to CIN activation[4]. The initial dopamine peak depended on β2-containing nicotinic receptors, which are high-affinity receptors expected to respond to even small increases in acetylcholine. Together, these findings support the idea that relatively small elevations in acetylcholine may be sufficient to evoke dopamine release, as observed with single-pulse stimulation. This is discussed in line 398.

8) Cortical stimulation evokes a delayed pause in CIN and dip in ACh followed by a pronounced (and even more delayed) burst in CIN activity and ACh release. This delayed component does not seem to affect DA release at all, further calling into question the importance of local nAChR-evoked DA release in vivo.

Once dopamine is released via cholinergic activation, there is a refractory window before a sizable response can be released again[7,8]. This window is on the order of multiple seconds. The second burst in cholinergic activity occurs during this window, and therefore, even though acetylcholine is binding receptors and activating dopamine axons, there is likely no dopamine to release.

9) No attempt is made to exclude the null hypothesis that cortical stimulation drives DA neuron activity in the midbrain in a manner that is mildly affected by systemic nAChR antagonist treatment. Anatomical evidence supports direct innervation of DA neurons by cortical neurons (PMID: 22681690).
The impact of this study would be greatly enhanced by performing calcium imaging of DA neurons in SNc/VTA in this task. But short of this, the authors ought to devote considerable space in their discussion to mention these important caveats and to suggest these and other future experiments that would be needed to get to the bottom of this important issue.

We are grateful for this important comment and experiment suggestion. The revised manuscript now directly addresses this null hypothesis. We carried out the suggested experiment and directly measured the response of midbrain dopamine neurons to sensory cues and the effect of nicotinic blockers. The data are shown in Figure 1 and Supplemental Figure 4, and the results described in lines 109 - 111. Briefly, cues produced small calcium signals in midbrain dopamine somas and also calcium signals in dopamine axons in the dorsomedial striatum. We interpret these findings as evidence that both local regulation of dopamine release and somatic activation of dopamine contribute to cue-evoked dopamine signals in the striatum. This conclusion is now presented in the text (lines 97).

MINOR COMMENTS:

10) In vivo N is specified for hemispheres and mice: which is used for statistical analyses? If hemispheres are used, then statistical tests ought to factor in that multiple hemispheres are taken from the same mouse (i.e. each N is not a statistically independent measure).

Thank you for pointing this out. We now clearly state n values to be the number of animals. Previously, we had treated each hemisphere as an independent instance, largely because these sensors were expressed virally, and variation between hemispheres (even in the same animal) can be affected by the quality of the injection and implant. To address the reviewer's point that multiple hemispheres from the same mouse are not independent, In the few cases where we have the same sensor expressed in both hemispheres within an animal, we have now averaged them to be one number. The statistical tests are now based on the number of mice, rather than the number of hemispheres.

11) Can the authors provide non-experts a sense for how loud or bright the sensory stimuli used in Fig 1 are? Might they be considered equally salient, or aversive, or startling? Could this be a factor in explaining the difference between light and sound stimuli?

We now provide more detailed information on the tone and light stimuli in the methods (lines 522-523 and lines 529 - 530) and outlined below. While we aimed at matching the salience of the stimuli and paid attention to avoid a startle response, it is hard to completely rule out differences in salience. We believe a more likely explanation for the discrepancy in response amplitude to a light flash versus a tone may be our recording location along the anterior-posterior axis, or the connectivity between the visual or auditory cortex and the frontal cortex.

The tone was produced by the speaker at 70 dB. This volume is comparable to the volume of normal conversation, and we have successfully used it in our labs to train mice on a decision-making task, without signs of aversive responses. The light flash was produced by three 16-lux LEDs on the ceiling, resulting in 5.9 cd/m2 illumination on the box's floor. This is comparable to a nightlight or natural light at dawn/dusk. We purposely used dim light flashes to avoid stimuli that would be aversive or startling.

12) I am surprised that animals (and DA responses) don't habituate to sensory stimuli that are not paired with rewards. There is evidence that sensory-evoked DA responses decrease with time under similar conditions (PMID: 28054919; 39415006). What may the difference be here?

We appreciate this comment. We do see evidence of habituation to the sensory stimuli over time, which is why we have limited our sessions to only five repetitions of each. For the in vivo pharmacology experiments, this protocol was repeated twice (once before and once after saline or drug), and the limited exposure maintained an adequate degree of novelty. This information is now noted in the methods (lines 520 - 521).

13) Line 354: why is it difficult to rule out the engagement of DA neurons following cortical stimulation? As shown here, photometric imaging of calcium is well within your reach.

Indeed, we followed the helpful advice from this reviewer and carried out the additional experiments, as described above.

14) Ref 23 and 32 are now published.

Thank you for catching this. We have updated these citations to reflect this.

15) Line 399: This study does not study ACh-mediated control over DA throughout learning either; this distinction should therefore not be made to highlight the value of this work over others'.

We have edited the text to be clearer. Our intention was not to highlight the value of this work over others, but to point out that aspects of the acetylcholine-dopamine interaction have yet to be explored before the community concludes that this mechanism has little purpose.

16) Line 404: Please clarify what is meant by 'proprietary' role of frontal cortex, and how ref. 52 (in prior sentence) implicating thalamic inputs in ACh-mediated DA release support such a 'proprietary' role.

We have edited the text to shift emphasis to glutamatergic inputs (including thalamus) rather than the frontal cortex specifically.

17) Methods: Why are Pvalb-Cre and Sst-Cre lines listed for 'MSN' recordings??

These lines were used because the original goal of the recording experiments was to record from these interneurons. However, the goal changed based on the results from the voltammetry experiments and recordings from MSNs became the primary goal. Note that these two lines of mice are on a C57B6/J background, as well as the other transgenic lines used. We have edited the text to make it clearer that while all CINs were recorded in ChAT-Cre x tdTomato mice, MSN recordings were conducted in a few different genotypes (Lines 213 - 215, Methods).

18) Methods: Why is 405 nm light delivered at a lesser power than 470 nm light, especially since 405 nm light barely generates any signal and penetrates tissue less well than 470 nm light? How is the 405 nm signal used to correct or clean up the 470nm signal? More details of how photometry signals are analyzed using these packages to obtain df/f values (delta from what?) and z-scored is important.

We edited the methods section to include these additional details. The 405nm light is used as a control channel to capture non-neuromodulator-dependent changes in fluorescence. Because our animals are headfixed, we have minimal movement artifacts, and this channel mostly captures large artifacts caused by rarer movement of the fiber optic patch

cords (for example, if the system gets bumped or a cord is jostled mid-recording). The 470nm channel captures these artifacts, as well as neuromodulator-dependent changes in fluorescence. The dF/F is calculated by subtracting the control channel from the signal channel and dividing by the control channel. This is a standard practice for fiber photometry experiments. We titrated the drive levels for the 405 and 470 nm LEDs so that the incoming readings had similar voltages and similar signal-to-noise ratios.

Reviewer #2:

The manuscript by Goldbach et al. examines the afferent-specificity of evoked CIN-mediated dopamine release in the dorsomedial striatum. Using a combination of in vivo imaging, circuit tracing, slice electrophysiology and neuromodulator biosensors/measurements the authors show that frontal cortical regions (in particular the anterior cingulate and prelimbic) can reliably evoke CIN-mediated dopamine release in the dorsomedial striatum, whereas sensorimotor regions cannot. This is due to heterogenous anatomical and functional connectivity of diverse cortical afferents to CINs in the dorsal striatum. This is an exciting and important study, with profound implications for both reinforcement learning and numerous dopamine-related disorders. In my opinion, a few issues should be addressed to strengthen the intriguing conclusions of this important study.

Major:

1. The authors show in figure 1 that CIN-dependent dopamine release is stimulus-specific in the dorsomedial striatum: visual stimuli evoke nAChR-dependent dopamine release, whereas auditory stimuli do not. The interpretation is that the auditory stimulus used does not engage CINs as efficiently as the visual stimulus does. In figure 2 the authors then nicely show how a visual stimulus promotes CIN activity and ACh and dopamine release. The auditory stimulus may not evoke dopamine release in the dorsomedial striatum, but does it promote CIN activity and ACh release? Mechanistically, this would be important to know.

Thank you for this question. We have now included these data in Supplemental Figure 4. We do not see strong acetylcholine or cholinergic calcium responses to the auditory tone.

2. Related to point 1, previous studies have shown that auditory stimuli can produce complex responses in CINs (or at least in "tonically active neurons") in non-human primates. How do the authors think these observations fit with the data in this manuscript? I don't think this needs to be addressed with new experiments, but a discussion would be helpful.

We appreciate the opportunity to connect our findings with this rich literature from nonhuman primates. If invited to speculate based on our findings in the rostral DMS, we predict that auditory stimuli may also produce responses in mouse CINs at more caudal

location in the DMS. We also expect that these tone responses in CINs would not be generated by activation of auditory cortex inputs, as connectivity to CINs was weak when tested in vitro. Thus, we anticipate that the findings with auditory stimuli in the caudal DMS would be similar to what we observed with light stimuli in the rostral DMS.

We have edited the text to make the location of the recording clearer and noted that animals were naive to the stimuli. It is worth pointing out that the noted non-human primate studies recorded responses in trained animals where tones were predictive of outcomes and it is hard to know if similar results would be seen in tone-naive animals.

> 3. For slice electrophysiology experiments in figure 4, "CIN and MSN recordings were made from the striatal region receiving labeled projections." This makes sense, but it does not directly translate to the general question of what is going on in the dorsomedial striatum. For example, while since SSp axons are mostly found in the dorsolateral striatum, were any CINs recorded in response to SSp stimulation in the dorsomedial striatum, the region where ACh and dopamine release measures were made? I understand that the expectation is that responses would be very small if axon density is low, but this detail is central to the paper's conclusions.

The reviewer raises an important point. We now clarify that the focus of this study is the DMS. This issue was confusing to other reviewers as well so the revised manuscript clarifies the recording sites and limits the scope to focus on the dorsomedial striatum. Thus, the revised manuscript focuses on cortical areas that project solely to the DMS: ACA, PI, visual and auditory and carries out the in vitro and in vivo recordings in DMS. The data from SSp stimulation is reported in Supplemental Figure 10.

All experiments with SSp stimulation, either in vitro or in vivo, were carried out in the DLS. We cannot rule out that sparse SSp innervation to DMS may recruit CINs and evoke DA. However, this possibility seems highly unlikely based on our finding with other sensory areas that project to CINs and the rabies tracing that did not show labeling to CINs in DMS.

We found that CIN responses to SSp stimulation were already very small in the dorsolateral striatum, even though the dorsolateral striatum receives dense inputs from SSp. Consequently, we did not record from CINs in the dorsomedial striatum with SSp stimulation, since the dorsomedial striatum receives no projections from SSp. To resolve these points and improve the clarity of the paper, the revised manuscript now focuses on the DMS and the visual/auditory inputs that project heavily to DMS. The SSp data is still included, but has been moved to a Supplemental Figure 7.

> 4. The authors nicely show in figure 6 that stimulation of PL/ACA inputs evokes CIN-dependent dopamine release in the dorsomedial striatum in vivo (and in earlier figures show that anatomical/functional connectivity of sensory and motor cortex to CINs in this region is minimal). A useful control in figure 6 would be to show that stimulation of SSp

(for example) in vivo does not evoke dopamine release (or CIN activity or ACh release) in the dorsomedial striatum.

We have added new data to a supplemental figure (Supplement Figure 10) associated with Figures 7 and 8, which presents recordings of dopamine responses (and acetylcholine / cholinergic calcium responses) to SSp stimulation in the DLS. These new data show that SSp stimulation did not evoke responses in DLS; based on that result, we also would expect no responses in DMS (which receives no SSp input).

5. Considering the title and main conclusions refer broadly to “sensory cortices” and “sensory stimuli,” the limited scope of the in vivo experiments (visual and auditory only) should be acknowledged. Especially given that the effect of SSp stimulation on dopamine release in vivo was not tested (see point 4).

We agree with the reviewer’s point and have revised the manuscript to focus on the DMS and the auditory/visual projections to this region. This shift in focus is reflected in the title and throughout the paper. However, please note that we still include data from DLS and SSp stimulation (Supplemental Figures 7 and 9) even though this is not the focus of our study.

Minor:
1. Some findings that overlap or corroborate known cortical connections to MSNs and CINs should be cited (Johansson & Silberberg 2020; Assous & Tepper 2019).

Thank you for catching this major oversight. These papers were both major sources of inspiration for us. We believe these citations may have been accidentally removed during editing, and we have added these citations back in (Lines 63-65).

2. Identification of some of the specific SSp body territories innervating the DLS seems to be at odds with prior work (e.g. SSp-m as the major DLS input- relevant experimental images lack clear mouth-specificity). It would probably be best to either avoid specifying the exact somatic body-map input territory, or clearly disambiguate the relevant SS subfields that do and do not project to DLS and whether they drive EPSCs in CINs and ACh/DA release.

We have restructured the paper to focus more specifically on the dorsomedial striatum. Data from the dorsolateral striatum (except the rabies data) have been moved to Supplemental Figures 7 and 10, and we have combined the SSp-m data with the other SSp data.

3. The manuscript generally overstates the lack of connectivity between sensory cortex and CINs in the DLS. Considering Fig. 3 and S4 show both anatomical and functional connections between SSp and DLS CINs, line 140 (“…primary sensory areas do not

synapse onto dorsal striatal CINs") and other statements characterizing SSp input to CINS as "failing to produce any response" do not seem accurate and should be clarified.

As mentioned above, we have restructured the paper to focus on the dorsomedial striatum. We acknowledge that there are important differences between the dorsomedial and dorsolateral striatum. It is beyond the scope of our paper to fully document those differences, which is why we now restrict our main conclusions to the role of CINs and ACh in the dorsomedial striatum.

4. Extra-striatal sources of ACh have not been directly tested or discussed. In Figs. 1, 2, 5, and 6, cholinergic afferents from the PPN and LDT could, in principle, contribute to GRAB-ACh, dLight, or FSCV signals. This concern is easy to address by citing work from others that has effectively ruled out the possibility[9].

Thank you for pointing this out. We now include this in the discussion and cite work from others demonstrating that extra-striatal acetylcholine does not generally contribute to this mechanism (Lines 393 - 396).

5. In Figure 1 there is no panel "g".

Fixed.

6. Figure 4 legend does not refer to panel "i".

Fixed.

7. In general, it would help to indicate significance in all graphs, even if it's stated in the legends. For example, an asterisk to indicate if DHBE significantly reduces DA concentration in figure 5f and others.

We previously used markers beneath the x-axis labels to indicate significance in this figure. We have moved these markers above the data to make it clearer and more consistent with other figures. We have also ensured that other figures have the necessary significance markers.

8. Page 8, line 240: should "Figure 2" and "Figure 3" be "Figure 3" and "Figure 4"?

Fixed

9. Page 6, lines 192-193 states that mice were injected with ChR2-eYFP or ChrimsonR-tdTomato for slice electrophysiology experiments in figure 4. I may have missed it in the methods, but were recordings for each opsin balanced?

Yes, recordings of Figure 4 in the revised manuscript were made with both opsins. We now point this out in lines 209-210, with more details in the Methods (Lines 579 - 580), and the caption associated with Supplemental Figure 6.

10. In Figure 4d and 4f it's not clear if the representative traces (for cortex to MSN and cortex to CIN) are both 1 ms stim duration, or 1 ms for MSNs and 10 ms for CINs, as stated in the text. If those traces from CINs are 10 ms, the visual marker for the stimulation should be lengthened.

In this figure, only the VISp and AUDp traces were recorded with a 10 ms stimulation, to demonstrate that there was still no response even with a longer stimulation duration. We have adjusted the visual markers to reflect this, and please note that they are not to scale; a 1 ms marker would be nearly invisible, since the x scale spans 520 ms.

11. Page 3, lines 73-74: the word "directly" is repeated.

Fixed

12. Line 132: "prevailed" has a loaded connotation and should be replaced with "persisted".

Fixed

13. Line 490: "optogentic" should be "optogenetic".

Fixed

Reviewer #3:

Goldbach and colleagues investigated whether distinct cortical inputs to cholinergic interneurons (CINs) can drive acetylcholine (ACh)-dependent dopamine (DA) release in the dorsomedial striatum (DMS) via nicotinic receptor (nAChR) activation on dopaminergic terminals. Using a combination of in vivo fiber photometry, electrophysiology, selective optogenetic stimulation of glutamatergic inputs from various cortical regions, rabies retrograde tracing, and fast-scan cyclic voltammetry (FSCV), the authors propose that frontal cortices, but not sensory cortices, are strongly connected to CINs and capable of inducing local ACh-mediated DA release.
While connectivity from frontal cortices to CINs has been previously documented, the novelty of this study lies in demonstrating their ability to regulate local striatal DA release in contrast to sensory cortices. The manuscript is well-written and provides valuable insights, however, additional experiments and discussion are needed prior to its acceptance for publication to prevent overinterpretation of the current data and to strengthen the conclusions.

Major comments:

1)The authors suggest that DA transients evoked by visual stimuli (Figures 1 and 2) or optogenetic stimulation of prefrontal cortex projections (Figure 6) are cholinergic-dependent, based on their reduction following systemic administration of the nAChR blocker mecamylamine. However, this approach does not directly confirm this claim, as nAChRs are ubiquitously expressed throughout the brain, including midbrain DA neurons. The authors only partially controlled for potential disruption of visual pathways to the striatum, but to prove the proposed mechanism, further experiments are needed, specifically disrupting striatal cholinergic signaling, for example by selectively inactivating CINs/ACh release, or the nAChRs located on dopaminergic terminals, as done in previous studies (Krok et al., 2023; Chantranupong et al., 2023). Otherwise, the conclusions should be significantly tempered. Additionally, the authors should address why systemic mecamylamine completely abolished DA transients evoked by sensory stimuli (Figures 1h and 2m), suggesting a purely local origin of DA release, despite evidence that salient stimuli can activate DA neuron firing.

We agree with the reviewer's points, which were also raised by other reviewers. To address the issue of striatal cholinergic signaling, we performed additional experiments that tested local intracranial infusion of nicotinic blockers on sensory and optogenetically evoked dopamine responses. The results from these new experiments replace the mecamylamine/dopamine data in both Figures 1 and 7 and the previous, systemic data have been moved to Supplemental Figures 1 and 7. The new results provide much stronger support for our previous conclusions. Notably, the optogenetically evoked responses were significantly reduced by a local infusion of mecamylamine (62% reduction at the highest stimulation level vs 0% by systemic injection). These results from local intrastriatal application provide direct support for the idea of intrastriatal regulation of dopamine responses, and avoid indirect effects on midbrain and other brain regions from systemic mecamylamine.
To address the issue of midbrain DA neuron firing, we have added calcium recordings of midbrain dopaminergic cell bodies, midbrain dopaminergic axons in the striatum, and prefrontal cortical axons in the striatum (Figures 1 and 6). To our surprise, and probably to the surprise of Reviewer 1, novel sensory cues produced only small responses in both dopaminergic cell bodies and axons

2)The current controversies regarding DA and ACh interactions in the striatum are mentioned in the Discussion, citing the studies that have reported anti-correlated signals in vivo. However, the authors have not made any comments or attempted to analyze the correlation between DA and ACh transients or compare their latencies in response to visual stimuli (Figures 1 and 2) or optogenetic activation of cortical projections (Figure 6).

We agree that this is an interesting analysis and we have carried out comparisons on the rising phase of the dLight signal and the GCaMP signal in CINs to find a 40 ms delay between them, with GCaMP in CINs rising first and dLight signals following 40 ms later.

We limited the comparison to dLight and GCaMP because they have similar ON kinetics (~10 ms). GRAB-ACh has an ON tau of 88ms, which complicates direct comparisons with a much faster on signal such as dLight. Furthermore, direct comparisons on timing are also complicated by the fact that the recordings were done in different hemispheres of the same animal, since it's currently not possible to combine dual sensors with an opsin stimulation all in one hemisphere.
Thus, while direct comparisons of DA and ACh signal timing are not possible, comparisons between GCaMP8s and dLight, which share similar on-kinetics, revealed GCaMP in CINs rises first, and dLight follows about 40ms behind upon cue light presentation.
Fortunately, the main conclusions from our study do not depend on the timing or correlations between the recordings. We agree that in future work it would be interesting to use timing to gain additional insights into the circuits mechanisms, and for that work, we would use GRAB-DA instead of dLight.

To summarize, our experiments were not designed to directly compare the timing of recorded DA and ACh signals. Nonetheless, we did compare the onset times between GCaMP8s and dLight, since their on-kinetics are fairly similar. When we look at the cue-evoked responses, GCaMP in CINs rises first, and dLight follows about 40ms behind it.

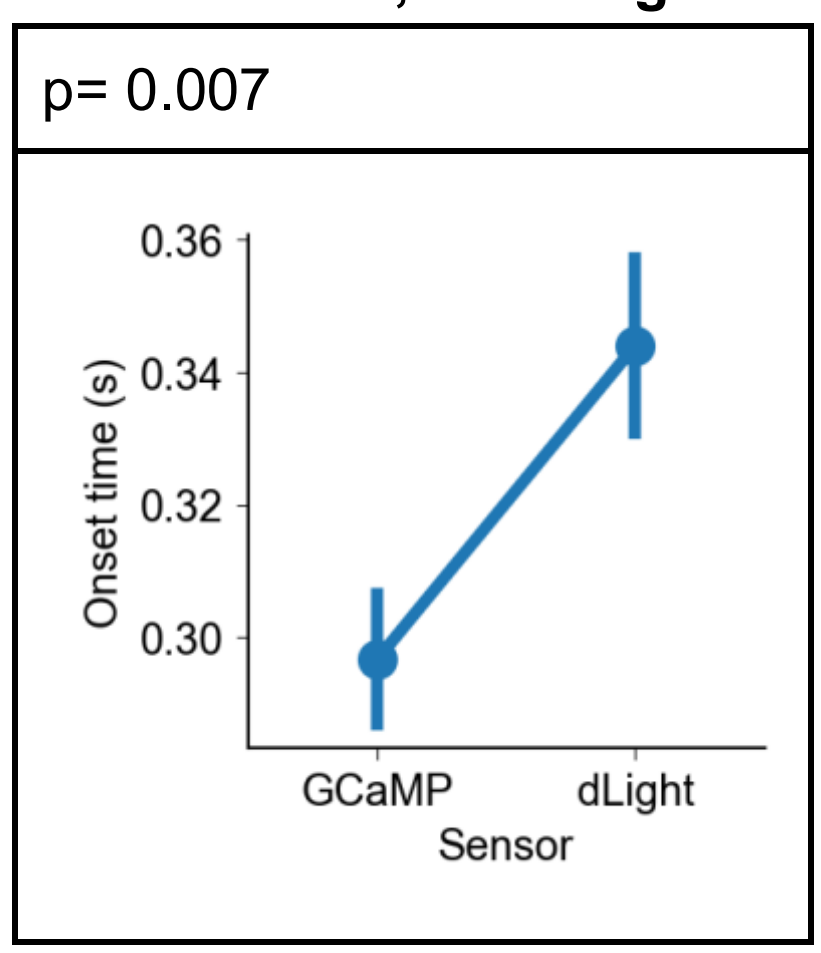

3)The rationale for co-expressing ChR2 in the prelimbic cortex and ChrimsonR in sensory cortices for paired stimulation experiments (Figure S3) was nicely explained. However, it remains unclear which opsin was used for experiments examining the inputs from single cortical areas (Figure 4). Were animals injected with one opsin or the other per cortical area? Were the EPSCs pooled across opsins for the same cortical area? Or was ChR2 consistently used for frontal cortices and ChrimsonR for sensory cortices? This should be clarified, and the same opsin should have been consistently used for an appropriate comparison. Additionally, while the text mentions that both opsins were used for FSCV experiments, Figure 5 suggests only ChR2 was used.

These issues are now clarified, and we have done additional experiments to verify that the choice of sensor does not change the results. Originally, we had injected ChrimsonR into sensory areas and ChR2 into frontal areas to allow paired recordings (Supplemenal Figure 6). We have now repeated the recordings with only ChR2 in sensory areas and replicated the results.

Briefly, the amplitudes of EPSCs recorded in CINs remained under 50 pA, regardless of the opsin used for stimulation. For MSNs, we found that using ChrimsonR tended to produce slightly larger responses than ChR2, regardless of the cortical area targeted. These results show that the connectivity pattern for cortical areas with CINs and MSNs holds constant regardless of the choice of opsin.

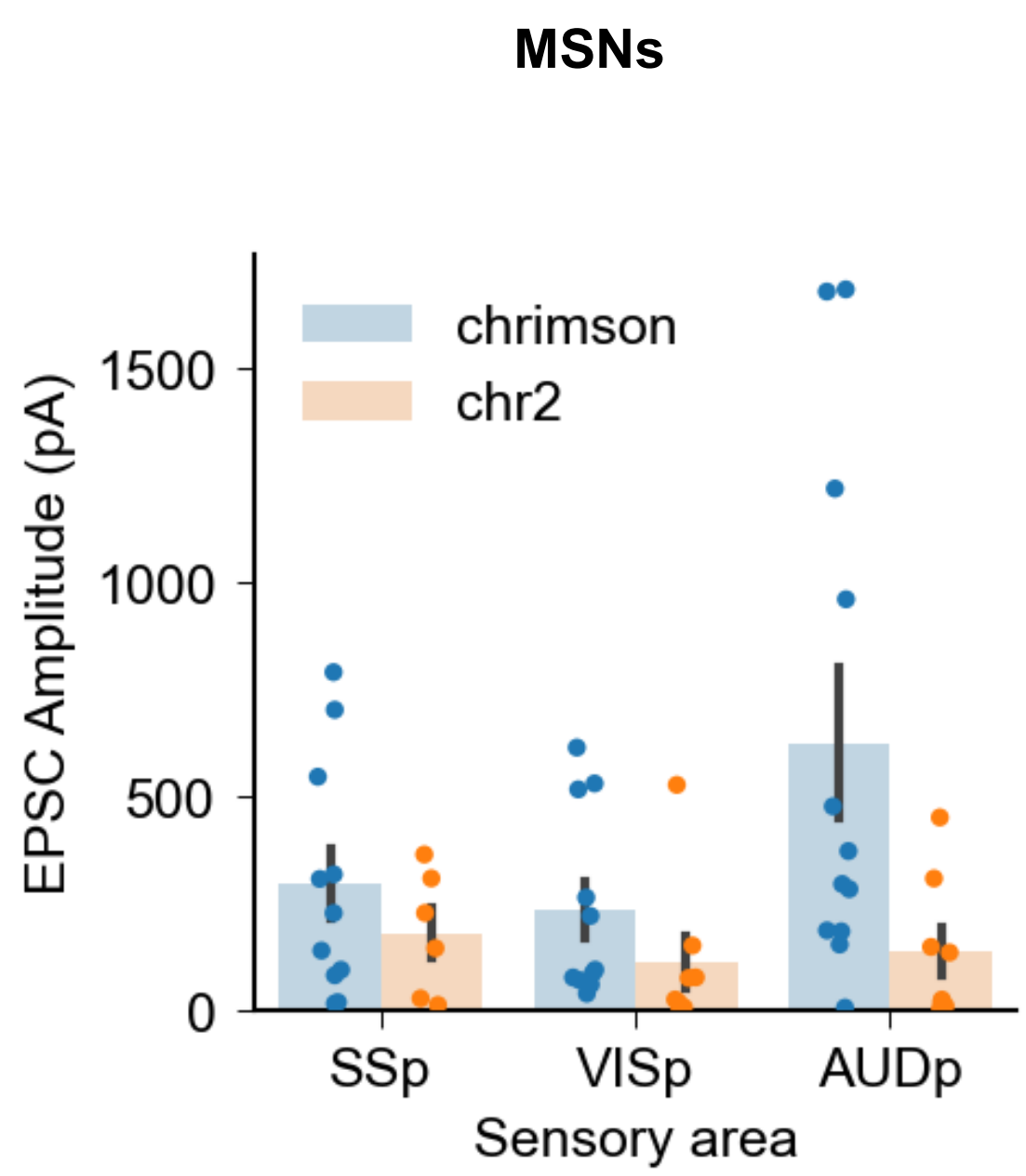

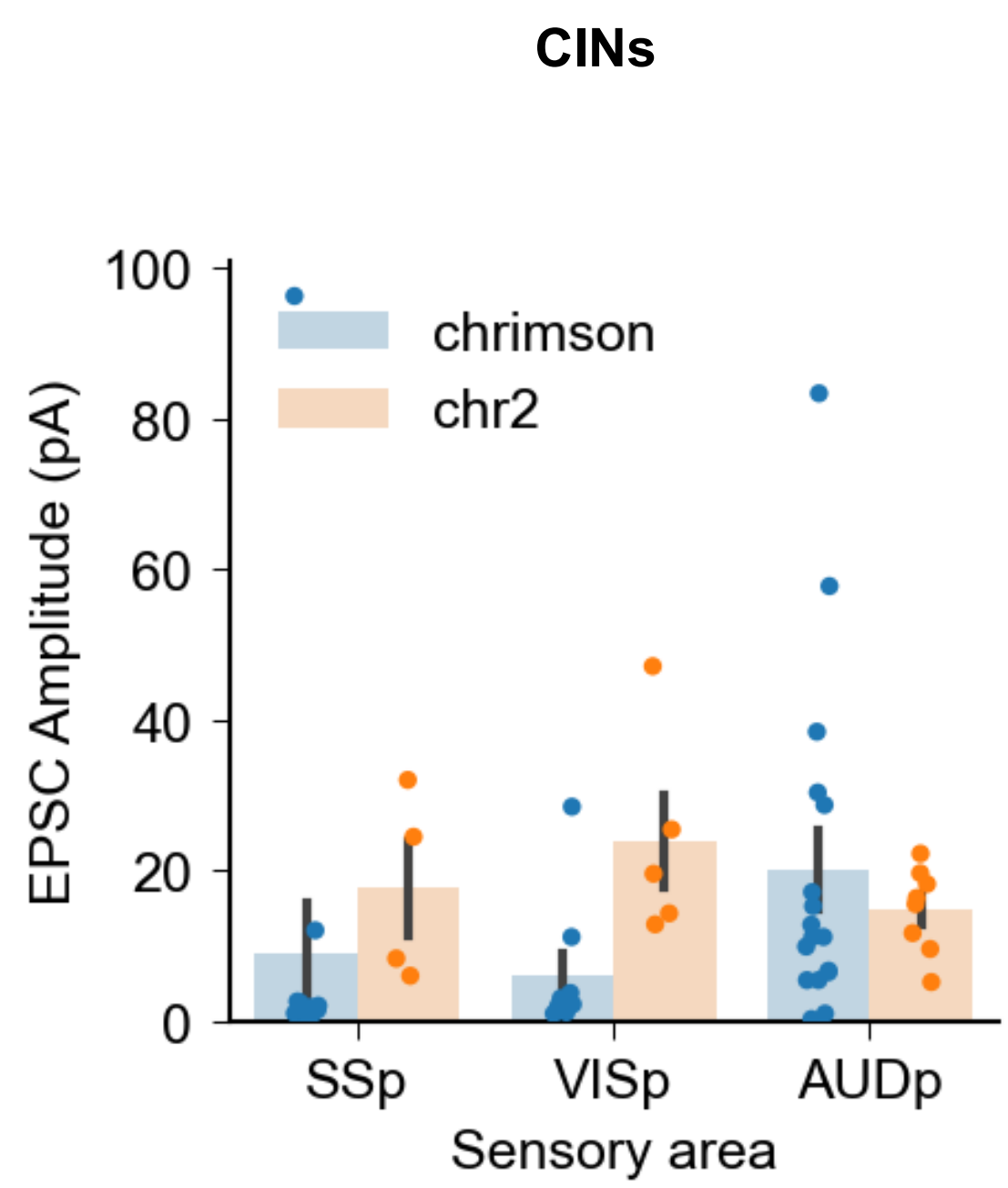

4)The authors explored different durations of single-pulse optical stimulation in electrophysiology experiments (Figure 4). Given prior evidence that motor cortex inputs to CINs facilitate and can drive robust ACh release, it would be beneficial to examine different stimulation patterns and not just single pulses to validate these negative findings.

This is an interesting question and an exciting follow-up experiment for understanding the short and long term plasticity changes triggered by high vs low frequency stimulation. For the revisions of this study, we focused on adding new experiments that were critical for strengthening the current conclusions and avoided expanding onto new directions.

5) Why was a 5 ms optogenetic pulse used in FSCV experiments (Figure 5) to engage ACh-mediated DA release, when a 10 ms pulse was required to evoke detectable EPSCs in CINs (Figure 4)?

Thank you for the opportunity to clarify this point. Our standard approach was to begin with a lower stimulation intensity and increase it until we were confident that no response would be detected. In the electrophysiology and fast-scan cyclic voltammetry experiments, the cutoff used to define a negative response differed because the two methods have different sensitivities. We now clarify in the Methods (lines 641–644) that both cutoff values are above the threshold at which we would expect to detect a response. As shown by the input-output curves in Figure 4, if a 5 ms stimulus does not evoke a response in CINs, a 10 ms stimulus also does not.

6)Although anatomical and functional experiments showed that frontal cortices but not sensory cortices (Vis, Aud, SSp) were primarily connected to CINs in the DMS, and no DA release was detected by FSCV, in vivo experiments would serve as important controls for these negative results. Specifically, examining CIN activation, ACh release, and DA release with single vs. multiple stimuli, as it was done for prefrontal cortex inputs (Figure 6).

As suggested by the reviewer, we now include data showing the effects of stimulating SSp inputs in the dorsolateral striatum on dLight, CIN GCaMP, and GRAB-ACh signals (Supplemental Figure 10). Although SSp stimulation elicited responses in CIN GCaMP and GRAB-ACh, it did not produce positive dLight responses. These findings provide additional support for our conclusion that sensory cortices do not engage cholinergic-dependent dopamine signaling.

7) Optogenetic stimulation of SSp-mj inputs to CINs in the DLS evoked detectable EPSCs (~80 pA, Figure S4d). Although the authors describe these as “smaller and more infrequent” (line 230), the frequency is not reported, and it appears all recorded CINs responded to single stimulation. This contrasts with the absence of EPSCs in DMS CINs (~0 pA). Despite the lack of DA release in ex vivo FSCV experiments upon SSp-mj terminals activation, it would be valuable to assess in vivo responses in the DLS and not just in the DMS.

In line with the reviewer’s suggestion, we have added new experiments showing in vivo recordings of dopamine, acetylcholine, and cholinergic calcium in the dorsolateral striatum (Supplemental Figure 10). As noted above in response to other reviewers, the revised manuscript now focuses on the dorsomedial striatum. This focus helps sharpen the main conclusions of the study—namely, the role of CINs in the local regulation of dopamine transmission—while acknowledging that important differences likely exist

between dorsomedial and dorsolateral striatum that are beyond the scope of the present work. Accordingly, we now acknowledge this point in the text (lines 328–330) and present the new dorsolateral striatum data in Supplemental Figures 7 and 10.

Minor comments

Figure 3b – Include scale bar information in the legend.

Fixed

Line 278 – Rephrase the heading because CINs cannot be “released”.

Fixed

Materials and methods. Animals section. - Several mouse lines that were not used in the present study are mentioned.

This has now been clarified in both the Results (lines 214–216) and the Methods. All mice used in this study were on a C57BL/6J background. We also now explain the rationale for using ChAT-Cre × tdTomato mice to label CINs for recording. MSN recordings were performed in the same mice, as well as in Pvalb-Cre and SST-Cre lines, and these strains are now specified in the Methods.

Line 489 – Place “or” after the dose of mecamylamine and not before.

The dosing referred to the control with saline administration (10 mL/kg). We have edited the text to make both the mecamylamine and saline dosing clearer.

Reviewer #4:

This manuscript by Goldhach et al. investigated the circuit mechanisms underlying ACh-dependent DA release in the dorsomedial striatum (DMS). The authors combine anatomical tracing, in vivo fiber photometry, optogenetics, and ex vivo electrophysiology to demonstrate that frontal cortical inputs, but not sensory cortices, selectively drive cholinergic interneurons (CINs), leading to ACh-dependent DA release. Major findings include 1) light, but not auditory stimuli, triggers a DA transient that can be eliminated by i.p. injection of a nicotinic receptor antagonist; 2) CINs within the DMS prefer to receive inputs from frontal cortical areas; 3) stimulation of frontal cortical areas can trigger CIN firing and DA release both in vivo and ex vivo.

Major

1) It is unclear why the authors chose DMS to conduct the research, given that the striatum is a large brain region and functionally heterogeneous. Is the observed sensory-induced DA release unique to DA axons within the DMS? It is necessary to do a full characterization of the light-evoked DA release within other subregions of the striatum.

Thank you for these important questions. The rationale for focusing on the dorsomedial striatum (DMS) is now stated in the Introduction (lines 36–40). Briefly, the DMS receives convergent input from visual and frontal cortical areas, positioning it as an integrative hub for sensory and cognitive information. It has been implicated in sensorimotor control, perceptual decision-making, and the processing of novel and salient stimuli during goal-directed behavior.
We agree with the reviewer that there are important functional differences across striatal subregions. In response to this feedback, and to comments from the other reviewers, we revised the manuscript to focus specifically on the DMS. We also expanded the dorsolateral striatum data, which are now presented in Supplemental Figure 10. Accordingly, our conclusions are limited to the DMS, although similar principles may apply more broadly across striatal regions. More specifically, our results support the principle that selective corticostriatal projections—and potentially other inputs, such as thalamic afferents—can drive dopamine transmission in the DMS through local cholinergic-dependent circuits.

> 2) Please clarify the rationale for using head-fixed mice. Would similar responses be observed in a freely moving configuration?

The use of head-fixed mice allowed precise control over visual stimulus presentation, including timing, position, and viewing distance. In addition, because sensory processing is lateralized in the brain, the head-fixed preparation ensured consistent bilateral stimulus delivery across trials and animals.
We expect that responses would be more variable in freely moving animals. For example, on different trials an animal might view the stimulus directly, or predominantly through the left or right visual field. Even so, we anticipate that the circuit mechanisms identified here remain valid and relevant during unconstrained behavior.

> 3) The light intensity used in the study needs more justification. 5.9 cd/m^2 seems to be relatively dim. It is better to titrate light intensity and evoked response to clearly demonstrate their relationship. I wonder whether the response would be more complex as the light stimulation increases.

We now provide a more detailed justification (lines 538–541). Briefly, we used relatively dim light flashes to maximize the reliability of visually evoked responses while minimizing startle. Because the mice were dark-adapted in the recording chamber, we avoided brighter stimuli, which are well known to induce aversive responses in mice.

> 4) It is unclear why the ACh release evoked by sensory input in fig.2d-i (monophasic) is totally different from that in fig.6g-I (triphasic).

There is indeed variability in the dynamics of ACh release, which has puzzled us and others[10]. We speculate that the response variability is in part due to differences in somatic vs dendritic vs axonal contributions of the calcium signals in CINs. Given that CINs are sparse in the striatum, fiber placement will determine the exact contribution of somatic vs dendritic vs axonal signals to the overall signals and this would impact a time course of the calcium response.

We now make this point explicitly in the revised manuscript and we document the variability and include examples of both monophasic and multiphasic responses (Suppl. Figures 8.

> 5) To demonstrate that sensory input is not jeopardized by systematic inhibition of nAChRs, the authors showed that the D1 MSNs can still respond to light input with or without mecamylamine. However, the experiment cannot rule out the possibility that the procedure does not influence the input to CINs, since MSNs and CINs receive different afferents (as shown in this study). Why not perform local nAChR inhibition at the recording site to more specifically address the point?

We followed the reviewer's suggestion and performed these experiments. In the revised manuscript, we now test the effects of local intracranial infusion of nicotinic blockers on both sensory-evoked and optogenetically evoked dopamine responses (new Figure 1 and 8). These new data replace the mecamylamine/dopamine results in the main figures and systemic data have been moved to Supplemental Figures 1 and 9.
The new findings provide much stronger support for our conclusions. In particular, local infusion produced substantially greater inhibition of optogenetically evoked dopamine responses than systemic mecamylamine, providing more direct evidence for local, intrastriatal regulation of dopamine signaling.

> 6) For circuit tracing and recording, the authors used a driver line (Chat-Cre) for specific labeling and manipulation of CINs. The results from those experiments provide further support for our conclusions that sensory cortices do not have access to cholinergic-dependent dopamine signaling.

We appreciate this comment and we agree that ectopic expression was a big problem in the original ChAT-IRES-Cre line. However, this problem is limited to certain ChAT-Cre driver lines and newer lines show more stable, clean expression of cre recombinase[11]. The line used for this paper, the del-neo line, has resolved the misexpression issue, as validated by other labs. The exact line used in now described in Methods

> 7) When measuring the functional connections, the author used both CHR2 and Chrimson to trigger the inputs from different cortices (fig.4). The results from those experiments provide further support for our conclusions that sensory cortices do not have access to cholinergic-dependent dopamine signaling.

These issues are now clarified, and we performed additional experiments to confirm that the choice of sensor/virus does not affect the conclusions. In the original study, we injected ChrimsonR into sensory cortical areas and ChR2 into frontal areas to enable paired recordings (Supplemental Figure 6). The reviewer raised the important possibility that the absence of responses from sensory inputs might reflect differences between viruses rather than true circuit differences. To address this, we repeated the sensory cortex recordings using ChR2 alone and obtained the same results.
In the revised manuscript, most frontal cortex stimulation was performed with ChR2, whereas sensory cortex stimulation was tested with both ChrimsonR and ChR2. In summary, CIN responses remained small—generally below 50 pA—regardless of the opsin used. In MSN recordings, ChrimsonR tended to produce somewhat larger responses than ChR2. Overall, these data show that the pattern of results is consistent across opsins and does not depend on the viral construct used.

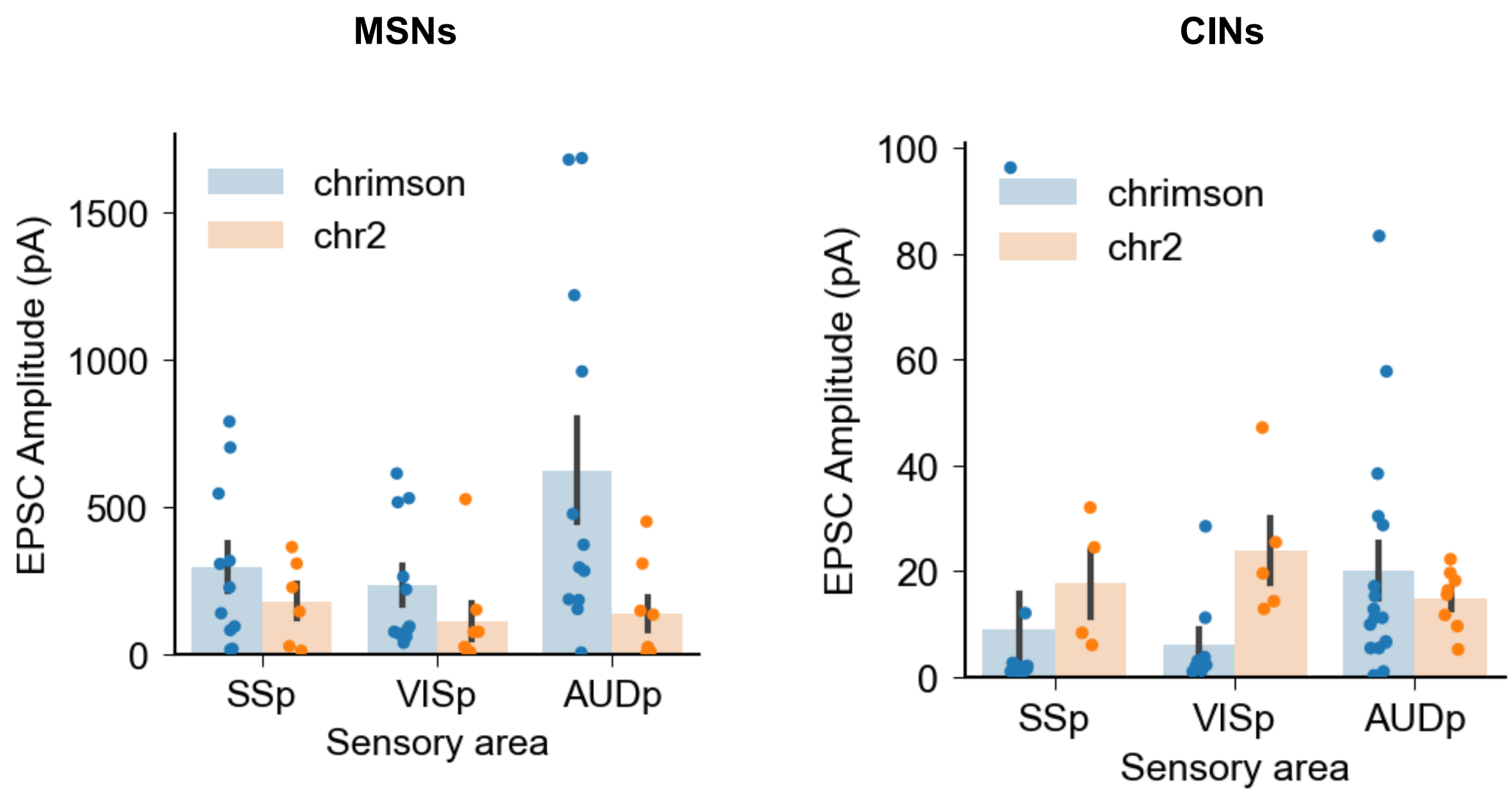

8) In Fig.5, the authors carried out an experiment where they triggered DA release using ChR2 injected at different brain regions and claimed that stimulation of the frontal cortex would evoke more DA release. Since there is no projection in the recording area (fig.5), it is not surprising that no DA release is evoked. It would be more interesting to test if DA release can be induced in regions with dense innervation of axons instead of only focusing on the DMS region. This is related to my comment 1), where I wonder whether

DA axons innervating different subregions of the striatum are all regulated by CINs, but receive sensory input from distinct upstream brain regions.

We have now clarified these points and confirmed that all recordings were obtained from striatal regions receiving dense axonal innervation from the targeted cortical area. The revised manuscript focuses on the dorsomedial striatum and its major cortical inputs: prelimbic, anterior cingulate, visual, and auditory cortex. Each of these regions densely innervates the dorsomedial striatum. The critical difference lies in their local targets. Sensory cortical inputs contact and drive MSNs, but they do not effectively engage CINs and therefore fail to evoke dopamine release. In contrast, frontal cortical inputs do engage CINs and are able to drive dopamine release. Together, these results support the principle that selected cortical inputs—and possibly other afferents—can regulate striatal dopamine release through local cholinergic mechanisms, distinct from direct activation of midbrain dopamine neurons.
Regarding the reviewer's final question, we speculate that dopamine axons in other striatal subregions are also regulated by CINs, based on prior published work[2].

9) Not sure what the S3 can tell us, given that 1) the response highly depends on the recording site. Recording made closer to the tdTomato-positive area will naturally receive more input from Chrimson-expressing axons and vice versa; 2) CHR2 stimulation (PFC) will inevitably recruit some Chrimson-positive fibers (sensory terminals), which explains why CHR2 stimulation always generates a larger response.

We apologize for the confusion about the recording site, which is now articulated and labeled more clearly in the revised manuscript. We confirm all recordings were performed in striatal subregions receiving projections. Suppl. Figure 3 has been revised and now only displays data from experiments targeting cortical areas with projections to the dorsomedial striatum (PL, ACA, Visual, Auditory).
The difference in the evoked DA signals cannot be explained by spurious activation of ChrimsonR expressing fibers. First, the difference is seen independently of the opsin used. Second, control experiments in slices only expressing ChrimsonR show no response when using 405 nm light under the same conditions used to stimulate axons. This control is now added to the Supplemental figure.

10) Why does ip administration of mecamylamine have very limited effect when ACh-DA is driven by optogenetic stimulation of the PFC (Fig. 6d-f vs Fig. 1e-f).

Additional experiments were done in the revised manuscript to improve drug delivery using intracranial infusions. The new data shows stronger inhibition by mecamylamine applied intracranially on PFC-driven DA signals in vivo (new Figure 8).
Systemic administration limited the dose that could be used. Mecamylamine and DHBE both can have rare adverse effects when administered systemically. With intracranial delivery, systemic adverse effects were less of a concern, and we were able to administer a high enough dose to eliminate both sensory and optogenetically-evoked responses. We

speculate that the reduced inhibition caused by systemic administration of nAChR blocker is due to low dose. This is now discussed in the revised manuscript (line 339-345).

11) It is unclear how the evoked responses are quantified, especially when the traces include multiple phases (i.e., fig. 1e tone). It seems there should be a change in tone-induced response before and after nAChR blockade.

We appreciate this comment. We investigated the effect of nAChR blocker on the tone response and found that intracranial mecamylamine suppressed the tone evoked dLight signals. This data is now presented in Supplemental Figure 2. We have also added details to the methods and new supplemental figures to help clarify how evoked responses were quantified (lines 554 - 558, Supplemental Figures 2 and 8).

12) A loss-of-function study is required to demonstrate that the light-induced DA release is indeed caused by the PFC-CIN pathway.

The revised manuscript includes new experiments validating the PFC->CIN-> local dopamine aspect of the proposed circuit mechanism. Recruitment of midbrain DA neuron, either axons and somas were measure calcium signals addressed the recruitment of midbrain dopamine neuron somas s by

Our aim was not to state definitively that these responses are caused by the PFC-CIN pathway, only that it is a more likely possibility (alongside thalamic routes) than inputs from sensory cortices. We have edited the text to reflect this. We have also added data to Figure 1 that includes recordings of PFC axons in the striatum, demonstrating that these axons do respond to sensory stimuli. This provides further evidence that it is a feasible pathway, though again, our intention is not to say for certain that this pathway is responsible - only that it is possible.

Minor

Fig.1e figure legend should include a description for the scale bar.

Done

References

1. Bouabid, S. *et al.* An anatomical hotspot for striatal dopamine-acetylcholine interactions during reward and movement. 2026.01.20.700614 Preprint at https://doi.org/10.64898/2026.01.20.700614 (2026).

2. Kosillo, P., Zhang, Y.-F., Threlfell, S. & Cragg, S. J. Cortical Control of Striatal Dopamine Transmission via Striatal Cholinergic Interneurons. *Cereb. Cortex N. Y. NY* **26**, 4160 (2016).

3. Cover, K. K. *et al.* Activation of the Rostral Intralaminar Thalamus Drives Reinforcement through Striatal Dopamine Release. *Cell Rep.* **26**, 1389-1398.e3 (2019).
4. Flink, D. R., Faturos, N. G., Zhang, H. & Hamid, A. A. Dual cholinergic mechanisms for sculpting striatal dopamine in vivo. 2025.12.19.695021 Preprint at https://doi.org/10.64898/2025.12.19.695021 (2025).
5. Shin, J. H., Adrover, M. F., Wess, J. & Alvarez, V. A. Muscarinic regulation of dopamine and glutamate transmission in the nucleus accumbens. *Proc. Natl. Acad. Sci. U. S. A.* **112**, 8124–8129 (2015).
6. Green, S. M., Nathani, S., Zimmerman, J., Fireman, D. & Urs, N. M. Retrograde Labeling Illuminates Distinct Topographical Organization of D1 and D2 Receptor-Positive Pyramidal Neurons in the Prefrontal Cortex of Mice. *eNeuro* **7**, (2020).
7. Brill-Weil, S. G. *et al.* Presynaptic GABAA receptors control integration of nicotinic input onto dopaminergic axons in the striatum. *Cell Rep.* **44**, 116555 (2025).
8. Kramer, P. F., Yanez, A., Clever, F., Zhang, R. & Khaliq, Z. M. High-frequency axonal bursts mediate bidirectional modulation of dopamine signaling by nicotinic receptors. 2025.12.19.695584 Preprint at https://doi.org/10.64898/2025.12.19.695584 (2026).
9. Brimblecombe, K. R. *et al.* Targeted Activation of Cholinergic Interneurons Accounts for the Modulation of Dopamine by Striatal Nicotinic Receptors. *eNeuro* **5**, (2018).
10. Bouabid, S. *et al.* Distinct spatially organized striatum-wide acetylcholine dynamics for the learning and extinction of Pavlovian associations. *Nat. Commun.* **16**, 5169 (2025).
11. Rossi, J. *et al.* Melanocortin-4 receptors expressed by cholinergic neurons regulate energy balance and glucose homeostasis. *Cell Metab.* **13**, 195–204 (2011).

RESPONSE TO REVIEWER COMMENTS - NCOMMS-25-78202A

We sincerely thank all of the reviewers for their thoughtful and constructive feedback throughout the review process. Their careful evaluations and insightful suggestions have greatly strengthened the manuscript and helped us present the study in its strongest form. We are grateful for the reviewers' positive assessment of the revised manuscript, and we share their view that this study will be of broad interest and significance to the field.

POINT-BY-POINT RESPONSE

Reviewer #1:

The authors satisfactorily addressed all of my comments, with many new experiments and analyses, and considerable modifications to the text. The revised manuscript presents a compelling story with strong evidence supporting exciting claims of broad interest. This study provides the best evidence to date of acetylcholine-evoked dopamine release in vivo; it ought to accepted for publication and will be cited abundantly as this is a hot topic with broad implications.

My only minor suggestion is that the author specify which variant of GCamp8 they use throughout the manuscript (including figures): I am guessing 8s, but this is not always specified.

We sincerely thank the reviewer for their very positive assessment of our revised manuscript and for their thoughtful and constructive comments throughout the review process. We are grateful that the reviewer found the additional experiments, analyses, and revisions to have strengthened the manuscript and that they view the study as providing compelling evidence for acetylcholine-evoked dopamine release in vivo. The reviewer's comments and suggestions were extremely helpful in improving the rigor, clarity, and overall impact of the study.

We have specified the GCaMP8 variants used throughout the manuscript. We have now updated the appropriate text and figures to clarify when GCaMP8s or GCaMP8f was used.

Reviewer #2:

The authors have adequately addressed my concerns. I commend them on their exciting and important contribution!

We are grateful for the reviewer's enthusiastic assessment of the revised manuscript and sincerely thank them for thoughtfulness throughout the review process.

Reviewer #3:

All points raised in the initial review have been adequately addressed. The additional experiments strengthen the conclusions. I recommend acceptance of the manuscript in its current form.

Thank you for the positive assessment of the revised manuscript.

Reviewer #4:

The authors have done an excellent job in addressing my concerns, in most cases with experimental data. I have no further questions.

Figures 1b–d, j, 6c, and 7b. scale bar label/statement missing

In the rebuttal letter for reviewer #4, points 6) and 7) contain a strange statement that appears out of nowhere:
"The results from those experiments provide further support for our conclusions that sensory cortices do not have access to cholinergic-dependent dopamine signaling."

We are delighted by this response and we sincerely thank the reviewer for their thoughtful and constructive comments throughout the reviewing process.

Scale bars have been added to the noted figure panels.

Reviewer #5:

I co-reviewed this manuscript with one of the reviewers who provided the listed reports. This is part of the Nature Communications initiative to facilitate training in peer review and to provide appropriate recognition for Early Career Researchers who co-review manuscripts.
